# The BIG protein distinguishes the process of CO_2_‐induced stomatal closure from the inhibition of stomatal opening by CO_2_

**DOI:** 10.1111/nph.14957

**Published:** 2018-01-02

**Authors:** Jingjing He, Ruo‐Xi Zhang, Kai Peng, Cecilia Tagliavia, Siwen Li, Shaowu Xue, Amy Liu, Honghong Hu, Jingbo Zhang, Katharine E. Hubbard, Katrin Held, Martin R. McAinsh, Julie E. Gray, Jörg Kudla, Julian I. Schroeder, Yun‐Kuan Liang, Alistair M. Hetherington

**Affiliations:** ^1^ State Key Laboratory of Hybrid Rice Department of Plant Sciences College of Life Sciences Wuhan University Wuhan 430072 China; ^2^ School of Biological Sciences Life Sciences Building 24 Tyndall Avenue Bristol BS8 1TQ UK; ^3^ Lancaster Environment Centre Lancaster University Lancaster LA1 4YQ UK; ^4^ College of Life Science and Technology Huazhong Agricultural University Wuhan 430070 China; ^5^ Cell and Developmental Biology Section Division of Biological Sciences University of California at San Diego La Jolla CA 92093 USA; ^6^ School of Environmental Sciences University of Hull Hull HU6 7RX UK; ^7^ Institut für Biologie und Biotechnologie der Pflanzen Universität Münster Schlossplatz 7 Münster 48149 Germany; ^8^ Department of Molecular Biology and Biotechnology University of Sheffield Firth Court, Western Bank Sheffield S10 2TN UK

**Keywords:** abscisic acid (ABA), *Arabidopsis thaliana*, *BIG* gene, CO_2_ signaling, stomatal function, S‐type anion channel

## Abstract

- We conducted an infrared thermal imaging‐based genetic screen to identify Arabidopsis mutants displaying aberrant stomatal behavior in response to elevated concentrations of CO_2_.
- This approach resulted in the isolation of a novel allele of the Arabidopsis *BIG* locus (*At3g02260*) that we have called *CO*_*2*_ *insensitive 1* (*cis1*).
- *BIG* mutants are compromised in elevated CO_2_‐induced stomatal closure and bicarbonate activation of S‐type anion channel currents. In contrast with the wild‐type, they fail to exhibit reductions in stomatal density and index when grown in elevated CO_2_. However, like the wild‐type, *BIG* mutants display inhibition of stomatal opening when exposed to elevated CO_2_. *BIG* mutants also display wild‐type stomatal aperture responses to the closure‐inducing stimulus abscisic acid (ABA).
- Our results indicate that BIG is a signaling component involved in the elevated CO_2_‐mediated control of stomatal development. In the control of stomatal aperture by CO_2_, BIG is only required in elevated CO_2_‐induced closure and not in the inhibition of stomatal opening by this environmental signal. These data show that, at the molecular level, the CO_2_‐mediated inhibition of opening and promotion of stomatal closure signaling pathways are separable and BIG represents a distinguishing element in these two CO_2_‐mediated responses.

## Introduction

Stomata consist of a pair of guard cells that surround a central pore and serve to regulate water loss and the uptake of CO_2_. Both the aperture of the stomatal pore and the number of stomata that develop on the leaf surface are controlled by environmental signals. By integrating external signals and local cues, stomata ‘set’ gas exchange to suit the prevailing environmental conditions (Hetherington & Woodward, 2003). One of the signals that controls stomatal aperture and influences stomatal development, in both the short and long term, is the atmospheric concentration of carbon dioxide ([CO_2_]) (Kim *et al*., 2010; Franks *et al*., 2012). In response to an increase in [CO_2_], stomatal aperture reduces, as, in general, do the number of stomata that develop on the surface of the leaves (Vavasseur & Raghavendra, 2005; Kim *et al*., 2010; Franks *et al*., 2012). Understanding how the plant perceives changes in [CO_2_] and integrates this information with other internal and external signals, resulting in the adjustments of stomatal aperture and density, is of key importance in the context of understanding the impact of global environmental change on plants (Assmann & Jegla, 2016).

Recently, we have begun to understand more about the underlying cellular mechanisms responsible for coupling increased [CO_2_] to reduced stomatal conductance (Kim *et al*., 2010; Assmann & Jegla, 2016; Engineer *et al*., 2016). In this context, it is important to recognize that elevated CO_2_‐induced reductions in stomatal conductance are the net result of two processes: the promotion of stomatal closure and the inhibition of stomatal opening (Assmann, 1993). These processes are separable; abscisic acid (ABA)‐induced stomatal closure is distinct from ABA‐inhibited stomatal opening (Allen *et al*., 1999; Wang *et al*., 2001; Mishra *et al*., 2006). However, before the current work, it was not known whether this also applied to [CO_2_]‐induced changes in stomatal aperture.

There is evidence that the guard cell ABA and CO_2_ signaling responsible for the inhibition of light‐induced stomatal opening pathways converge (Webb & Hetherington, 1997). It has been suggested that elevated [CO_2_] brings about its effects on stomatal aperture and development by accessing the ABA signaling pathway, because there is a requirement for both ABA and the ABA receptors of the PYR/RCAR family in these responses (Chater *et al*., 2015). There are other data suggesting that the early steps in CO_2_‐mediated closure converge with ABA signaling downstream of ABA receptors and the two pathways influence each other on convergence (Xue *et al*., 2011; Merilo *et al*., 2013; Hõrak *et al*., 2016; Jakobson *et al*., 2016; Yamamoto *et al*., 2016). Obviously, these processes are not mutually exclusive. Although the mechanism(s) through which the guard cell ABA signaling pathway is accessed is not fully understood, it has been possible to distinguish, on a genetic basis, components that function in CO_2_‐mediated closure, but not in guard cell ABA signaling. In Arabidopsis, these include β‐carbonic anhydrases which are encoded by the *CA1* and *CA4* genes (Hu *et al*., 2010), the protein kinase HT1 (HIGH LEAF TEMPERATURE 1) (Hashimoto *et al*., 2006), RHC1, a MATE transporter (Tian *et al*., 2015), and the MAP kinase MPK4 (Hõrak *et al*., 2016; Jakobson *et al*., 2016). Loss of the CAs, RHC1 and MPK4 impairs CO_2_‐induced closure (Hashimoto *et al*., 2006; Hu *et al*., 2010; Tian *et al*., 2015; Hõrak *et al*., 2016; Jakobson *et al*., 2016), whereas recessive *ht1* alleles show a constitutive high CO_2_ response (Hashimoto *et al*., 2006; Hashimoto‐Sugimoto *et al*., 2016).

In 1987, Woodward discovered an inverse relationship between atmospheric [CO_2_] and stomatal density (Woodward, 1987). We know less about the operation of this developmental signaling pathway, but the putative β‐keto acyl CoA synthase encoded by the *HIC* gene is involved, as are the CO_2_ Response Secreted Protease (CRSP), the β‐carbonic anhydrases CA1 and CA4, and the peptide Epidermal Patterning Factor 2 (EPF2) (Gray *et al*., 2000; Doheny‐Adams *et al*., 2012; Engineer *et al*., 2014). Most recently, it has been shown that the activity of the reactive oxygen species (ROS)‐producing NADPH oxidases, encoded by the *RBOHD* and *RBOHF* genes, is involved in the CO_2_‐mediated reduction in stomatal density, as is ABA and the ABA receptors encoded by the PYR/RCAR family (Chater *et al*., 2015).

During an infrared thermal imaging genetic screen in Arabidopsis (Wang *et al*., 2004), we isolated a novel allele of the *BIG* locus (*At3g02260*) that we name *CO*_*2*_ *insensitive 1* (*cis1*), which is compromised in both elevated [CO_2_]‐induced closure and reduction in stomatal density. However, when challenged with ABA, *cis1* displays reductions in stomatal aperture that are indistinguishable from the wild‐type (WT), suggesting that *BIG* (*CIS1*) functions upstream of ABA or in an ABA‐independent signaling pathway responsible for the control of stomatal aperture by CO_2_. We also found that the activation of the guard cell S‐type anion channel by bicarbonate is compromised by the loss of *BIG* function. Furthermore, in contrast with elevated [CO_2_]‐mediated closure, the ability of elevated [CO_2_] to inhibit stomatal opening was not affected in this mutant. In summary, we have identified BIG as a new component in the signaling pathway responsible for the control of stomatal development by elevated [CO_2_]. We also show that *BIG* features in the signaling pathway through which elevated [CO_2_] controls stomatal aperture. Importantly, we show that BIG is only involved in elevated [CO_2_]‐induced stomatal closure and is not involved in the inhibition of stomatal opening by this environmental signal or in stomatal responses to ABA. These results show that, at the molecular level, these pathways are separable, with BIG representing a component that distinguishes these two CO_2_‐mediated responses.

## Materials and Methods

### Plant growth

All Arabidopsis (*Arabidopsis thaliana* L.) lines used were in the Columbia background (Col‐0). Seeds of *doc1‐1* and *big‐1* were obtained from NASC (the European Arabidopsis Stock Centre, http://arabidopsis.org.uk). Seed germination and plant growth were performed as described previously (Liang *et al*., 2010).

### Mutant screen

To identify genes required for stomatal CO_2_ responses, we screened 20 000 seeds from an Arabidopsis EMS (ethyl methanesulfonate) M2 population representing 40 independent pools (each pool corresponding to *c*. 1000 M1 plants) by infrared thermal imaging (Wang *et al*., 2004; Xie *et al*., 2006). Screening was carried out on 3–4‐wk‐old plants in a purpose‐built chamber (84 × 68 × 20 cm^3^), located inside a controlled environment room. The CO_2_ concentration inside the chamber was controlled externally from CO_2_ cylinders. Air flow in the chamber was maintained at 0.03 m s^−1^ using fans. Relative humidity inside the chamber was *c*. 60%, temperature was 22°C and light intensity was 120 μmol m^−2^ s^−1^. Plants were placed in the chamber and exposed to 360 ppm [CO_2_] (360 ppm [CO_2_] cylinder (balanced air mixture)). After 40 min, thermal images were captured and the plants were then exposed to 1500 ppm [CO_2_] (1500 ppm [CO_2_] cylinder (balanced air mixture)) for a further 40 min, and thermal images were captured. Pairs of images were compared to identify putative CO_2_ response mutants. Infrared thermal imaging was performed using an Inframetrics middle infrared (3.4–5 μm) camera model SC1000E (FLIR Systems Inc., Wilsonville, OR, USA). Images were stored in a ThermaCam Image file format (IMG) and analysed with the ThermaCam™ Researcher 2001 software (FLIR Systems). Mutants exhibiting altered leaf thermal profiles compared with WT were selected, self‐pollinated, and seeds (M3) were collected for further investigation. Backcross seeds (F_1_s) were obtained using mutant lines as female and Col‐0 as male. F_2_ was used for segregation analysis. Mutants segregating in F_2_ were backcrossed to WT Col‐0 for another two generations before being used for fine mapping and phenotyping.

### Map‐based mutant gene cloning

*cis1* mutants were outcrossed to WT plants in the Landberg *erecta* background (L*er*) and the segregating F_2_ seedlings were screened using infrared thermography. A total of 868 *cis1* mutants were used for mapping. Twenty‐two simple sequence length polymorphism (SSLP) markers were used for bulked segregant analysis as described previously (Lukowitz *et al*., 2000). The Arabidopsis single nucleotide polymorphism (SNP) collections (http://www.arabidopsis.org/) were used to design SSLP, cleaved amplified polymorphic sequences (CAPS) and derived CAPS (dCAPS) markers for fine mapping. The mutation was narrowed down to a *c*. 100‐kb region at the top arm of chromosome III between SSLP marker nga172 and CAPS marker CA1, and is adjacent to SSLP marker nga32. T‐DNA insertion lines representing all the annotated genes within this region were obtained from NASC and screened using infrared thermal imaging. A T‐DNA insertion line (SALK_105495) of *At3g02260* which also showed morphological similarity to the mutant ‘*cis1*’ was identified. We performed an allelism test using the F_1_ progeny of the *cis1* and *big‐1* (SALK_105495) cross using thermal imaging. This confirmed that *cis1* and *big‐1* are allelic to each other. We used PCR‐based genotyping and gene sequencing to confirm the presence of a T‐DNA insertion in gene *At3g02260* of the SALK_105495 line and a single point mutation in gene *At3g02260* of the *cis1* mutant.

### Measurements of stomatal density, index, aperture and cell viability

Stomatal density and index were measured on leaf abaxial surfaces as described previously (Chater *et al*., 2015). The effect of CO_2_ on stomatal aperture was measured using the isolated epidermal strip bioassay technique as described previously (Chater *et al*., 2015). Forty stomatal pores were measured per treatment in three separate replicated tests. To avoid experimenter bias, all the aperture measurements were performed blind. Cell viability was assessed as described in Chater *et al*. (2015). Experiments on independently grown plant material were carried out three times and data were analysed by SigmaPlot 10 (Systat Software Inc., San Jose, CA, USA).

### Gas exchange measurements

Time‐resolved stomatal conductance analyses of intact leaves of 5‐wk‐old plants were conducted using a Li‐6400 gas exchange analyzer with a fluorometer chamber (Li‐Cor, Lincoln, NE, USA), as described by Hu *et al*. (2010). The photon flux density was set at 150 μmol m^−2^ s^−1^; temperature and relative humidity were held at 21°C and *c*. 60–70%, respectively. Stomatal conductance was stabilized at 400 ppm CO_2_ (as ambient concentration) for 30 min and then shifted to 800 ppm for another 30 min before being shifted to 100 ppm for 1.5 h. Data shown are the means ± SE, *n *=* *4 leaves for each genotype.

### Patch clamp experiments

Arabidopsis guard cell protoplasts were isolated according to the procedure described previously (Siegel *et al*., 2009). The whole‐cell currents were recorded using a patch clamp amplifier (Axopatch 200B) and a digitizer (Digidata 1550) (Molecular Devices LLC, Sunnyvale, CA, USA). CO_2_/bicarbonate‐activated S‐type anion currents were recorded as described previously (Xue *et al*., 2011). The bath solution contained 30 mM CsCl, 2 mM MgCl_2_, 1 mM CaCl_2_ and 10 mM Mes/Tris, pH 5.6. The pipette solution contained 150 mM CsCl, 2 mM MgCl_2_, 6.7 mM ethyleneglycol‐bis(β‐aminoethylether)‐*N*,*N*′‐tetraacetic acid (EGTA), 6.03 mM CaCl_2_ (2 μM free Ca^2+^), 5 mM Mg‐ATP, 10 mM HEPES/Tris, pH 7.1. Bicarbonate was freshly added to the pipette solution before patching the protoplasts each day. At pH 7.1, 11.5 mM free bicarbonate was balanced with 2 mM free CO_2_ in the pipette solution. For more details, please consult Xue *et al*. (2011).

### Reverse transcription‐polymerase chain reaction (RT‐PCR) and quantitative RT‐PCR analysis

Total RNA from aerial parts of the plants was prepared using an RNeasy total RNA mini kit (Qiagen, Hilden, Germany) and digested with RNase‐free DNase I (Thermo Fisher Scientific Inc. Waltham, MA, USA); the absence of genomic DNA contamination was confirmed by PCR using RNA as template without reverse transcription. First‐strand cDNA was synthesized using Superscript II^®^ reverse transcriptase (Invitrogen, Thermo Fisher Scientific) and oligo d(T)_15–18_ (Promega (Beijing) Biotech Co. Ltd, Beijing, China) mRNA primer with 1 μg of total RNA as the template. cDNA corresponding to 20 ng of total RNA and 300 nM of each primer were used in PCRs. The primers for RT‐PCR amplification of *BIG* fragments were: primer pair1, F_1_ (5′‐CAGCAAGCTCTATACCTTCAG‐3′) and R1 (5′‐TCCATCC ATCCACTCAACTC‐3′); primer pair 2, F_2_ (5′‐GTCTTCT ACTTCACTGACCAACTCC‐3′) and R2 (5′‐TCCATCTTC TTCTTCCTCTACATCC‐3′); Actin7 was amplified with forward primer (5′‐TGTTCCCAAGTATTGTTGGTCGTC‐3′) and reverse primer (5′‐TGCTGAGGGATGCAAGGA TTGATC‐3′) as a loading control. The PCR conditions were as follows: one cycle (94°C, 5 min), 35 cycles (94°C, 30 s; 62°C, 30 s; 72°C, 1 min), one cycle (72°C, 7 min). Quantitative PCR was carried out on an Mx3005P (Stratagene, La Jolla, CA, USA) or an ECO (Illumina Inc., San Diego, CA, USA) real‐time PCR thermal cycler in a total reaction volume of 20 μl using the SYBR green dye PCR Master Mix (Thermo Fisher Scientific) and the conditions 95°C for 10 min, 40 two‐step cycles at 95°C for 15 s and 60°C for 1 min, followed by dissociation melting curve analysis to determine the PCR specificity. The gene‐specific primers used for *BIG* were: F, 5′‐GAATGGGAAGGAGCTATGTTG‐3′; R, 5′‐GATACTGTG CTAAGGGAACTG‐3′; for *Actin3* (*At3g53750*), the primers were: F, 5′‐GGCAGAATATGATGAGTCAGG‐3′; R, 5′‐AAAGAAGAGCAGAGAACGAAG‐3′. The relative RNA levels were calculated from cycle threshold (*C*_T_) values according to the Δ*C*_T_ method, and relative target mRNA levels were normalized to *Actin3* mRNA levels. Reactions were repeated independently three times with similar results.

## Results

### The *cis1* mutant is involved in the response of stomatal conductance to elevated CO_2_

**Figure 1:**
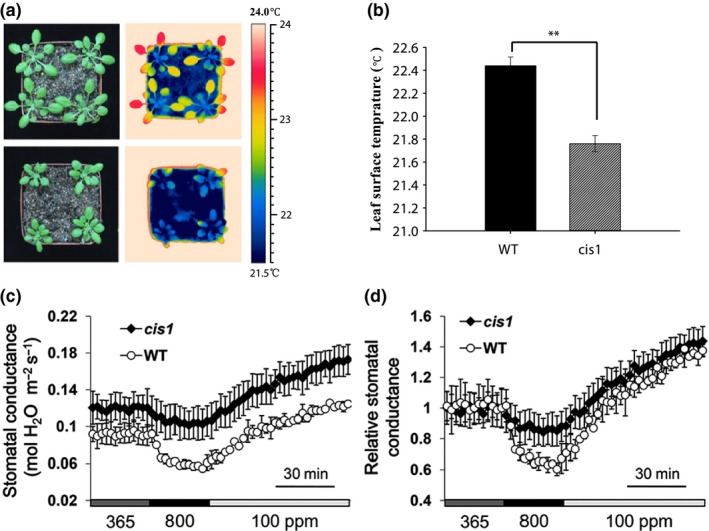
The *CO*_*2*_ *insensitive 1* (*cis1*) mutant displays a lower leaf surface temperature under elevated CO_2_ than wild‐type (WT) Arabidopsis. (a) Infrared thermograms showing that the leaf surface temperature of the *cis1* mutant is lower than that of WT when the plants are exposed to 1500 ppm CO_2_. (b) The average leaf surface temperature of the *cis1* mutant is *c*. 0.68°C lower than that of the WT plant when both are exposed to 1500 ppm CO_2_. Bars are mean ± SE (Student's *t‐*test, **, *P *≤* *0.001, *n *=* *20). (c) In contrast with WT, the *cis1* mutant fails to display elevated (800 ppm) CO_2_‐induced reduction in stomatal conductance, but exhibits a WT response when exposed to low (100 ppm) CO_2_. Bars are mean ± SE (representative data, *n *=* *4). (d) Relative stomatal conductance in (c). Bars are mean ± SE (representative data are presented, *n *=* *4).

To understand the underlying cellular basis of the effect of elevated CO_2_ on stomatal development and function, we carried out a forward genetic screen using infrared thermography. We reasoned that mutants failing to exhibit reductions in aperture, in this case induced by exposure to elevated [CO_2_], would be visible because they would exhibit reduced leaf temperature as a result of increased leaf evapotranspiration relative to WT (Darwin, 1904). Infrared thermography has been used previously to isolate mutants carrying lesions in stomatal responses to ABA (Raskin & Ladyman, 1988; Merlot *et al*., 2002), reduced atmospheric relative humidity (Xie *et al*., 2006; Liang *et al*., 2010) and CO_2_ (Hashimoto *et al*., 2006; Negi *et al*., 2008). Using this approach, we screened M2 plants from an EMS‐mutagenized population of Arabidopsis and identified *cis1* that displayed significantly lower leaf surface temperature (0.68°C) relative to WT when challenged for 40 min with 1500 ppm [CO_2_] (Fig. 1a,b). Genetic analysis revealed that this phenotype was caused by a single recessive Mendelian mutation (data not shown). To investigate the lesion in the *cis1* mutant further, we measured the stomatal conductance (*g*_s_). Figure 1(c,d) shows that, in WT, challenge with 800 ppm CO_2_ results in a reduction in *g*_s_, whereas the response is attenuated in *cis1*. By contrast, both *cis1* and WT display an increase in *g*_s_ when exposed to low (100 ppm) CO_2_. We confirmed this response in *big‐1*, a second independent allele of *cis1* (Supporting Information Fig. S1). These data suggest that the *cis1* mutant is compromised in the stomatal response to elevated [CO_2_].

### Identification of the *CIS1* gene locus

**Figure 2:**
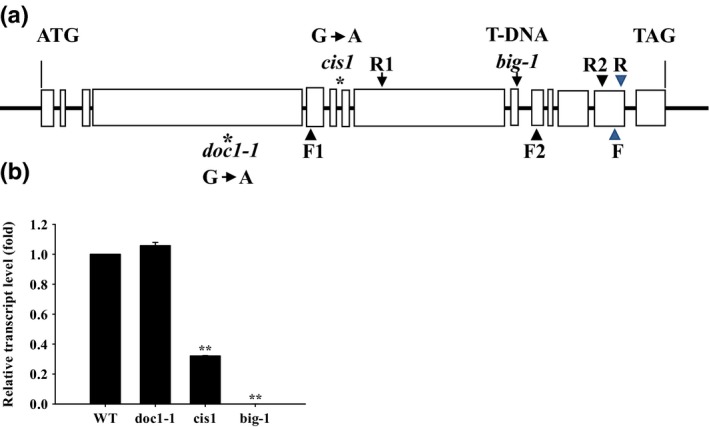
*CO*_*2*_ *insensitive 1* (*cis1*) is a new allele of the *BIG* gene in Arabidopsis. (a) Schematic structure of the *BIG* gene. The intron and exon organization of the *BIG* gene shown was determined by comparison of the cDNAs obtained by reverse transcription‐polymerase chain reaction (RT‐PCR) and genomic sequences from the Arabidopsis wild‐type (WT) Col‐0. Boxes indicate exons and lines between boxes indicate introns. The locations of the single base mutations and T‐DNA insertion of *cis1*,* doc1‐1* and *big‐1* are indicated. Diagram not to scale. * Indicates the site of point mutation. (b) The relative mRNA levels of *BIG* mutant alleles obtained by quantitative PCR with a pair of primers (F and R) with binding sites shown in (a). Values are mean ± SE,* n *=* *3.** Indicates the difference is statistically significant , *P* ≤ 0.001.

We performed map‐based gene cloning to identify the *CIS1* locus, and mapped the mutation to a 107‐kb region of chromosome III close to the *doc1* mutations (data not shown; Gil *et al*., 2001). Seeds for T‐DNA insertion lines of all annotated genes within this region were obtained from NASC and screened using infrared thermal imaging. A T‐DNA insertion line (SALK_105495) of *At3g02260* was identified that displayed similar thermal behavior to the *cis1* mutant. Sequencing of *cis1* revealed a single point mutation (G to A substitution) in locus *At3g02260* localized at a splicing acceptor site at position +8542 (GT…AG to GT…AA) (Fig. 2a), which resulted in alternative spliced mRNAs as shown in Fig. S2. Real‐time quantitative PCR revealed that, compared with WT, *cis1* (*At3g02260*) gene transcript abundance was reduced to a third (Fig. 2b).

*At3g02260* has previously been named *BIG* and is annotated as encoding a large protein of 5098 amino acids, containing multiple conserved functional domains including three putative Zn‐finger domains (Kanyuka *et al*., 2003; Kasajima *et al*., 2007). Our sequencing revealed that the original annotation is incorrect, as the open reading frame of *BIG* is 63 bp shorter than predicted, because 30 bp of the sequence of intron 1, 21 bp of intron 5 and 12 bp of intron 7 had been annotated as part of the respective neighboring exons (Notes S1). Hence, the *BIG* open reading frame (ORF) is 15 234 bp long, encoding a putative 5077‐amino‐acid peptide, as predicted by Gil *et al*. (2001).

Many alleles of *big* mutants, for example *ga6*,* tir3*,* doc1*,* asr1*,* lpr1*,* elk1*,* asa1*,* umb1*,* crm1* and *rao3*, have been independently isolated. All mutants are characterized by deficient organ elongation (dwarfism) and have altered root architecture, reduced apical dominance, defects in light responses and aberrant auxin transport. They also show altered sensitivities to GA, cytokinin, ethylene, low phosphate and water withholding treatments (Li *et al*., 1994; Ruegger *et al*., 1997; Sponsel *et al*., 1997; Gil *et al*., 2001; Lease *et al*., 2001; Kanyuka *et al*., 2003; López‐Bucio *et al*., 2005; Kasajima *et al*., 2007; Yamaguchi *et al*., 2007; Ivanova *et al*., 2014). Interestingly, insects and mammals possess homologs of the BIG protein and these are involved in signaling. Calossin/Pushover in *Drosophila melanogaster* and mammalian p600/UBR4 are homologs of BIG, both of which have a calmodulin (CaM) ‐binding domain and are probably involved in Ca^2+^ signaling (Xu *et al*., 1998; Parsons *et al*., 2015).

To confirm the identity of *cis1*, we obtained two additional mutant alleles of *BIG*. *doc1‐1* was originally isolated in a genetic screen for components of light signaling and harbors a single base change from G to A at position +5514 (Fig. 2a), resulting in a change from a conserved cysteine (Cys) residue to tyrosine (Tyr). This missense *BIG* mutation perturbs auxin transport and plant growth (Gil *et al*., 2001) but, in our quantitative PCR analysis, no change to the transcript abundance of *BIG* was detected (Fig. 2b). *big‐1* harbors a T‐DNA insertion in exon 9 before position +13 617 of the *BIG* gene (Kasajima *et al*., 2007) (Fig. 2a). We detected no *BIG* transcript in this mutant by quantitative PCR (Fig. 2b).

### *BIG* is also involved in the control of stomatal development by elevated CO_2_

**Figure 3:**
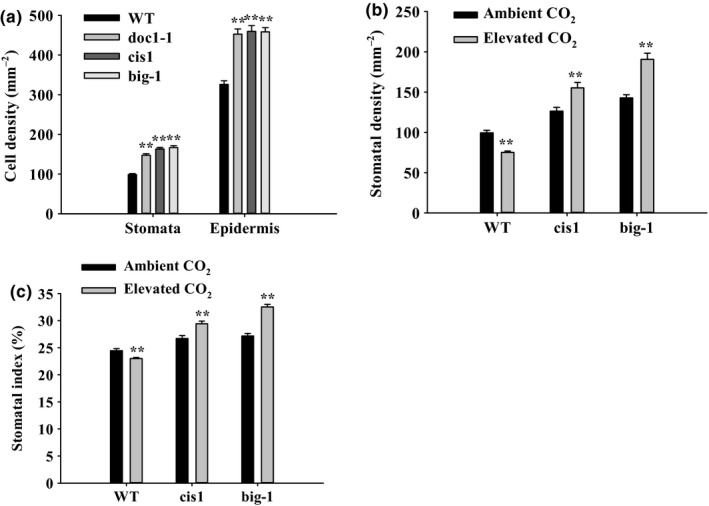
*BIG* gene mutants have higher stomatal density than wild‐type (WT) Arabidopsis. (a) Compared with WT,*BIG* mutants exhibit increased stomata and epidermal pavement cells (‘Epidermis’) density when grown at ambient [CO_2_]. Error bars represent ± SE (Mann–Whitney rank sum test, **, *P *≤* *0.001, *n *=* *72). (b) Stomatal density of WT and *BIG* mutant seedlings grown at ambient (450 ppm) and elevated (1000 ppm) [CO_2_]. When grown at 1000 ppm [CO_2_], the mean stomatal density of WT was significantly reduced compared with growth at ambient [CO_2_] (error bars represent ± SE (Mann–Whitney rank sum test, **, *P *≤* *0.001, *n *>* *20)), whereas, in the *BIG* gene alleles, stomatal density increased in these conditions (error bars represent ± SE (Student's *t‐*test, **, *P *≤* *0.001, *n *>* *20)). (c) Stomatal index of WT and *BIG* mutant seedlings grown at 450 ppm and 1000 ppm [CO_2_]. When grown at 1000 ppm, the mean stomatal index of WT was significantly reduced compared with growth at ambient [CO_2_] (error bars represent ± SE (Student's *t‐*test, **, *P *≤* *0.001, *n *>* *20)), whereas, in the *BIG* gene mutants, the stomatal index increased in these conditions (error bars represent ± SE (Student's *t‐*test, or Mann–Whitney rank sum test, **, *P *≤* *0.001, *n *>* *20)).

The data in Fig. 3(a) show that stomatal and epidermal pavement cell densities are greater in the *BIG* mutant alleles than in WT (*P *≤* *0.001). This reflects the fact that both guard cells and epidermal cells were significantly smaller than in WT (data not shown). Stomatal development is controlled by CO_2_, with stomatal density and index typically reduced in plants grown under elevated [CO_2_] (Woodward, 1987; Woodward & Kelly, 1995). We next investigated whether *BIG* has a role to play in the control of stomatal development by elevated [CO_2_]. In WT, growth at elevated [CO_2_] resulted in a decrease in stomatal density and index (Fig. 3b,c). In marked contrast, under the same conditions, growth at elevated [CO_2_] resulted in significant increases in both stomatal density and index in the *BIG* mutants (Fig. 3b,c). These data suggest that, in addition to controlling stomatal aperture, *BIG* is also required for the reduction in stomatal density and index caused by higher than ambient [CO_2_].

The *BIG* protein is involved in the signaling pathway by which elevated [CO_2_] induces stomatal closure, but not in the pathway through which elevated [CO_2_] inhibits stomatal opening.

**Figure 4:**
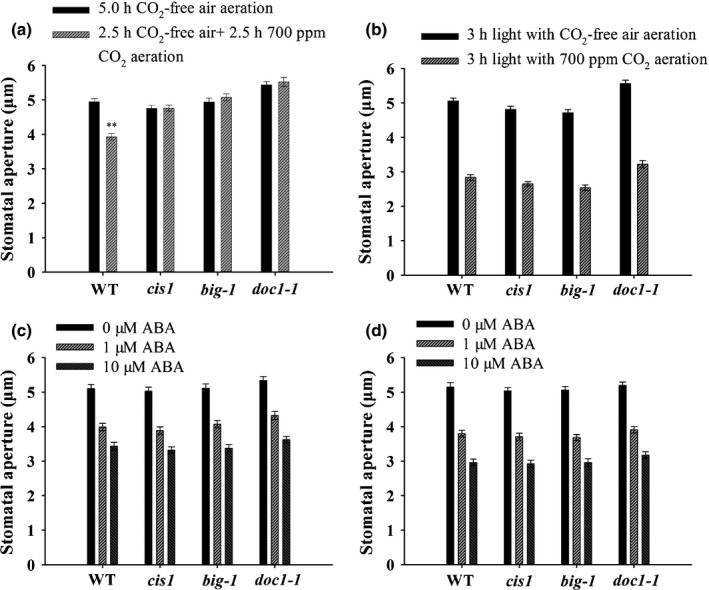
Stomatal responses of *BIG* gene mutants to elevated CO_2_ or exogenous abscisic acid (ABA) in Arabidopsis. (a) Elevated CO_2_‐induced stomatal closure is impaired in *BIG* gene mutants. Values are mean ± SE (Mann–Whitney rank sum test, **, *P *≤* *0.001, *n *=* *40). (b) Elevated CO_2_‐induced inhibition of stomatal opening is not compromised in *BIG* gene mutants. Error bars represent SE (*n *=* *40). (c) ABA‐induced stomatal closure is not compromised in *BIG* gene mutants. Bars are mean ± SE (*n *=* *40). (d) The inhibition of light‐induced stomatal opening by ABA is not compromised in *BIG* gene mutants. Values are mean ± SE (*n *=* *40). WT, wild‐type.

The results from the gas exchange experiments (Fig. 1c,d) prompted us to make direct measurements of stomatal responsiveness by quantifying changes in stomatal aperture (Chater *et al*., 2015). Figure 4(a) shows that, in contrast with WT, the stomata of *cis1*,* big‐1* and *doc1‐1* mutants failed to close when subjected to 700 ppm CO_2_. These data indicate that BIG is required for elevated CO_2_‐induced stomatal closure. Elevated [CO_2_] is also known to inhibit light‐induced stomatal opening (Mansfield *et al*., 1990). In contrast with CO_2_‐induced stomatal closure, the inhibition of light‐induced stomatal opening of the BIG mutants was similar to that of WT (Fig. 4b). The specific role of the *BIG* gene in the pathway by which elevated [CO_2_] brings about stomatal closure is highlighted by our observation that the series of allelic mutants all display WT behavior in response to ABA. This holds for both ABA‐induced stomatal closure and the inhibition by ABA of light‐induced stomatal opening (Fig. 4c,d). The intact stomatal ABA response as well as the impaired CO_2_ response were both observed in more than one of our laboratories, underlining the robustness of the CO_2_ specificity of the stomatal phenotype in *big* mutant alleles.

### BIG is required for the activation of S‐type anion channels by elevated bicarbonates

**Figure 5:**
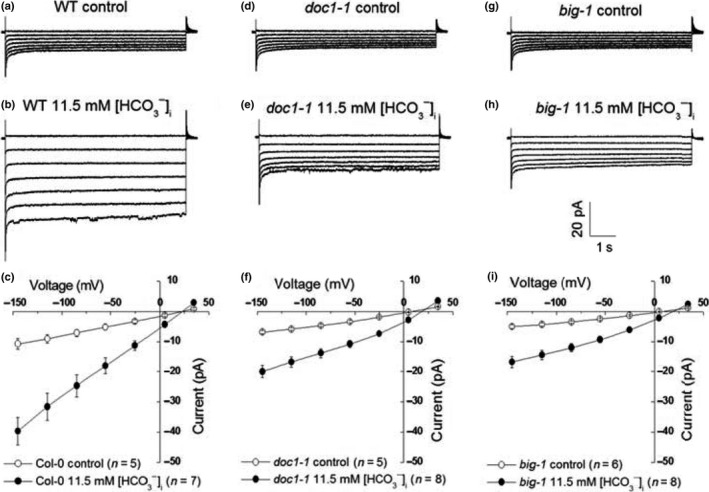
Bicarbonate‐activated S‐type anion currents were suppressed in *BIG* mutant guard cell protoplasts. (a) Typical recording in wild‐type (WT) guard cell protoplasts without bicarbonate. (b) Typical recording of 11.5 mM [HCO_3_^−^]_i_‐activated S‐type anion currents in WT guard cell protoplasts. (c) Average current–voltage relationships of whole‐cell currents as recording in (a) (open circles, *n *=* *5) and (b) (closed circles, *n *=* *7). Error bars represent ± SE. (d) Representative recording in *doc1‐1* mutant guard cell protoplasts without bicarbonate added to the pipette solution. (e) Representative whole‐cell current recording in *doc1‐1* mutant guard cell protoplasts with 11.5 mM [HCO_3_^−^]_i_ added to the pipette solution. (f) Average current–voltage relationships of whole‐cell currents as recording in (d) (open circles, *n *=* *5) and (e) (closed circles, *n *=* *8). Error bars represent ± SE. (g) Representative recording in *big‐1* mutant guard cell protoplasts without bicarbonate added to the pipette solution. (h) Representative whole‐cell current recording in *big‐1* mutant guard cell protoplasts with 11.5 mM [HCO_3_^−^]_i_ bicarbonate added to the pipette solution. (i) Average current–voltage relationships of whole‐cell currents as recording in (g) (open circles, *n *=* *6) and (h) (closed circles, *n *=* *8). Error bars represent ± SE.

S‐type anion channels are recognized as one of the main players in guard cell signaling. They mediate the release of anions from guard cells and promote stomatal closure in response to diverse stimuli, including increased [CO_2_] (Kollist *et al*., 2011; Wang *et al*., 2016). An increase in the cytoplasmic bicarbonate concentration activates S‐type anion channels in guard cells and correlates with elevated [CO_2_]‐induced stomatal closure in diverse mutant backgrounds (Vahisalu *et al*., 2008; Xue *et al*., 2011; Merilo *et al*., 2013). To understand the role of BIG in guard cell signaling further, we investigated whether the activation of S‐type anion channels by applied bicarbonate was impaired by mutations in *BIG*. In WT guard cell protoplasts, large anion currents were recorded when the pipette solution contained 11.5 mM free bicarbonate (Fig. 5b). However, in guard cell protoplasts of the *doc1‐1* and *big‐1* mutant alleles, lower anion currents were activated by the same concentration of bicarbonate in the pipette solution (Fig. 5e,h). At a voltage of −145 mV, the average activated currents were −39.7 ± 4.6 pA for WT (Fig. 5c), −20.0 ± 2.0 pA for the *doc1‐1* mutant (Fig. 5f) and −16.8 ± 1.8 pA for the *big‐1* mutant (Fig. 5i). The differences between WT and each mutant allele of *BIG* were statistically significant (*P *≤* *0.01). These results demonstrate that the BIG protein is required for elevated intracellular bicarbonate‐induced activation of guard cell plasma membrane S‐type anion channel currents that function in CO_2_‐induced stomatal closure and further reinforce the importance of BIG in stomatal closure.

## Discussion

### BIG is involved in stomatal closure induced by elevated CO_2_, but not in elevated CO_2_‐induced inhibition of stomatal opening

We conducted a genetic screen that resulted in the identification of a novel allele of the *BIG* gene that we call *CIS1*, which plays a regulatory role in stomatal function and development. Our phenotypic analyses revealed that *CIS1* is involved in the reduction in stomatal conductance induced by elevated CO_2_ (Figs 1b,c, S1). On the surface of a leaf, during the day, stomata are exposed to frequently conflicting signals from the environment. Guard cells integrate these signals and the overall result is the optimization of gas exchange under the prevailing environmental conditions. Looking at this more closely, in the case of stomatal closure, it is necessary to stimulate the processes associated with the loss of guard cell turgor, whilst simultaneously inhibiting the cellular reactions involved in solute accumulation and stomatal opening. The opening and closure responses are physiologically distinct and are not the reverse of each other (Assmann, 1993; Li *et al*., 2000). When we investigated the role of *BIG* in these processes, we found, intriguingly, that it was only involved in elevated CO_2_‐induced stomatal closure. In marked contrast, all of the BIG mutants exhibited WT behavior in our bioassay of CO_2_ inhibition of light‐stimulated stomatal opening (Fig. 4a,b). To extend our investigation of the role of BIG in the regulation of stomatal aperture, we also investigated whether it played a role in stomatal closure induced by ABA. The data in Fig. 4(c,d) clearly indicate that BIG is not involved in ABA‐promoted closure or in ABA‐inhibited light‐induced opening. Because *BIG* encodes a protein which, in guard cells, is only involved in CO_2_‐induced closure and not CO_2_‐inhibited opening, this makes it possible, at the molecular level, to distinguish, and to start to define, these different processes. In this sense, these data fit well with the observation that, in molecular terms, ABA‐induced stomatal closure is distinct from the inhibition of opening by ABA. Examples include GPA1, which is involved in ABA inhibition of opening, but not in closure (Wang *et al*., 2001), a sphingosine‐1‐phosphate phosphatase, long‐chain base phosphate lyase double mutant (*sppasedpl1*), which displays WT behavior during ABA‐induced closure, but is slightly impaired in the ABA inhibition of stomatal opening response (Worrall *et al*., 2008), PI‐phospholipase C, which is involved in the ABA‐inhibition of opening, but not closure (Mills *et al*., 2004), and the observation that some members of the PYR/PYL ABA receptor family involved in stomatal opening inhibition are different from those involved in stomatal closure induction (Yin *et al*., 2013). The second striking result to emerge from these experiments is that BIG is not involved in ABA‐induced reductions in stomatal aperture (Fig. 4c,d). This suggests that the BIG protein lies upstream of the point of convergence of the guard cell CO_2_ and ABA signaling pathways (Webb & Hetherington, 1997; Xue *et al*., 2011; Merilo *et al*., 2013; Chater *et al*., 2015; Jakobson *et al*., 2016; Yamamoto *et al*., 2016). Looking downstream of the point of convergence, it is well known that both ABA‐ and CO_2_‐induced stomatal closure involve the activation of slow anion channels (Kim *et al*., 2010; Assmann & Jegla, 2016; Engineer *et al*., 2016). Our data reveal that mutations in BIG depressed the activation of S‐type anion channels by bicarbonate (Fig. 5), in line with the impaired elevated [CO_2_]‐induced stomatal closure. A recent study by Yamamoto *et al*. (2016) has provided evidence that different parts of SLAC1 are separately responsible for sensing ABA and CO_2_ signals. It is the transmembrane domain of SLAC1 channels that perceives CO_2_ signals, in contrast with the N‐ and C‐terminal ends of SLAC1 which are responsible for ABA signaling in Arabidopsis (Brandt *et al*., 2015; Yamamoto *et al*., 2016). Further investigation is needed to determine whether the activation of S‐type anion channels by ABA is affected by the loss of *BIG* gene function.

### BIG is also involved in the control of stomatal development by elevated CO_2_

Figure 3(a) shows that mutations in BIG result in significant increases in guard and epidermal pavement cell densities, consistent with the findings of Guo *et al*. (2013). Growth at elevated [CO_2_] typically results in a reduction in stomatal index and density (Hetherington & Woodward, 2003; Assmann & Jegla, 2016; Engineer *et al*., 2016). The results in Fig. 3(b,c) clearly show that, in marked contrast with WT, the stomatal indices and density of BIG mutants increased when the plants were grown at 1000 ppm CO_2_. The epidermal cell densities in the mutants remained significantly higher than those of WT at this elevated [CO_2_] (Fig. S3). It is likely, as with *ßca1ca4*,* epf2* and *hic* mutants (Gray *et al*., 2000; Engineer *et al*., 2014), that loss of BIG function relieves the elevated [CO_2_]‐mediated repression of stomatal development. How might BIG bring about an effect on CO_2_‐mediated stomatal development? One possibility that would merit future investigation is that this is an auxin‐related response. The *BIG* gene has been reported to encode a protein associated with auxin transport (Gil *et al*., 2001; Kanyuka *et al*., 2003) and is specifically required in the process by which auxin inhibits endocytosis and promotes its own efflux from cells (Paciorek *et al*., 2005). In this context, it is worth noting that evidence is emerging that auxin inhibits stomatal development. Mutants disrupted in the TAA1/TAR auxin biosynthesis or polar auxin transport and auxin signaling, as observed in multiple *tir1/afb* auxin receptor mutants, cause stomatal clustering (Balcerowicz *et al*., 2014; Le *et al*., 2014; Zhang *et al*., 2014). However, we observed no stomatal clustering in the *cis1* and related mutants. Further work will be required to reveal whether disruptions to auxin signaling underlie the BIG stomatal mutant phenotype.

In conclusion, we demonstrate that, in Arabidopsis*,* the BIG protein is involved in the elevated [CO_2_]‐mediated control of stomatal closure and density. Our results reveal that we have identified a component which is involved in the signaling pathway by which elevated CO_2_ promotes stomatal closure. However, BIG is not involved in the elevated [CO_2_]‐mediated inhibition of light‐induced opening or in stomatal closure initiated by ABA. These data indicate that elevated [CO_2_]‐mediated closure and inhibition of opening are, in molecular terms, distinguishable. Our data suggest that BIG lies upstream of the point of convergence of ABA and CO_2_, or resides in an as yet undefined parallel signaling pathway that converges at or above the SLAC1 ion channel.

## Author contributions

A.M.H. conceived the study. Y‐K.L. and A.M.H. designed the research. Y‐K.L., J.H., R‐X.Z., K.P., C.T., S.L., S.X., A.L., H.H., J.Z., K.E.H. and K.H. conducted the experiments. J.H., J.K., M.R.M., J.E.G., J.I.S., Y‐K.L. and A.M.H. analyzed the data. A.M.H., Y‐K.L. and J.E.G. wrote the manuscript. All authors read and approved the manuscript.

## Supporting information

Please note: Wiley Blackwell are not responsible for the content or functionality of any Supporting Information supplied by the authors. Any queries (other than missing material) should be directed to the *New Phytologist* Central Office.

**Fig. S1** The *big‐1* mutant fails to display elevated (800 ppm) CO_2_‐induced reduction in stomatal conductance.
**Fig. S2** PCR amplification of the *BIG* fragment from cDNAs of wild‐type (WT) and mutant plants.
**Fig. S3** Epidermal cell density of wild‐type (WT) and *BIG* gene mutant seedlings grown at elevated (1000 ppm) [CO_2_].

**Notes S1** Determination of the intron–exon structure of *BIG* by DNA sequencing.
