# Supplementary material for: The BIG protein distinguishes the process of CO 2‐induced stomatal closure from the inhibition of stomatal opening by CO 2
Source: New Phytol. 2018 Jan 2;218(1):232–41. doi: 10.1111/nph.14957 (PMC5887946; doi:10.1111/nph.14957)
Supplement: Supplementary file 1 — Fig. S1 The big‐1 mutant fails to display elevated (800 ppm) CO2‐induced reduction in stomatal conductance. Fig. S2 PCR amplification of the BIG fragment from cDNAs of wild‐type (WT) and mutant plants. Fig. S3 Epidermal cell density of wild‐type (WT) and BIG gene mutant seedlings grown at elevated (1000 ppm) [CO2]. [file NPH-218-232-s001.pdf]

New Phytologist **Supporting Information**

Article title: **The BIG protein distinguishes the process of CO_2-induced stomatal closure from the inhibition of stomatal opening by CO_2.**

Authors: Jingjing He[1,#], Ruo-Xi Zhang[1,#], Kai Peng[2], Cecilia Tagliavia[3],Siwen Li[1],Shaowu Xue[4], Amy Liu[5], Honghong Hu[4,5], Jingbo Zhang[5], Katherine E Hubbard[5,6], Katrin Held[7], Martin R McAinsh[3], Julie E Gray[8], Jörg Kudla[7], Julian I Schroeder[5], Yun-Kuan Liang[1]* and Alistair M Hetherington[2]*

Article acceptance date: 12 November 2017

The following Supporting Information is available for this article:

Fig. S1 The*big-1* mutant fails to display elevated (800 ppm) CO_2-induced reduction in stomatal conductance.

Fig. S2 PCR amplification of the *BIG* fragment from cDNAs of WT and mutant plants.

Fig. S3 Epidermal cell density of WT and *BIG* gene mutant seedlings grown at elevated 1,000 ppm [CO_2].

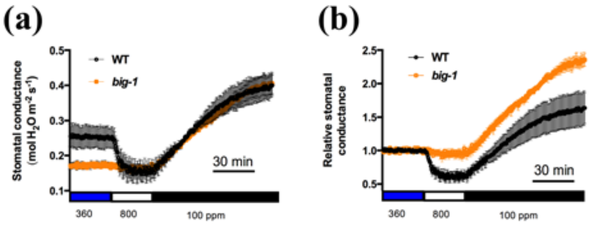

Fig. S1 (a) In contrast to WT, the *big-1*mutant fails to display elevated (800 ppm) CO_2-induced reduction in stomatal conductance, but exhibits a WT response when exposed to low (100 ppm) CO_2. Error bars represent ± SE (representative data, n=4). (b) Relative stomatal conductance in (a). Error bars represent ± SE (representative data, n=4).

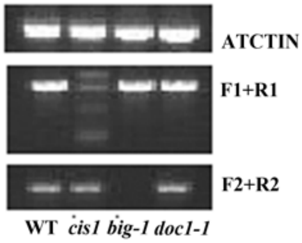

Fig. S2 PCR amplification of the *BIG* fragment from cDNAs of WT and mutant plants showed a complex PCR band pattern of *cis1* and only truncated *BIG* transcript present in *big-1* whereas no change in mRNA abundance is detected in the *doc1-1* mutant. Primer binding sites as indicated in Fig. 2a. *ACTIN* was usedas a reference gene.

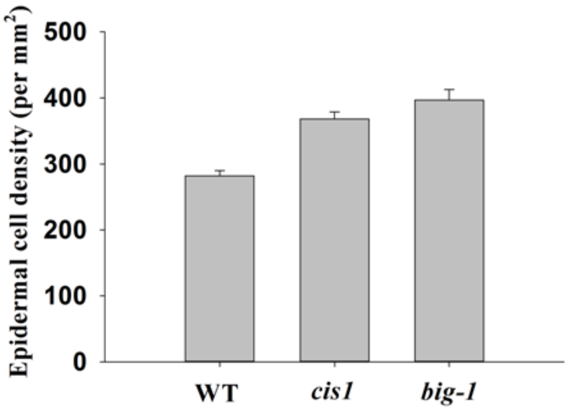

Fig. S3 Epidermal cell density of WT and *BIG*gene mutant seedlings grown at elevated 1,000 ppm [CO_2].
