# Supplementary material for: The BIG protein distinguishes the process of CO 2‐induced stomatal closure from the inhibition of stomatal opening by CO 2
Source: New Phytol. 2018 Jan 2;218(1):232–41. doi: 10.1111/nph.14957 (PMC5887946; doi:10.1111/nph.14957)
Supplement: Supplementary file 2 — Notes S1 Determination of the intron–exon structure of BIG by DNA sequencing. [file NPH-218-232-s002.pdf]

***New Phytologist* Supporting Information**

Article title: **The BIG protein distinguishes the process of CO_2-induced stomatal closure from the inhibition of stomatal opening by CO_2.**

Authors: Jingjing He[1,#], Ruo-Xi Zhang[1,#], Kai Peng[2], Cecilia Tagliavia[3], Siwen Li[1], Shaowu Xue[4], Amy Liu[5], Honghong Hu[4,5], Jingbo Zhang[5], Katherine E Hubbard[5,6], Katrin Held[7], Martin R McAinsh[3], Julie E Gray[8], Jörg Kudla[7], Julian I Schroeder[5], Yun-Kuan Liang[1]* and Alistair M Hetherington[2]*

Article acceptance date: 12 November 2017

The following Supporting Information is available for this article:

Notes S1: Determination of the intron-exon structure of *BIG* by DNA sequencing

1. Summary

We managed by using 21 sets of primer pair together with each empirically optimized PCR condition to amplify and sequence through the cDNA of *BIG* (*At3g02260*) gene. Our result revealed that the actual length of the ORF of BIG is 15,234bp, which is 63bp shorter than the one predicted by Kanyuka *et al.* (2003). It is because a 30bp (GTACTGACTTGACACTGAATCAAGGTTTAG) of the sequence of intron 1, a 21 bp (GTAATGTTTCTACCAAATTCA) of intron 5 and a 12bp (GTGTGTTCTTGT) of intron 7 were interpreted as part of their respective neighboring exons by TAIR. Therefore the ORF of *BIG* gene is 15,234bp long encoding a putative peptide of 5,077 amino acids same to the length predicted by Gil *et al.* (2001).

2. The intron-exon structure of BIG shown by the sequence alignment using CLUSTAL 2.1

BIG-GENOME is to refer to the genomic sequence of *BIG* gene downloaded from TAIR website;
BIG-ORF is the actual ORF of *BIG* determined by DNA sequencing;
BIG-TAIR is the ORF of *BIG* annotated by TAIR.

```
BIG-GENOME      ATGGCAGATGACTTGGCGAATCTCTGCCGATTTCTCTTCGACGACACCGCCTTTCCCTCT
BIG-ORF         ATGGCAGATGACTTGGCGAATCTCTGCCGATTTCTCTTCGACGACACCGCCTTTCCCTCT
BIG-TAIR        ATGGCAGATGACTTGGCGAATCTCTGCCGATTTCTCTTCGACGACACCGCCTTTCCCTCT
                ************************************************************
BIG-GENOME      TTGTCGTCTTCGGCATCTTCAGATCTCTTTTCTCGCCGTTTACGCTCCGATGATTCGATC
BIG-ORF         TTGTCGTCTTCGGCATCTTCAGATCTCTTTTCTCGCCGTTTACGCTCCGATGATTCGATC
BIG-TAIR        TTGTCGTCTTCGGCATCTTCAGATCTCTTTTCTCGCCGTTTACGCTCCGATGATTCGATC
                ************************************************************
BIG-GENOME      AAGCGCGGTCTCCGTTCTTTCTATCTCCTCCTTCGCTGGGGTGTCGCTCCGATCGGCGGA
BIG-ORF         AAGCGCGGTCTCCGTTCTTTCTATCTCCTCCTTCGCTGGGGTGTCGCTCCGATCGGCGGA
BIG-TAIR        AAGCGCGGTCTCCGTTCTTTCTATCTCCTCCTTCGCTGGGGTGTCGCTCCGATCGGCGGA
                ************************************************************
BIG-GENOME      GACGATGCTGATTCCTCTGGCAAGCTACGCTTTGAGACATGGTCCGATTCGCAGCTTCAA
BIG-ORF         GACGATGCTGATTCCTCTGGCAAGCTACGCTTTGAGACATGGTCCGATTCGCAGCTTCAA
BIG-TAIR        GACGATGCTGATTCCTCTGGCAAGCTACGCTTTGAGACATGGTCCGATTCGCAGCTTCAA
                ************************************************************
```

```
BIG-GENOME    GCTCTTGTTTCGATATCTCAGGCGATCCTTCTACTATCTCGATCTCTATTAGGTACTGAC
BIG-ORF       GCTCTTGTTTCGATATCTCAGGCGATCCTTCTACTATCTCGATCTCTATTAG--------
BIG-TAIR      GCTCTTGTTTCGATATCTCAGGCGATCCTTCTACTATCTCGATCTCTATTAGGTACTGAC
              ****************************************************
BIG-GENOME    TTGACACTGAATCAAGGTTTAGGTCGGTATCAATTTAGCTGTCCATTTCTAGTTCGTCTT
BIG-ORF       ------------------------------------------------------------
BIG-TAIR      TTGACACTGAATCAAGGTTTAG--------------------------------------

BIG-GENOME    CCACTTCTGAGCTTTGGCATCTGCTTTTTTTGTTGCTATCCTAGATGAGTTACTAGTTA
BIG-ORF       ------------------------------------------------------------
BIG-TAIR      ------------------------------------------------------------

BIG-GENOME    TTTTCCATCTGAACAGTGATGATCTCGAATCATTTTCCTTACTGTTTTTTTTTTTTTGGT
BIG-ORF       ------------------------------------------------------------
BIG-TAIR      ------------------------------------------------------------

BIG-GENOME    GTATCTTAGTGGACCAACTCGAACCAATCGTTTTGGGTGTCATCCAGGAGGTGATGGAAT
BIG-ORF       ---------TGGACCAACTCGAACCAATCGTTTTGGGTGTCATCCAGGAGGTGATGGAAT
BIG-TAIR      ---------TGGACCAACTCGAACCAATCGTTTTGGGTGTCATCCAGGAGGTGATGGAAT
                       ***************************************************
BIG-GENOME    TCTCTCTCAGCTTCTTGGAGAAGTCAAGTTTTAGACAAAATGATCTCAAAATGGAGGTTT
BIG-ORF       TCTCTCTCAGCTTCTTGGAGAAGTCAAGTTTTAGACAAAATGATCTCAAAATGGAG----
BIG-TAIR      TCTCTCTCAGCTTCTTGGAGAAGTCAAGTTTTAGACAAAATGATCTCAAAATGGAG----
              ********************************************************
BIG-GENOME    GTTCTTTATTATTAATTTGTGATAGAGATTCAGTAGGGAAGTCTTTAATGTTGGTCTTAT
BIG-ORF       ------------------------------------------------------------
BIG-TAIR      ------------------------------------------------------------

BIG-GENOME    TTTGGTACACTGGGTAAGTCTAGGTAATCTGCTTGAGGTCCTTTTTGAGCATAGACATAT
BIG-ORF       ------------------------------------------------------------
BIG-TAIR      ------------------------------------------------------------

BIG-GENOME    CTTCTAGAGAATTTGTTTTTATTTGTGTTTGTTTTTCGATGATTAGACGTTTGAACTCGT
BIG-ORF       ------------------------------------------------------------
BIG-TAIR      ------------------------------------------------------------

BIG-GENOME    TTTATAAGACTTTATCAGGTTTTTGACACTAGTGGTCAGGTTTTCATTGTTGCGCCATAG
BIG-ORF       ------------------------------------------------------------
BIG-TAIR      ------------------------------------------------------------

BIG-GENOME    AATTTTGTCACATATGCTAACCAGCATGTTTTCGTTTTACCTTTTCCATGAGGGTTATAG
BIG-ORF       ------------------------------------------------------------
BIG-TAIR      ------------------------------------------------------------
```

```
BIG-GENOME      TATGGTTACGGTCTACTTCCATTAGTAACTCTTTGTCTATCACCAAAACATTTGTGGACT
BIG-ORF         ------------------------------------------------------------
BIG-TAIR        ------------------------------------------------------------

BIG-GENOME      ACTGACTTGGTTTCTTACTCTGTTTCTATCAAGTTTGACTGCATTAGTAATATCTCCAAA
BIG-ORF         ------------------------------------------------------------
BIG-TAIR        ------------------------------------------------------------

BIG-GENOME      TGCTTGCTTGAATCAGATCAACATGGAAATACTTTTGGAAATTGCTTCTTTTGATGGAAG
BIG-ORF         ----------------ATCAACATGGAAATACTTTTGGAAATTGCTTCTTTTGATGGAAG
BIG-TAIR        ----------------ATCAACATGGAAATACTTTTGGAAATTGCTTCTTTTGATGGAAG
                                ********************************************
BIG-GENOME      TGAGAAGCAATATGATATATTACCAGATTTCTCTCCCGCTGAAGTTGCAGAACTGTGGCC
BIG-ORF         TGAGAAGCAATATGATATATTACCAGATTTCTCTCCCGCTGAAGTTGCAGAACTGTGGCC
BIG-TAIR        TGAGAAGCAATATGATATATTACCAGATTTCTCTCCCGCTGAAGTTGCAGAACTGTGGCC
                ************************************************************
BIG-GENOME      AGCTTTTTCTGGCGAGCATGATAATATGGACGCACAGAGCCTTGTGAAATGTACTTTTCA
BIG-ORF         AGCTTTTTCTGGCGAGCATGATAATATGGACGCACAGAGCCTTGTGAAATGTACTTTTCA
BIG-TAIR        AGCTTTTTCTGGCGAGCATGATAATATGGACGCACAGAGCCTTGTGAAATGTACTTTTCA
                ************************************************************
BIG-GENOME      AGGTATTGTTTCTACCCATCCACTCAGCAATATGTTTTCTTACTTCAAATTTATTTGATG
BIG-ORF         AG----------------------------------------------------------
BIG-TAIR        AG----------------------------------------------------------
                **
BIG-GENOME      ACCCTTACATCTTTTGACATCTCAAGCAGGGGGCAGATGCTCAAATGAAGAGAAGCCAGT
BIG-ORF         -----------------------------GGGGCAGATGCTCAAATGAAGAGAAGCCAGT
BIG-TAIR        -----------------------------GGGGCAGATGCTCAAATGAAGAGAAGCCAGT
                                             *******************************
BIG-GENOME      TGACCGGCTTCTTATTACACTGATGTCTGAATGCATTGAATCTGATGTCCAGGCGCAGTC
BIG-ORF         TGACCGGCTTCTTATTACACTGATGTCTGAATGCATTGAATCTGATGTCCAGGCGCAGTC
BIG-TAIR        TGACCGGCTTCTTATTACACTGATGTCTGAATGCATTGAATCTGATGTCCAGGCGCAGTC
                ************************************************************
BIG-GENOME      AGTAGTAAAGTCCCCTTTTCAACAAGACTGTGGAGACCTGAATCCCTTTACTCGACACCT
BIG-ORF         AGTAGTAAAGTCCCCTTTTCAACAAGACTGTGGAGACCTGAATCCCTTTACTCGACACCT
BIG-TAIR        AGTAGTAAAGTCCCCTTTTCAACAAGACTGTGGAGACCTGAATCCCTTTACTCGACACCT
                ************************************************************
BIG-GENOME      GGCAGTTGTTCACCTTCGCTGTGTTTGTCGCCTGATTATGGTCTGCAAAGAGCTTGTTCA
BIG-ORF         GGCAGTTGTTCACCTTCGCTGTGTTTGTCGCCTGATTATGGTCTGCAAAGAGCTTGTTCA
BIG-TAIR        GGCAGTTGTTCACCTTCGCTGTGTTTGTCGCCTGATTATGGTCTGCAAAGAGCTTGTTCA
                ************************************************************
BIG-GENOME      ATTGCCAAATATGTTGGATGAGAAGACGGTTGATCAAGCTGTCCTTGATAAATTGTCGTT
BIG-ORF         ATTGCCAAATATGTTGGATGAGAAGACGGTTGATCAAGCTGTCCTTGATAAATTGTCGTT
BIG-TAIR        ATTGCCAAATATGTTGGATGAGAAGACGGTTGATCAAGCTGTCCTTGATAAATTGTCGTT
                ************************************************************
```

```
BIG-GENOME      CTGCTTAAGAATTTTGAAGCTGCTTGGGAGTCTTTCAAAAGATGTTCAAAGTATAGAAAA
BIG-ORF         CTGCTTAAGAATTTTGAAGCTGCTTGGGAGTCTTTCAAAAGATGTTCAAAGTATAGAAAA
BIG-TAIR        CTGCTTAAGAATTTTGAAGCTGCTTGGGAGTCTTTCAAAAGATGTTCAAAGTATAGAAAA
                ************************************************************
BIG-GENOME      CGACGGCTCATTGTTACAAGCAGTAGCTTCATTTACAGATGCTTTTCCTAAGCTGTTTAG
BIG-ORF         CGACGGCTCATTGTTACAAGCAGTAGCTTCATTTACAGATGCTTTTCCTAAGCTGTTTAG
BIG-TAIR        CGACGGCTCATTGTTACAAGCAGTAGCTTCATTTACAGATGCTTTTCCTAAGCTGTTTAG
                ************************************************************
BIG-GENOME      GGTTTTCTTTGAATTCACTAACCACACTGCTACAGAGGGTAATATTGAGAGTCTTTCTCT
BIG-ORF         GGTTTTCTTTGAATTCACTAACCACACTGCTACAGAGGGTAATATTGAGAGTCTTTCTCT
BIG-TAIR        GGTTTTCTTTGAATTCACTAACCACACTGCTACAGAGGGTAATATTGAGAGTCTTTCTCT
                ************************************************************
BIG-GENOME      TGCGCTAGTGGAGGGATTTCTTAATCTCGTCCAACTCATTTTTGGCAAGAGCAGTGTTTT
BIG-ORF         TGCGCTAGTGGAGGGATTTCTTAATCTCGTCCAACTCATTTTTGGCAAGAGCAGTGTTTT
BIG-TAIR        TGCGCTAGTGGAGGGATTTCTTAATCTCGTCCAACTCATTTTTGGCAAGAGCAGTGTTTT
                ************************************************************
BIG-GENOME      TCAGAATGTCCAGGCATGTGTGGCAGCTTCTATTGTCAGCAATCTAGATTCCTCAGTGTG
BIG-ORF         TCAGAATGTCCAGGCATGTGTGGCAGCTTCTATTGTCAGCAATCTAGATTCCTCAGTGTG
BIG-TAIR        TCAGAATGTCCAGGCATGTGTGGCAGCTTCTATTGTCAGCAATCTAGATTCCTCAGTGTG
                ************************************************************
BIG-GENOME      GAGATATGATGGGTCCTCTTGTAATTTGACACCTCCACTTGCTTACTTCCCTCGGTCTGT
BIG-ORF         GAGATATGATGGGTCCTCTTGTAATTTGACACCTCCACTTGCTTACTTCCCTCGGTCTGT
BIG-TAIR        GAGATATGATGGGTCCTCTTGTAATTTGACACCTCCACTTGCTTACTTCCCTCGGTCTGT
                ************************************************************

BIG-GENOME      CATCTACACATTGAAACTCATTCAAGATCTCAAGAGACAACCATACCATATCCATGATCT
BIG-ORF         CATCTACACATTGAAACTCATTCAAGATCTCAAGAGACAACCATACCATATCCATGATCT
BIG-TAIR        CATCTACACATTGAAACTCATTCAAGATCTCAAGAGACAACCATACCATATCCATGATCT
                ************************************************************
BIG-GENOME      GAGGGTTTTAGAGTCTGAAGTTACCTATGAAGATGTCAGTTCCACTGTTGATTCTGTTTA
BIG-ORF         GAGGGTTTTAGAGTCTGAAGTTACCTATGAAGATGTCAGTTCCACTGTTGATTCTGTTTA
BIG-TAIR        GAGGGTTTTAGAGTCTGAAGTTACCTATGAAGATGTCAGTTCCACTGTTGATTCTGTTTA
                ************************************************************
BIG-GENOME      TTTCCATCTGCGCCAGGAAAAGATCCCGTTGCTCAAGTGCTTCACGGTCGAGGATATAAT
BIG-ORF         TTTCCATCTGCGCCAGGAAAAGATCCCGTTGCTCAAGTGCTTCACGGTCGAGGATATAAT
BIG-TAIR        TTTCCATCTGCGCCAGGAAAAGATCCCGTTGCTCAAGTGCTTCACGGTCGAGGATATAAT
                ************************************************************
BIG-GENOME      GAGAGTGATATTTCCTTCATCAAGTCAATGGATGGATAACTTCTTCCATCTTGTTTATTT
BIG-ORF         GAGAGTGATATTTCCTTCATCAAGTCAATGGATGGATAACTTCTTCCATCTTGTTTATTT
BIG-TAIR        GAGAGTGATATTTCCTTCATCAAGTCAATGGATGGATAACTTCTTCCATCTTGTTTATTT
                ************************************************************
BIG-GENOME      CCTCCATCGCGAAGGTGTAAAATTACGACCAAAAGTGGAGAGGACGTATTCCAGTTTAAG
BIG-ORF         CCTCCATCGCGAAGGTGTAAAATTACGACCAAAAGTGGAGAGGACGTATTCCAGTTTAAG
BIG-TAIR        CCTCCATCGCGAAGGTGTAAAATTACGACCAAAAGTGGAGAGGACGTATTCCAGTTTAAG
```

```
            ************************************************************
BIG-GENOME  ATCCAACAGTTTTGCAGAAGTTGAAAGCCAAATTTCTCATGACGATGAAGCCCTGTTTGG
BIG-ORF     ATCCAACAGTTTTGCAGAAGTTGAAAGCCAAATTTCTCATGACGATGAAGCCCTGTTTGG
BIG-TAIR    ATCCAACAGTTTTGCAGAAGTTGAAAGCCAAATTTCTCATGACGATGAAGCCCTGTTTGG
            ************************************************************
BIG-GENOME  AAACTTATTTTCTGAGGGTAGTCGTTCTCTCTGTTCCATAGAACCAAATGATCAACCACC
BIG-ORF     AAACTTATTTTCTGAGGGTAGTCGTTCTCTCTGTTCCATAGAACCAAATGATCAACCACC
BIG-TAIR    AAACTTATTTTCTGAGGGTAGTCGTTCTCTCTGTTCCATAGAACCAAATGATCAACCACC
            ************************************************************
BIG-GENOME  TGTTTCTGTCAGCAGCAATTTACTATTGCAAGCTGCTAAGGAACTGTTGAATTTTCTTAG
BIG-ORF     TGTTTCTGTCAGCAGCAATTTACTATTGCAAGCTGCTAAGGAACTGTTGAATTTTCTTAG
BIG-TAIR    TGTTTCTGTCAGCAGCAATTTACTATTGCAAGCTGCTAAGGAACTGTTGAATTTTCTTAG
            ************************************************************
BIG-GENOME  AGCATGCATTCTTTGTCAAGAATGGGTTCCTAGTATATATGAAGATGGATGTAAAAAGCT
BIG-ORF     AGCATGCATTCTTTGTCAAGAATGGGTTCCTAGTATATATGAAGATGGATGTAAAAAGCT
BIG-TAIR    AGCATGCATTCTTTGTCAAGAATGGGTTCCTAGTATATATGAAGATGGATGTAAAAAGCT
            ************************************************************
BIG-GENOME  TGACACTGGTCACATTGATATCCTGCTCAATATAGTAGGCTGTAGTATTGAAGACAAGGC
BIG-ORF     TGACACTGGTCACATTGATATCCTGCTCAATATAGTAGGCTGTAGTATTGAAGACAAGGC
BIG-TAIR    TGACACTGGTCACATTGATATCCTGCTCAATATAGTAGGCTGTAGTATTGAAGACAAGGC
            ************************************************************
BIG-GENOME  TTCTGATGGTGGTTGCATGCTCCAAGATGAGGGAAGACCTGGACATGTTGCTTTTGAGCT
BIG-ORF     TTCTGATGGTGGTTGCATGCTCCAAGATGAGGGAAGACCTGGACATGTTGCTTTTGAGCT
BIG-TAIR    TTCTGATGGTGGTTGCATGCTCCAAGATGAGGGAAGACCTGGACATGTTGCTTTTGAGCT
            ************************************************************
BIG-GENOME  GTTACTCAATCTCTTGAGAAGTCGTGCTCTGTCTGATTTTCTAGAGTCCTACCTTTTTCA
BIG-ORF     GTTACTCAATCTCTTGAGAAGTCGTGCTCTGTCTGATTTTCTAGAGTCCTACCTTTTTCA
BIG-TAIR    GTTACTCAATCTCTTGAGAAGTCGTGCTCTGTCTGATTTTCTAGAGTCCTACCTTTTTCA
            ************************************************************
BIG-GENOME  GCAAATTCTTGTTGTTGAAAATAGTGACTTCAACTATAATGATAAGACTCTGGCACTGTT
BIG-ORF     GCAAATTCTTGTTGTTGAAAATAGTGACTTCAACTATAATGATAAGACTCTGGCACTGTT
BIG-TAIR    GCAAATTCTTGTTGTTGAAAATAGTGACTTCAACTATAATGATAAGACTCTGGCACTGTT
            ************************************************************
BIG-GENOME  GGCTCACACTCTTCTATGTAGGCCGGGCTTGGCTGGGGCACAGTTGAGAGCCAAAATTTA
BIG-ORF     GGCTCACACTCTTCTATGTAGGCCGGGCTTGGCTGGGGCACAGTTGAGAGCCAAAATTTA
BIG-TAIR    GGCTCACACTCTTCTATGTAGGCCGGGCTTGGCTGGGGCACAGTTGAGAGCCAAAATTTA
            ************************************************************
BIG-GENOME  TGATGGTTTCGTTAGTTTTGTTACCGAGAGAGCAAGAGGTATATGTGCTGAAGCTTTGAG
BIG-ORF     TGATGGTTTCGTTAGTTTTGTTACCGAGAGAGCAAGAGGTATATGTGCTGAAGCTTTGAG
BIG-TAIR    TGATGGTTTCGTTAGTTTTGTTACCGAGAGAGCAAGAGGTATATGTGCTGAAGCTTTGAG
            ************************************************************
BIG-GENOME  TCTCAAGGAGTTGACTGCTTGTCTTCCTTCTGCTTTCCATATTGAGATTCTTCTTATGGC
BIG-ORF     TCTCAAGGAGTTGACTGCTTGTCTTCCTTCTGCTTTCCATATTGAGATTCTTCTTATGGC
BIG-TAIR    TCTCAAGGAGTTGACTGCTTGTCTTCCTTCTGCTTTCCATATTGAGATTCTTCTTATGGC
```

```
                ************************************************************
BIG-GENOME      TTTCCATTTATCAAATGAAGCTGAGAAGGCAAAATTCTCAAATCTCATTGCTTCATGTCT
BIG-ORF         TTTCCATTTATCAAATGAAGCTGAGAAGGCAAAATTCTCAAATCTCATTGCTTCATGTCT
BIG-TAIR        TTTCCATTTATCAAATGAAGCTGAGAAGGCAAAATTCTCAAATCTCATTGCTTCATGTCT
                ************************************************************
BIG-GENOME      GCATAAAGTTGATACCCCGGCAGGAATATGTGATGGTCCTCAGCTGTCCTCTTGGGCAAT
BIG-ORF         GCATAAAGTTGATACCCCGGCAGGAATATGTGATGGTCCTCAGCTGTCCTCTTGGGCAAT
BIG-TAIR        GCATAAAGTTGATACCCCGGCAGGAATATGTGATGGTCCTCAGCTGTCCTCTTGGGCAAT
                ************************************************************
BIG-GENOME      GCTGATATCTAGGTTGTTAGTGCTGTTGCACCATATGTTGTTACATCCAAACACATGCCC
BIG-ORF         GCTGATATCTAGGTTGTTAGTGCTGTTGCACCATATGTTGTTACATCCAAACACATGCCC
BIG-TAIR        GCTGATATCTAGGTTGTTAGTGCTGTTGCACCATATGTTGTTACATCCAAACACATGCCC
                ************************************************************
BIG-GENOME      AACATCATTAATGCTAGATCTAAGGTCTAAACTGAGGGAAGTTCGTAGCTGTGGTAGTAA
BIG-ORF         AACATCATTAATGCTAGATCTAAGGTCTAAACTGAGGGAAGTTCGTAGCTGTGGTAGTAA
BIG-TAIR        AACATCATTAATGCTAGATCTAAGGTCTAAACTGAGGGAAGTTCGTAGCTGTGGTAGTAA
                ************************************************************
BIG-GENOME      TTTACATGTAACTGTTGGTGATCATTTATCTTCGTGGGCGTCTCTTGTAGCAAGGGGTAT
BIG-ORF         TTTACATGTAACTGTTGGTGATCATTTATCTTCGTGGGCGTCTCTTGTAGCAAGGGGTAT
BIG-TAIR        TTTACATGTAACTGTTGGTGATCATTTATCTTCGTGGGCGTCTCTTGTAGCAAGGGGTAT
                ************************************************************
BIG-GENOME      AACTGATTCATGGGCTGAGGATGAGTCAGTCAGCCATCTAATGAGTCAAATGATTGACTT
BIG-ORF         AACTGATTCATGGGCTGAGGATGAGTCAGTCAGCCATCTAATGAGTCAAATGATTGACTT
BIG-TAIR        AACTGATTCATGGGCTGAGGATGAGTCAGTCAGCCATCTAATGAGTCAAATGATTGACTT
                ************************************************************
BIG-GENOME      TTCCCCTCATCCTCCTACATTTCAGAATGATGTGTCTACTGCCAAAACCTTAAACTTGGA
BIG-ORF         TTCCCCTCATCCTCCTACATTTCAGAATGATGTGTCTACTGCCAAAACCTTAAACTTGGA
BIG-TAIR        TTCCCCTCATCCTCCTACATTTCAGAATGATGTGTCTACTGCCAAAACCTTAAACTTGGA
                ************************************************************
BIG-GENOME      TTATGGAGATTTATCTGCGAGTTTGTGTCGGGTTTTGGGGCTGTGGAAGGGGAAAAAGGC
BIG-ORF         TTATGGAGATTTATCTGCGAGTTTGTGTCGGGTTTTGGGGCTGTGGAAGGGGAAAAAGGC
BIG-TAIR        TTATGGAGATTTATCTGCGAGTTTGTGTCGGGTTTTGGGGCTGTGGAAGGGGAAAAAGGC
                ************************************************************
BIG-GENOME      AGGAAAAGTGGAAGACCTGCTAGTGGAAAGATACATTTTCATGCTTTCTTCAGATATTGC
BIG-ORF         AGGAAAAGTGGAAGACCTGCTAGTGGAAAGATACATTTTCATGCTTTCTTCAGATATTGC
BIG-TAIR        AGGAAAAGTGGAAGACCTGCTAGTGGAAAGATACATTTTCATGCTTTCTTCAGATATTGC
                ************************************************************
BIG-GENOME      TCGTATTAACTGTGCATTGGATAGCCAGCCTTCTTTGCATGTTAATTACCAGAATGTGGA
BIG-ORF         TCGTATTAACTGTGCATTGGATAGCCAGCCTTCTTTGCATGTTAATTACCAGAATGTGGA
BIG-TAIR        TCGTATTAACTGTGCATTGGATAGCCAGCCTTCTTTGCATGTTAATTACCAGAATGTGGA
                ************************************************************
BIG-GENOME      CATATCTAACTCTGTGGATCTCATCAGCACCAGTCATTTGCTTGTAGGCGACATCAATGT
BIG-ORF         CATATCTAACTCTGTGGATCTCATCAGCACCAGTCATTTGCTTGTAGGCGACATCAATGT
BIG-TAIR        CATATCTAACTCTGTGGATCTCATCAGCACCAGTCATTTGCTTGTAGGCGACATCAATGT
```

```
                ************************************************************
BIG-GENOME      TGTTGGTAGAAATATCGAGTTGAGAAACATTCTGATCGGTGTTTTAAATCAGCTCCAGGC
BIG-ORF         TGTTGGTAGAAATATCGAGTTGAGAAACATTCTGATCGGTGTTTTAAATCAGCTCCAGGC
BIG-TAIR        TGTTGGTAGAAATATCGAGTTGAGAAACATTCTGATCGGTGTTTTAAATCAGCTCCAGGC
                ************************************************************
BIG-GENOME      AGCACCTGAGCAGGTGGTTGAGGATTTGGGTTGGGATTATATTCGCGAAGGAGCTTGGCT
BIG-ORF         AGCACCTGAGCAGGTGGTTGAGGATTTGGGTTGGGATTATATTCGCGAAGGAGCTTGGCT
BIG-TAIR        AGCACCTGAGCAGGTGGTTGAGGATTTGGGTTGGGATTATATTCGCGAAGGAGCTTGGCT
                ************************************************************
BIG-GENOME      CTCTCTTCTGTTGTACTTTCTTGATGGTGGCGTCTGGGATTATTGCAACAAAAATTCATG
BIG-ORF         CTCTCTTCTGTTGTACTTTCTTGATGGTGGCGTCTGGGATTATTGCAACAAAAATTCATG
BIG-TAIR        CTCTCTTCTGTTGTACTTTCTTGATGGTGGCGTCTGGGATTATTGCAACAAAAATTCATG
                ************************************************************
BIG-GENOME      TTCAGAAATCGATCCCTTCTGGAAGGAGTGCACATCTGTTGACGCCAAGTATGTTGCTGC
BIG-ORF         TTCAGAAATCGATCCCTTCTGGAAGGAGTGCACATCTGTTGACGCCAAGTATGTTGCTGC
BIG-TAIR        TTCAGAAATCGATCCCTTCTGGAAGGAGTGCACATCTGTTGACGCCAAGTATGTTGCTGC
                ************************************************************
BIG-GENOME      AGCAGAAGGGGTAGTCTCTTACTTGATGAAGACTGGTGATATTGCAGAATTGCTGAGAAT
BIG-ORF         AGCAGAAGGGGTAGTCTCTTACTTGATGAAGACTGGTGATATTGCAGAATTGCTGAGAAT
BIG-TAIR        AGCAGAAGGGGTAGTCTCTTACTTGATGAAGACTGGTGATATTGCAGAATTGCTGAGAAT
                ************************************************************
BIG-GENOME      GCTTTCATCATTGGTTGGCAAATATTTACGAGTGTATAAGAAAGCTTTCCTTGCAACTTT
BIG-ORF         GCTTTCATCATTGGTTGGCAAATATTTACGAGTGTATAAGAAAGCTTTCCTTGCAACTTT
BIG-TAIR        GCTTTCATCATTGGTTGGCAAATATTTACGAGTGTATAAGAAAGCTTTCCTTGCAACTTT
                ************************************************************
BIG-GENOME      CAGCGATTGGAATCATCACGGTCATAGTTCGCCTTCTCTGCTACTTCTCAAGCATACTCA
BIG-ORF         CAGCGATTGGAATCATCACGGTCATAGTTCGCCTTCTCTGCTACTTCTCAAGCATACTCA
BIG-TAIR        CAGCGATTGGAATCATCACGGTCATAGTTCGCCTTCTCTGCTACTTCTCAAGCATACTCA
                ************************************************************
BIG-GENOME      ATTTGGTAAAAGTCTCCAAGGTGAATATGCGAAGATTGGTGACAATTCTCTCCATCTTCA
BIG-ORF         ATTTGGTAAAAGTCTCCAAGGTGAATATGCGAAGATTGGTGACAATTCTCTCCATCTTCA
BIG-TAIR        ATTTGGTAAAAGTCTCCAAGGTGAATATGCGAAGATTGGTGACAATTCTCTCCATCTTCA
                ************************************************************
BIG-GENOME      ATGCATCTTTTATCTGTCAAAACTGGATTCCCTGGGAGATGGAAGAGGTTCGGGTGTTTT
BIG-ORF         ATGCATCTTTTATCTGTCAAAACTGGATTCCCTGGGAGATGGAAGAGGTTCGGGTGTTTT
BIG-TAIR        ATGCATCTTTTATCTGTCAAAACTGGATTCCCTGGGAGATGGAAGAGGTTCGGGTGTTTT
                ************************************************************
BIG-GENOME      ATGGAAAGTGTTTTGGGAATTTATGGTACATGGGTTTCCCACTAGTCTTCAAACTTCTAG
BIG-ORF         ATGGAAAGTGTTTTGGGAATTTATGGTACATGGGTTTCCCACTAGTCTTCAAACTTCTAG
BIG-TAIR        ATGGAAAGTGTTTTGGGAATTTATGGTACATGGGTTTCCCACTAGTCTTCAAACTTCTAG
                ************************************************************
BIG-GENOME      TGCGATTCTTCTTTCATGTATTCTAAGCATAAGATGCATTGTGCTGACAATAAATGGCTT
BIG-ORF         TGCGATTCTTCTTTCATGTATTCTAAGCATAAGATGCATTGTGCTGACAATAAATGGCTT
BIG-TAIR        TGCGATTCTTCTTTCATGTATTCTAAGCATAAGATGCATTGTGCTGACAATAAATGGCTT
```

```
            ************************************************************
BIG-GENOME  ACTCAAACTGGGTAACTCTAAGGAGAAGTTTGGGGTAGATACTAGTGTACTTCATCAGCT
BIG-ORF     ACTCAAACTGGGTAACTCTAAGGAGAAGTTTGGGGTAGATACTAGTGTACTTCATCAGCT
BIG-TAIR    ACTCAAACTGGGTAACTCTAAGGAGAAGTTTGGGGTAGATACTAGTGTACTTCATCAGCT
            ************************************************************
BIG-GENOME  ACTTGATTCTATCATGATTATTAAGTTTGACCAGGTATTCGAAAGCTTTCATGGGAAATG
BIG-ORF     ACTTGATTCTATCATGATTATTAAGTTTGACCAGGTATTCGAAAGCTTTCATGGGAAATG
BIG-TAIR    ACTTGATTCTATCATGATTATTAAGTTTGACCAGGTATTCGAAAGCTTTCATGGGAAATG
            ************************************************************
BIG-GENOME  TGAGGAGATCCATCAAAATATATGTGCCGTGTTGCAGCTTCCAGATTTGACTGAGTTGTT
BIG-ORF     TGAGGAGATCCATCAAAATATATGTGCCGTGTTGCAGCTTCCAGATTTGACTGAGTTGTT
BIG-TAIR    TGAGGAGATCCATCAAAATATATGTGCCGTGTTGCAGCTTCCAGATTTGACTGAGTTGTT
            ************************************************************
BIG-GENOME  TCTGATGAAAGACATGGAGGGGTTTGTAAGAGATATCAGTGCTGAGCAGATAGATAGAAG
BIG-ORF     TCTGATGAAAGACATGGAGGGGTTTGTAAGAGATATCAGTGCTGAGCAGATAGATAGAAG
BIG-TAIR    TCTGATGAAAGACATGGAGGGGTTTGTAAGAGATATCAGTGCTGAGCAGATAGATAGAAG
            ************************************************************
BIG-GENOME  CCAAGTGCTTGAAGGGGTGATCACCAAGATTGTAGATGTTATGGACAGTCTAAGCAAGGA
BIG-ORF     CCAAGTGCTTGAAGGGGTGATCACCAAGATTGTAGATGTTATGGACAGTCTAAGCAAGGA
BIG-TAIR    CCAAGTGCTTGAAGGGGTGATCACCAAGATTGTAGATGTTATGGACAGTCTAAGCAAGGA
            ************************************************************
BIG-GENOME  TTCTTCGAAATCTGATATTTTCAAATTTTATCTTGGTGTAGATGCAGTCTCTGAGCATAC
BIG-ORF     TTCTTCGAAATCTGATATTTTCAAATTTTATCTTGGTGTAGATGCAGTCTCTGAGCATAC
BIG-TAIR    TTCTTCGAAATCTGATATTTTCAAATTTTATCTTGGTGTAGATGCAGTCTCTGAGCATAC
            ************************************************************
BIG-GENOME  CAGGGAATTTTATGAGTTGCAACGTGGAGATCTGTCTGTGTTTATTGACTCATTGGACTA
BIG-ORF     CAGGGAATTTTATGAGTTGCAACGTGGAGATCTGTCTGTGTTTATTGACTCATTGGACTA
BIG-TAIR    CAGGGAATTTTATGAGTTGCAACGTGGAGATCTGTCTGTGTTTATTGACTCATTGGACTA
            ************************************************************
BIG-GENOME  CTGTTCCTTGGAACCAGTAAACATAAAAGTACTTAACTTCCTTGTTGACCTTTTGTCTGT
BIG-ORF     CTGTTCCTTGGAACCAGTAAACATAAAAGTACTTAACTTCCTTGTTGACCTTTTGTCTGT
BIG-TAIR    CTGTTCCTTGGAACCAGTAAACATAAAAGTACTTAACTTCCTTGTTGACCTTTTGTCTGT
            ************************************************************
BIG-GENOME  GGCTCAATCCCCTGATCTCCGGAGAAGAGTACAACAAAAATTTATTGACATGGATTTGAT
BIG-ORF     GGCTCAATCCCCTGATCTCCGGAGAAGAGTACAACAAAAATTTATTGACATGGATTTGAT
BIG-TAIR    GGCTCAATCCCCTGATCTCCGGAGAAGAGTACAACAAAAATTTATTGACATGGATTTGAT
            ************************************************************
BIG-GENOME  ATCTTTGTCTGGGTGGCTGGAGAGAAGACTGTTGGGTTCCTTCGTTGAAGAAATAGATGG
BIG-ORF     ATCTTTGTCTGGGTGGCTGGAGAGAAGACTGTTGGGTTCCTTCGTTGAAGAAATAGATGG
BIG-TAIR    ATCTTTGTCTGGGTGGCTGGAGAGAAGACTGTTGGGTTCCTTCGTTGAAGAAATAGATGG
            ************************************************************
BIG-GENOME  GAAAAAGACTGCAAAAGGAAATTCTCTTCCTTTCAGAGAGGCAGCAATGAATTTTATTAA
BIG-ORF     GAAAAAGACTGCAAAAGGAAATTCTCTTCCTTTCAGAGAGGCAGCAATGAATTTTATTAA
BIG-TAIR    GAAAAAGACTGCAAAAGGAAATTCTCTTCCTTTCAGAGAGGCAGCAATGAATTTTATTAA
```

```
                ************************************************************
BIG-GENOME      TTGTCTTGTGTCATCTACTAACGACCTACAGACCAGAGAGTTACAGAATCATTTATTCGA
BIG-ORF         TTGTCTTGTGTCATCTACTAACGACCTACAGACCAGAGAGTTACAGAATCATTTATTCGA
BIG-TAIR        TTGTCTTGTGTCATCTACTAACGACCTACAGACCAGAGAGTTACAGAATCATTTATTCGA
                ************************************************************
BIG-GENOME      GGCCTTGCTGATCTCCCTCGATACCGCGTTTCTCTCATTTGATATCCATATGTCTATGTC
BIG-ORF         GGCCTTGCTGATCTCCCTCGATACCGCGTTTCTCTCATTTGATATCCATATGTCTATGTC
BIG-TAIR        GGCCTTGCTGATCTCCCTCGATACCGCGTTTCTCTCATTTGATATCCATATGTCTATGTC
                ************************************************************
BIG-GENOME      ATATTTTCATTTTGTTCTTCAACTTGCAAGGGAAGACAACCTGATGAAGATGGTTTTAAA
BIG-ORF         ATATTTTCATTTTGTTCTTCAACTTGCAAGGGAAGACAACCTGATGAAGATGGTTTTAAA
BIG-TAIR        ATATTTTCATTTTGTTCTTCAACTTGCAAGGGAAGACAACCTGATGAAGATGGTTTTAAA
                ************************************************************
BIG-GENOME      GAGAACCATAATGCTGATGGAGAAGCTTGCTGCCGAGGAGAAGTTGCTTCCTGGTTTGAA
BIG-ORF         GAGAACCATAATGCTGATGGAGAAGCTTGCTGCCGAGGAGAAGTTGCTTCCTGGTTTGAA
BIG-TAIR        GAGAACCATAATGCTGATGGAGAAGCTTGCTGCCGAGGAGAAGTTGCTTCCTGGTTTGAA
                ************************************************************
BIG-GENOME      GTTTATTTTTGGTGTGATAGGCACCTTGTTGAGCAACCGTTCTCCAAGTCATGGAGAGAG
BIG-ORF         GTTTATTTTTGGTGTGATAGGCACCTTGTTGAGCAACCGTTCTCCAAGTCATGGAGAGAG
BIG-TAIR        GTTTATTTTTGGTGTGATAGGCACCTTGTTGAGCAACCGTTCTCCAAGTCATGGAGAGAG
                ************************************************************
BIG-GENOME      TTTGTGTGGAAAGTCCTTGGCAAGCTATAAGAATACTGCAACAGGTCCTTTGGTCCCAAA
BIG-ORF         TTTGTGTGGAAAGTCCTTGGCAAGCTATAAGAATACTGCAACAGGTCCTTTGGTCCCAAA
BIG-TAIR        TTTGTGTGGAAAGTCCTTGGCAAGCTATAAGAATACTGCAACAGGTCCTTTGGTCCCAAA
                ************************************************************
BIG-GENOME      ACTTTCAGGAACAACGAAAAAATCTGATACATTGGCTCTCCCCGTGGATCAGGAAGGAAG
BIG-ORF         ACTTTCAGGAACAACGAAAAAATCTGATACATTGGCTCTCCCCGTGGATCAGGAAGGAAG
BIG-TAIR        ACTTTCAGGAACAACGAAAAAATCTGATACATTGGCTCTCCCCGTGGATCAGGAAGGAAG
                ************************************************************
BIG-GENOME      CTCAATATCACTTGAATGCGATGTCACTTCTGTTGATGAAGATGAAGATGATGGAACATC
BIG-ORF         CTCAATATCACTTGAATGCGATGTCACTTCTGTTGATGAAGATGAAGATGATGGAACATC
BIG-TAIR        CTCAATATCACTTGAATGCGATGTCACTTCTGTTGATGAAGATGAAGATGATGGAACATC
                ************************************************************
BIG-GENOME      TGATGGTGAAGTGGCCAGCTTAGACAAGGAAGATGAAGAAGATGCCAACAGTGAGAGGTA
BIG-ORF         TGATGGTGAAGTGGCCAGCTTAGACAAGGAAGATGAAGAAGATGCCAACAGTGAGAGGTA
BIG-TAIR        TGATGGTGAAGTGGCCAGCTTAGACAAGGAAGATGAAGAAGATGCCAACAGTGAGAGGTA
                ************************************************************
BIG-GENOME      CCTTGCCTCAAAAGTCTGCACTTTTACGTCCAGTGGCAGTAATTTCATGGAACAACACTG
BIG-ORF         CCTTGCCTCAAAAGTCTGCACTTTTACGTCCAGTGGCAGTAATTTCATGGAACAACACTG
BIG-TAIR        CCTTGCCTCAAAAGTCTGCACTTTTACGTCCAGTGGCAGTAATTTCATGGAACAACACTG
                ************************************************************
BIG-GENOME      GTATTTTTGTTACACTTGTGACCTTACTGTGTCCAAAGGTTGCTGTTCTGTTTGCGCGAA
BIG-ORF         GTATTTTTGTTACACTTGTGACCTTACTGTGTCCAAAGGTTGCTGTTCTGTTTGCGCGAA
BIG-TAIR        GTATTTTTGTTACACTTGTGACCTTACTGTGTCCAAAGGTTGCTGTTCTGTTTGCGCGAA
```

```
                ************************************************************
BIG-GENOME      AGTTTGCCACCGGGGTCACCGTGTTGTCTATTCACGATCGAGTCGGTTTTTCTGTGACTG
BIG-ORF         AGTTTGCCACCGGGGTCACCGTGTTGTCTATTCACGATCGAGTCGGTTTTTCTGTGACTG
BIG-TAIR        AGTTTGCCACCGGGGTCACCGTGTTGTCTATTCACGATCGAGTCGGTTTTTCTGTGACTG
                ************************************************************
BIG-GENOME      TGGAGCTGGAGGTGTTAGGGGAAGCAGCTGCCAGTGCCTGAAGCCACGCAAATATAATGG
BIG-ORF         TGGAGCTGGAGGTGTTAGGGGAAGCAGCTGCCAGTGCCTGAAGCCACGCAAATATAATGG
BIG-TAIR        TGGAGCTGGAGGTGTTAGGGGAAGCAGCTGCCAGTGCCTGAAGCCACGCAAATATAATGG
                ************************************************************
BIG-GENOME      AAATGGAAGTGCTCCAGCTCGTGGTACAAATAATTTCCAATCGTTTTTACCCTTGTCTGA
BIG-ORF         AAATGGAAGTGCTCCAGCTCGTGGTACAAATAATTTCCAATCGTTTTTACCCTTGTCTGA
BIG-TAIR        AAATGGAAGTGCTCCAGCTCGTGGTACAAATAATTTCCAATCGTTTTTACCCTTGTCTGA
                ************************************************************
BIG-GENOME      GGATGCAGATCAGCTTGGAGAAAGCGATTCGGATGTGGAAGAAGATGGTTTCGGGGAGGA
BIG-ORF         GGATGCAGATCAGCTTGGAGAAAGCGATTCGGATGTGGAAGAAGATGGTTTCGGGGAGGA
BIG-TAIR        GGATGCAGATCAGCTTGGAGAAAGCGATTCGGATGTGGAAGAAGATGGTTTCGGGGAGGA
                ************************************************************
BIG-GENOME      AAATCACGTTGTCTTATATATCCCTAAAGAAACTCAATATAAGATGTCACTGCTACTCGA
BIG-ORF         AAATCACGTTGTCTTATATATCCCTAAAGAAACTCAATATAAGATGTCACTGCTACTCGA
BIG-TAIR        AAATCACGTTGTCTTATATATCCCTAAAGAAACTCAATATAAGATGTCACTGCTACTCGA
                ************************************************************
BIG-GENOME      AGAGCTCGGGATAGAGGACCGAGTACTGGAGCTTTTCTCATCTTTACTTCCGTCTATCAC
BIG-ORF         AGAGCTCGGGATAGAGGACCGAGTACTGGAGCTTTTCTCATCTTTACTTCCGTCTATCAC
BIG-TAIR        AGAGCTCGGGATAGAGGACCGAGTACTGGAGCTTTTCTCATCTTTACTTCCGTCTATCAC
                ************************************************************
BIG-GENOME      TAGCAAAAGAGACTCTGGCCTGTCGAAAGAGAAGCAGGTCAATCTCGGGAAAGACAAAGT
BIG-ORF         TAGCAAAAGAGACTCTGGCCTGTCGAAAGAGAAGCAGGTCAATCTCGGGAAAGACAAAGT
BIG-TAIR        TAGCAAAAGAGACTCTGGCCTGTCGAAAGAGAAGCAGGTCAATCTCGGGAAAGACAAAGT
                ************************************************************
BIG-GENOME      TCTTTCATTTGACACGGATCTTTTGCAGCTGAAAAAAGCATATAAAAGTGGGTCATTGGA
BIG-ORF         TCTTTCATTTGACACGGATCTTTTGCAGCTGAAAAAAGCATATAAAAGTGGGTCATTGGA
BIG-TAIR        TCTTTCATTTGACACGGATCTTTTGCAGCTGAAAAAAGCATATAAAAGTGGGTCATTGGA
                ************************************************************
BIG-GENOME      CTTAAAAATAAAGGCTGATTATACTAATTCAAAGGACCTTAAATCTCTTTTAGCCAATGG
BIG-ORF         CTTAAAAATAAAGGCTGATTATACTAATTCAAAGGACCTTAAATCTCTTTTAGCCAATGG
BIG-TAIR        CTTAAAAATAAAGGCTGATTATACTAATTCAAAGGACCTTAAATCTCTTTTAGCCAATGG
                ************************************************************
BIG-GENOME      TTCCCTTGTCAAGTCTCTCCTGAGTGTTAGTGTTCGGGGTCGCCTTGCAGTTGGAGAAGG
BIG-ORF         TTCCCTTGTCAAGTCTCTCCTGAGTGTTAGTGTTCGGGGTCGCCTTGCAGTTGGAGAAGG
BIG-TAIR        TTCCCTTGTCAAGTCTCTCCTGAGTGTTAGTGTTCGGGGTCGCCTTGCAGTTGGAGAAGG
                ************************************************************
BIG-GENOME      TGATAAAGTTGCTATATTTGATGTTGGACAGCTAATAGGACAAGCCACAATTGCACCCAT
BIG-ORF         TGATAAAGTTGCTATATTTGATGTTGGACAGCTAATAGGACAAGCCACAATTGCACCCAT
BIG-TAIR        TGATAAAGTTGCTATATTTGATGTTGGACAGCTAATAGGACAAGCCACAATTGCACCCAT
```

```
            ************************************************************
BIG-GENOME  AAATGCAGACAAGGCCAACGTTAAACCACTTTCAAGGAATATTGTTCGTTTTGAGATTGT
BIG-ORF     AAATGCAGACAAGGCCAACGTTAAACCACTTTCAAGGAATATTGTTCGTTTTGAGATTGT
BIG-TAIR    AAATGCAGACAAGGCCAACGTTAAACCACTTTCAAGGAATATTGTTCGTTTTGAGATTGT
            ************************************************************
BIG-GENOME  GCATCTTTCATTCAACCCAGTTGTGGAGAATTATCTTGCTGTCGCAGGCCTTGAAGATTG
BIG-ORF     GCATCTTTCATTCAACCCAGTTGTGGAGAATTATCTTGCTGTCGCAGGCCTTGAAGATTG
BIG-TAIR    GCATCTTTCATTCAACCCAGTTGTGGAGAATTATCTTGCTGTCGCAGGCCTTGAAGATTG
            ************************************************************
BIG-GENOME  CCAGATACTTACTTTGAATCATCGAGGTGAAGTCATCGACAGGCTTGCTGTTGAGCTTGC
BIG-ORF     CCAGATACTTACTTTGAATCATCGAGGTGAAGTCATCGACAGGCTTGCTGTTGAGCTTGC
BIG-TAIR    CCAGATACTTACTTTGAATCATCGAGGTGAAGTCATCGACAGGCTTGCTGTTGAGCTTGC
            ************************************************************
BIG-GENOME  CCTGCAAGGTGCATTCATCAGGCGTATAGACTGGGTTCCTGGTTCACAGGTTCAGTTAAT
BIG-ORF     CCTGCAAGGTGCATTCATCAGGCGTATAGACTGGGTTCCTGGTTCACAGGTTCAGTTAAT
BIG-TAIR    CCTGCAAGGTGCATTCATCAGGCGTATAGACTGGGTTCCTGGTTCACAGGTTCAGTTAAT
            ************************************************************
BIG-GENOME  GGTCGTTACGAACAAATTCGTGAAGATTTATGATCTATCCCAGGATAGCATCAGTCCAAC
BIG-ORF     GGTCGTTACGAACAAATTCGTGAAGATTTATGATCTATCCCAGGATAGCATCAGTCCAAC
BIG-TAIR    GGTCGTTACGAACAAATTCGTGAAGATTTATGATCTATCCCAGGATAGCATCAGTCCAAC
            ************************************************************
BIG-GENOME  ACAGTACTTCACTTTGCCAAACGACATGATTGTGGATGCTACTCTTTTTGTTGCTTCTCG
BIG-ORF     ACAGTACTTCACTTTGCCAAACGACATGATTGTGGATGCTACTCTTTTTGTTGCTTCTCG
BIG-TAIR    ACAGTACTTCACTTTGCCAAACGACATGATTGTGGATGCTACTCTTTTTGTTGCTTCTCG
            ************************************************************
BIG-GENOME  TGGGAGGGTTTTCCTTCTTGTTCTTTCAGAACAAGGGAATTTGTATAGGTTCGAACTATC
BIG-ORF     TGGGAGGGTTTTCCTTCTTGTTCTTTCAGAACAAGGGAATTTGTATAGGTTCGAACTATC
BIG-TAIR    TGGGAGGGTTTTCCTTCTTGTTCTTTCAGAACAAGGGAATTTGTATAGGTTCGAACTATC
            ************************************************************
BIG-GENOME  TTGGGGCGGCAATGCAGGCGCAACACCGCTTAAGGAAATCGTTCAGATTATGGGAAAGGA
BIG-ORF     TTGGGGCGGCAATGCAGGCGCAACACCGCTTAAGGAAATCGTTCAGATTATGGGAAAGGA
BIG-TAIR    TTGGGGCGGCAATGCAGGCGCAACACCGCTTAAGGAAATCGTTCAGATTATGGGAAAGGA
            ************************************************************
BIG-GENOME  TGTTACGGGAAAGGGTTCATCTGTCTATTTCTCTCCAACATATCGACTGCTTTTCATATC
BIG-ORF     TGTTACGGGAAAGGGTTCATCTGTCTATTTCTCTCCAACATATCGACTGCTTTTCATATC
BIG-TAIR    TGTTACGGGAAAGGGTTCATCTGTCTATTTCTCTCCAACATATCGACTGCTTTTCATATC
            ************************************************************
BIG-GENOME  CTATCATGATGGAAGTTCTTTTATGGGTCGACTCAGCTCAGATGCAACATCCTTAACTGA
BIG-ORF     CTATCATGATGGAAGTTCTTTTATGGGTCGACTCAGCTCAGATGCAACATCCTTAACTGA
BIG-TAIR    CTATCATGATGGAAGTTCTTTTATGGGTCGACTCAGCTCAGATGCAACATCCTTAACTGA
            ************************************************************
BIG-GENOME  TACATCTGGCATGTTTGAGGAAGAATCAGATTGTAAACAAAGGGTGGCTGGATTGCATCG
BIG-ORF     TACATCTGGCATGTTTGAGGAAGAATCAGATTGTAAACAAAGGGTGGCTGGATTGCATCG
BIG-TAIR    TACATCTGGCATGTTTGAGGAAGAATCAGATTGTAAACAAAGGGTGGCTGGATTGCATCG
```

```
            ************************************************************
BIG-GENOME  TTGGAAAGAGTTGTTGGCTGGCAGTGGATTATTTATTTGCTTCTCCAGCGTGAAGTCAAA
BIG-ORF     TTGGAAAGAGTTGTTGGCTGGCAGTGGATTATTTATTTGCTTCTCCAGCGTGAAGTCAAA
BIG-TAIR    TTGGAAAGAGTTGTTGGCTGGCAGTGGATTATTTATTTGCTTCTCCAGCGTGAAGTCAAA
            ************************************************************
BIG-GENOME  TGCTGTCTTAGCTGTGTCCTTGAGGGGCGATGGGGTATGTGCACAGAATCTCCGTCATCC
BIG-ORF     TGCTGTCTTAGCTGTGTCCTTGAGGGGCGATGGGGTATGTGCACAGAATCTCCGTCATCC
BIG-TAIR    TGCTGTCTTAGCTGTGTCCTTGAGGGGCGATGGGGTATGTGCACAGAATCTCCGTCATCC
            ************************************************************
BIG-GENOME  GACAGGATCATCTTCCCCTATGGTTGGAATAACCGCATACAAACCTTTGTCAAAAGACAA
BIG-ORF     GACAGGATCATCTTCCCCTATGGTTGGAATAACCGCATACAAACCTTTGTCAAAAGACAA
BIG-TAIR    GACAGGATCATCTTCCCCTATGGTTGGAATAACCGCATACAAACCTTTGTCAAAAGACAA
            ************************************************************
BIG-GENOME  TGTTCACTGTCTAGTTCTGCATGATGATGGCAGCCTTCAGATTTATTCTCATGTTCGTAG
BIG-ORF     TGTTCACTGTCTAGTTCTGCATGATGATGGCAGCCTTCAGATTTATTCTCATGTTCGTAG
BIG-TAIR    TGTTCACTGTCTAGTTCTGCATGATGATGGCAGCCTTCAGATTTATTCTCATGTTCGTAG
            ************************************************************
BIG-GENOME  TGGAGTTGATACTGACTCAAATTTCACAGCTGAAAAAGTTAAAAAGTTGGGTTCTAAGAT
BIG-ORF     TGGAGTTGATACTGACTCAAATTTCACAGCTGAAAAAGTTAAAAAGTTGGGTTCTAAGAT
BIG-TAIR    TGGAGTTGATACTGACTCAAATTTCACAGCTGAAAAAGTTAAAAAGTTGGGTTCTAAGAT
            ************************************************************
BIG-GENOME  ACTTAACAACAAAACCTATGCTGGTGCAAAGCCAGAGTTTCCCCTGGATTTCTTTGAGAG
BIG-ORF     ACTTAACAACAAAACCTATGCTGGTGCAAAGCCAGAGTTTCCCCTGGATTTCTTTGAGAG
BIG-TAIR    ACTTAACAACAAAACCTATGCTGGTGCAAAGCCAGAGTTTCCCCTGGATTTCTTTGAGAG
            ************************************************************
BIG-GENOME  GGCATTTTGCATTACAGCAGATGTGAGACTTGGTAGTGATGCTATTAGAAATGGTGATTC
BIG-ORF     GGCATTTTGCATTACAGCAGATGTGAGACTTGGTAGTGATGCTATTAGAAATGGTGATTC
BIG-TAIR    GGCATTTTGCATTACAGCAGATGTGAGACTTGGTAGTGATGCTATTAGAAATGGTGATTC
            ************************************************************
BIG-GENOME  CGAGGGAGCAAAACAGAGCTTGGCATCTGAGGATGGCTTTATTGAGAGCCCCAGTCCCGT
BIG-ORF     CGAGGGAGCAAAACAGAGCTTGGCATCTGAGGATGGCTTTATTGAGAGCCCCAGTCCCGT
BIG-TAIR    CGAGGGAGCAAAACAGAGCTTGGCATCTGAGGATGGCTTTATTGAGAGCCCCAGTCCCGT
            ************************************************************
BIG-GENOME  GGGCTTCAAGGTATGGTTGGAATTCTGCAGTAATTTTTCTTGATAAGTTTATCTGCATAC
BIG-ORF     GGGCTTCAAG--------------------------------------------------
BIG-TAIR    GGGCTTCAAG--------------------------------------------------
            **********
BIG-GENOME  TATGCTTGGTTATTAGTTTAGTACTCTGAAGAGTATTTTGTCCGCCCAATTATGAGTATA
BIG-ORF     ------------------------------------------------------------
BIG-TAIR    ------------------------------------------------------------

BIG-GENOME  TGGGTTGCTTGTCTCCAGTTATCTAACCTCCTTCTCTCTTTCAGATATCTGTCTCCAATC
BIG-ORF     --------------------------------------------ATATCTGTCTCCAATC
BIG-TAIR    --------------------------------------------ATATCTGTCTCCAATC
```

```
                                                    ****************
BIG-GENOME    CAAACCCTGACATTGTTATGGTTGGCATCCGGATGCATGTGGGTACTACATCAGCAAGCT
BIG-ORF       CAAACCCTGACATTGTTATGGTTGGCATCCGGATGCATGTGGGTACTACATCAGCAAGCT
BIG-TAIR      CAAACCCTGACATTGTTATGGTTGGCATCCGGATGCATGTGGGTACTACATCAGCAAGCT
              ************************************************************

BIG-GENOME    CTATACCTTCAGAAGTGACCATTTTCCAGAGATCGATTAAGATGGATGAGGGCATGAGGT
BIG-ORF       CTATACCTTCAGAAGTGACCATTTTCCAGAGATCGATTAAGATGGATGAGGGCATGAGGT
BIG-TAIR      CTATACCTTCAGAAGTGACCATTTTCCAGAGATCGATTAAGATGGATGAGGGCATGAGGT
              ************************************************************
BIG-GENOME    GCTGGTATGACATCCCATTTACTGTGGCTGAGTCACTTCTAGCTGATGAAGATGTTGTAA
BIG-ORF       GCTGGTATGACATCCCATTTACTGTGGCTGAGTCACTTCTAGCTGATGAAGATGTTGTAA
BIG-TAIR      GCTGGTATGACATCCCATTTACTGTGGCTGAGTCACTTCTAGCTGATGAAGATGTTGTAA
              ************************************************************
BIG-GENOME    TCTCTGTGGGGCCGACTACTAGTGGGACTGCACTGCCCAGAATAGACTCGCTTGAAGTAT
BIG-ORF       TCTCTGTGGGGCCGACTACTAGTGGGACTGCACTGCCCAGAATAGACTCGCTTGAAGTAT
BIG-TAIR      TCTCTGTGGGGCCGACTACTAGTGGGACTGCACTGCCCAGAATAGACTCGCTTGAAGTAT
              ************************************************************
BIG-GENOME    ATGGTCGAGCTAAAGATGAATTTGGCTGGAAAGAAAAAATGGATGCTGTGCTAGATATGG
BIG-ORF       ATGGTCGAGCTAAAGATGAATTTGGCTGGAAAGAAAAAATGGATGCTGTGCTAGATATGG
BIG-TAIR      ATGGTCGAGCTAAAGATGAATTTGGCTGGAAAGAAAAAATGGATGCTGTGCTAGATATGG
              ************************************************************
BIG-GENOME    AAGCTCGTGTGCTTGGTCATGGTTTGCTTCTTCCAGGCTCTAGTAAAAAGAGAGCGTTGG
BIG-ORF       AAGCTCGTGTGCTTGGTCATGGTTTGCTTCTTCCAGGCTCTAGTAAAAAGAGAGCGTTGG
BIG-TAIR      AAGCTCGTGTGCTTGGTCATGGTTTGCTTCTTCCAGGCTCTAGTAAAAAGAGAGCGTTGG
              ************************************************************
BIG-GENOME    CACAGTCTGCTTCAATGGAAGAGCAAGTTATCGCTGATGGTCTTAAGCTCCTATCAATCT
BIG-ORF       CACAGTCTGCTTCAATGGAAGAGCAAGTTATCGCTGATGGTCTTAAGCTCCTATCAATCT
BIG-TAIR      CACAGTCTGCTTCAATGGAAGAGCAAGTTATCGCTGATGGTCTTAAGCTCCTATCAATCT
              ************************************************************
BIG-GENOME    ATTATTCAGTTTGTAGGCCACGGCAAGAAGTAGTGCTTAGCGAACTCAAATGCAAACAGC
BIG-ORF       ATTATTCAGTTTGTAGGCCACGGCAAGAAGTAGTGCTTAGCGAACTCAAATGCAAACAGC
BIG-TAIR      ATTATTCAGTTTGTAGGCCACGGCAAGAAGTAGTGCTTAGCGAACTCAAATGCAAACAGC
              ************************************************************
BIG-GENOME    TACTAGAGACGATTTTCGAAAGTGATAGGGAAACCCTATTACAAACGACAGCTTGCCGTG
BIG-ORF       TACTAGAGACGATTTTCGAAAGTGATAGGGAAACCCTATTACAAACGACAGCTTGCCGTG
BIG-TAIR      TACTAGAGACGATTTTCGAAAGTGATAGGGAAACCCTATTACAAACGACAGCTTGCCGTG
              ************************************************************
BIG-GENOME    TTTTGCAGTCTGTCTTTCCAAGAAAGGAGATATACTACCAGGTAATGTTTCTACCAAATT
BIG-ORF       TTTTGCAGTCTGTCTTTCCAAGAAAGGAGATATACTACCAG-------------------
BIG-TAIR      TTTTGCAGTCTGTCTTTCCAAGAAAGGAGATATACTACCAGGTAATGTTTCTACCAAATT
              *****************************************
BIG-GENOME    CAGTCTTGCATGTTCAGTAGATATTTCCGGGCGCAGATATTCTTTAAATTTGTATGTTGT
BIG-ORF       ------------------------------------------------------------
```

```
BIG-TAIR      CAG---------------------------------------------------------

BIG-GENOME    CTTTGGCATTGATTATATCTGTTGCTATAGGTCAAAGACACCATGCGACTCCTTGGAGTG
BIG-ORF       ------------------------------GTCAAAGACACCATGCGACTCCTTGGAGTG
BIG-TAIR      -------------------------------TCAAAGACACCATGCGACTCCTTGGAGTG
                                             *****************************
BIG-GENOME    GTCAAGGTGACCTCCATTCTTTCTTCGAGGTTGGGGATTTTGGGTACTGGAGGTTCGATT
BIG-ORF       GTCAAGGTGACCTCCATTCTTTCTTCGAGGTTGGGGATTTTGGGTACTGGAGGTTCGATT
BIG-TAIR      GTCAAGGTGACCTCCATTCTTTCTTCGAGGTTGGGGATTTTGGGTACTGGAGGTTCGATT
              ************************************************************
BIG-GENOME    GTTGAAGAGTTCAATGCTCAGATGCGGGCGGTGTCTAAAGTAGCTTTGACCCGTAAATCA
BIG-ORF       GTTGAAGAGTTCAATGCTCAGATGCGGGCGGTGTCTAAAGTAGCTTTGACCCGTAAATCA
BIG-TAIR      GTTGAAGAGTTCAATGCTCAGATGCGGGCGGTGTCTAAAGTAGCTTTGACCCGTAAATCA
              ************************************************************
BIG-GENOME    AATTTCTCCGTCTTTCTGGAGATGAATGGTATATGCTGTTTCAGTTATTATCTACAGTGT
BIG-ORF       AATTTCTCCGTCTTTCTGGAGATGAATG--------------------------------
BIG-TAIR      AATTTCTCCGTCTTTCTGGAGATGAATG--------------------------------
              ****************************
BIG-GENOME    GTCTATTTGCATGTGTAGTTCAAAATTCAAACACAATTTTGTTTATTATCCTTGTATTAT
BIG-ORF       ------------------------------------------------------------
BIG-TAIR      ------------------------------------------------------------

BIG-GENOME    GTAATAGGAGCGGCTGTTTGTCATACAAATATAATCTTCCTTTCATATAGTTTTGTTTAT
BIG-ORF       ------------------------------------------------------------
BIG-TAIR      ------------------------------------------------------------

BIG-GENOME    ATGAGATATGTTTGTTATGTAGGCTCTGAAGTGGTTGATAATCTGATGCAAGTGCTATGG
BIG-ORF       ----------------------GCTCTGAAGTGGTTGATAATCTGATGCAAGTGCTATGG
BIG-TAIR      ----------------------GCTCTGAAGTGGTTGATAATCTGATGCAAGTGCTATGG
                                    **************************************
BIG-GENOME    GGAATTTTGGAGTCAGAGCCACTCGACACACCTACTATGAACAATGTCGTGATGTCCTCC
BIG-ORF       GGAATTTTGGAGTCAGAGCCACTCGACACACCTACTATGAACAATGTCGTGATGTCCTCC
BIG-TAIR      GGAATTTTGGAGTCAGAGCCACTCGACACACCTACTATGAACAATGTCGTGATGTCCTCC
              ************************************************************
BIG-GENOME    GTTGAACTAATCTATAGCTATGCAGAGTGTTTGGCATCTCAAGGAAAGGATACAGGGGTT
BIG-ORF       GTTGAACTAATCTATAGCTATGCAGAGTGTTTGGCATCTCAAGGAAAGGATACAGGGGTT
BIG-TAIR      GTTGAACTAATCTATAGCTATGCAGAGTGTTTGGCATCTCAAGGAAAGGATACAGGGGTT
              ************************************************************
BIG-GENOME    CATTCTGTAGCTCCTGCAGTTCAGTTACTGAAAGCACTTATGTTGTTTCCCAATGAGTCT
BIG-ORF       CATTCTGTAGCTCCTGCAGTTCAGTTACTGAAAGCACTTATGTTGTTTCCCAATGAGTCT
BIG-TAIR      CATTCTGTAGCTCCTGCAGTTCAGTTACTGAAAGCACTTATGTTGTTTCCCAATGAGTCT
              ************************************************************
BIG-GENOME    GTGCAAACATCCAGCAGGTGTGTTCTTGTGTATGTTTTTTATACTCTGCTCTCTTAGATA
BIG-ORF       GTGCAAACATCCAGCAG-------------------------------------------
```

```
BIG-TAIR      GTGCAAACATCCAGCAGGTGTGTTCTTGT-------------------------------
              *****************
BIG-GENOME    TGTACTAGTCTTCTAACTCAGTTTCTTTACAGCCTAGCTATATCATCAAGGTTACTTCAG
BIG-ORF       --------------------------------CCTAGCTATATCATCAAGGTTACTTCAG
BIG-TAIR      --------------------------------CCTAGCTATATCATCAAGGTTACTTCAG
                                              ****************************
BIG-GENOME    GTTCCTTTCCCAAAGCAAACAATGTTGACAACAGATGATTTGGTTGACAATGTTACAACT
BIG-ORF       GTTCCTTTCCCAAAGCAAACAATGTTGACAACAGATGATTTGGTTGACAATGTTACAACT
BIG-TAIR      GTTCCTTTCCCAAAGCAAACAATGTTGACAACAGATGATTTGGTTGACAATGTTACAACT
              ************************************************************
BIG-GENOME    CCTTCGGTGCCTATCAGAACAGCTGGTGGAAATACACATGTCATGATTGAGGAGGACTCT
BIG-ORF       CCTTCGGTGCCTATCAGAACAGCTGGTGGAAATACACATGTCATGATTGAGGAGGACTCT
BIG-TAIR      CCTTCGGTGCCTATCAGAACAGCTGGTGGAAATACACATGTCATGATTGAGGAGGACTCT
              ************************************************************
BIG-GENOME    ATAACTTCATCTGTTCAATACTGCTGTGATGGCTGCTCCACCGTCCCTATACTGAGAAGA
BIG-ORF       ATAACTTCATCTGTTCAATACTGCTGTGATGGCTGCTCCACCGTCCCTATACTGAGAAGA
BIG-TAIR      ATAACTTCATCTGTTCAATACTGCTGTGATGGCTGCTCCACCGTCCCTATACTGAGAAGA
              ************************************************************
BIG-GENOME    AGGTGGCACTGCACTGTTTGTCCAGATTTTGATCTATGTGAGGCATGCTATGAAGTGTTA
BIG-ORF       AGGTGGCACTGCACTGTTTGTCCAGATTTTGATCTATGTGAGGCATGCTATGAAGTGTTA
BIG-TAIR      AGGTGGCACTGCACTGTTTGTCCAGATTTTGATCTATGTGAGGCATGCTATGAAGTGTTA
              ************************************************************
BIG-GENOME    GATGCAGATCGTCTACCACCACCACACACTCGAGATCATCCTATGACAGCAATTCCAATA
BIG-ORF       GATGCAGATCGTCTACCACCACCACACACTCGAGATCATCCTATGACAGCAATTCCAATA
BIG-TAIR      GATGCAGATCGTCTACCACCACCACACACTCGAGATCATCCTATGACAGCAATTCCAATA
              ************************************************************
BIG-GENOME    GAAGTGGAATCACTTGGTGCAGACACCAATGAGATTCAGTTCTCTGCGGATGAAGTAGGT
BIG-ORF       GAAGTGGAATCACTTGGTGCAGACACCAATGAGATTCAGTTCTCTGCGGATGAAGTAGGT
BIG-TAIR      GAAGTGGAATCACTTGGTGCAGACACCAATGAGATTCAGTTCTCTGCGGATGAAGTAGGT
              ************************************************************
BIG-GENOME    ATTTCAAATATGTTGCCGGTGGTAACCAGTAGCATTCCACAAGCTTCAACTCCCTCCATC
BIG-ORF       ATTTCAAATATGTTGCCGGTGGTAACCAGTAGCATTCCACAAGCTTCAACTCCCTCCATC
BIG-TAIR      ATTTCAAATATGTTGCCGGTGGTAACCAGTAGCATTCCACAAGCTTCAACTCCCTCCATC
              ************************************************************
BIG-GENOME    CATGTCTTGGAACCTGGTGAATCTGCTGAGTTCTCTGCCTCACTGACCGATCCTATTTCT
BIG-ORF       CATGTCTTGGAACCTGGTGAATCTGCTGAGTTCTCTGCCTCACTGACCGATCCTATTTCT
BIG-TAIR      CATGTCTTGGAACCTGGTGAATCTGCTGAGTTCTCTGCCTCACTGACCGATCCTATTTCT
              ************************************************************
BIG-GENOME    ATCTCAGCGTCAAAGCGTGCCGTAAATTCTTTGATTCTCTCTGAGTTTCTCCAAGAGTTG
BIG-ORF       ATCTCAGCGTCAAAGCGTGCCGTAAATTCTTTGATTCTCTCTGAGTTTCTCCAAGAGTTG
BIG-TAIR      ATCTCAGCGTCAAAGCGTGCCGTAAATTCTTTGATTCTCTCTGAGTTTCTCCAAGAGTTG
              ************************************************************
BIG-GENOME    AGTGGATGGATGGAAACAGTATCCGGTGTTCAAGCTATTCCTGTGATGCAATTGTTCTAC
BIG-ORF       AGTGGATGGATGGAAACAGTATCCGGTGTTCAAGCTATTCCTGTGATGCAATTGTTCTAC
```

BIG-TAIR AGTGGATGGATGGAAACAGTATCCGGTGTTCAAGCTATTCCTGTGATGCAATTGTTCTAC

****

BIG-GENOME AGATTATCATCTGCCATTGGTGGAGCCTTTATGGATAGCTCAAAGCCCGAAGAAATAAGC

BIG-ORF AGATTATCATCTGCCATTGGTGGAGCCTTTATGGATAGCTCAAAGCCCGAAGAAATAAGC

BIG-TAIR AGATTATCATCTGCCATTGGTGGAGCCTTTATGGATAGCTCAAAGCCCGAAGAAATAAGC

****

BIG-GENOME TTGGATAAGTTAATTAAATGGCTTTTGGGTGAAATCAATCTCAGCAAGCCGTTTGCTGCT

BIG-ORF TTGGATAAGTTAATTAAATGGCTTTTGGGTGAAATCAATCTCAGCAAGCCGTTTGCTGCT

BIG-TAIR TTGGATAAGTTAATTAAATGGCTTTTGGGTGAAATCAATCTCAGCAAGCCGTTTGCTGCT

****

BIG-GENOME TCAACTCGCTCTTCCTTAGGGGAAATTGTGATTCTTGTGTTTATGTTTTTCACCTTGATG

BIG-ORF TCAACTCGCTCTTCCTTAGGGGAAATTGTGATTCTTGTGTTTATGTTTTTCACCTTGATG

BIG-TAIR TCAACTCGCTCTTCCTTAGGGGAAATTGTGATTCTTGTGTTTATGTTTTTCACCTTGATG

****

BIG-GENOME CTTCGGAGTTGGCACCAGCCTGGTAGTGATGGTAGTAGCTCCAAATTAGGTGGAAGTACA

BIG-ORF CTTCGGAGTTGGCACCAGCCTGGTAGTGATGGTAGTAGCTCCAAATTAGGTGGAAGTACA

BIG-TAIR CTTCGGAGTTGGCACCAGCCTGGTAGTGATGGTAGTAGCTCCAAATTAGGTGGAAGTACA

****

BIG-GENOME GATGTACATGACCGGAGGATTGTCCAGAGCTCCACTGTAGTAGCTACTCAGTCTTCTTTA

BIG-ORF GATGTACATGACCGGAGGATTGTCCAGAGCTCCACTGTAGTAGCTACTCAGTCTTCTTTA

BIG-TAIR GATGTACATGACCGGAGGATTGTCCAGAGCTCCACTGTAGTAGCTACTCAGTCTTCTTTA

****

BIG-GENOME CATGTTCAAGAGAGAGATGACTTTGCATCTCAGCTAGTTCGTGCGTGCAGTTGCCTCAGG

BIG-ORF CATGTTCAAGAGAGAGATGACTTTGCATCTCAGCTAGTTCGTGCGTGCAGTTGCCTCAGG

BIG-TAIR CATGTTCAAGAGAGAGATGACTTTGCATCTCAGCTAGTTCGTGCGTGCAGTTGCCTCAGG

****

BIG-GENOME AACCAAGAGTTTGTTAATTATCTGATGAACATACTTCAACAGCTAGTGCATGTTTTTAAG

BIG-ORF AACCAAGAGTTTGTTAATTATCTGATGAACATACTTCAACAGCTAGTGCATGTTTTTAAG

BIG-TAIR AACCAAGAGTTTGTTAATTATCTGATGAACATACTTCAACAGCTAGTGCATGTTTTTAAG

****

BIG-GENOME TCACGTGCTGCCAATGTTGAAGCTCGTGGATCAAGCTCTGGTTCTGGTTGTGGAGCCATG

BIG-ORF TCACGTGCTGCCAATGTTGAAGCTCGTGGATCAAGCTCTGGTTCTGGTTGTGGAGCCATG

BIG-TAIR TCACGTGCTGCCAATGTTGAAGCTCGTGGATCAAGCTCTGGTTCTGGTTGTGGAGCCATG

****

BIG-GENOME CTCACAGTCAGAAGAGATTTACCTGCGGGTAACTATTCTCCCTTCTTTTCGGATTCATAT

BIG-ORF CTCACAGTCAGAAGAGATTTACCTGCGGGTAACTATTCTCCCTTCTTTTCGGATTCATAT

BIG-TAIR CTCACAGTCAGAAGAGATTTACCTGCGGGTAACTATTCTCCCTTCTTTTCGGATTCATAT

****

BIG-GENOME GCAAAAGCCCATAGAGCAGATATTTTTGTGGACTATCACAGGCTGCTACTTGAGAATGTG

BIG-ORF GCAAAAGCCCATAGAGCAGATATTTTTGTGGACTATCACAGGCTGCTACTTGAGAATGTG

BIG-TAIR GCAAAAGCCCATAGAGCAGATATTTTTGTGGACTATCACAGGCTGCTACTTGAGAATGTG

****

BIG-GENOME TTTCGCTTAGTATATACCTTAGTTAGACCTGAAAAACAAGAGAAAATGGGGGAAAAGGAA

BIG-ORF TTTCGCTTAGTATATACCTTAGTTAGACCTGAAAAACAAGAGAAAATGGGGGAAAAGGAA

```
BIG-TAIR      TTTCGCTTAGTATATACCTTAGTTAGACCTGAAAAACAAGAGAAAATGGGGGAAAAGGAA
              ************************************************************
BIG-GENOME    AAGGTATACAGGAATGCTTCTTCTAAAGATTTAAAGCTGGACGGTTTCCAGGATGTTCTA
BIG-ORF       AAGGTATACAGGAATGCTTCTTCTAAAGATTTAAAGCTGGACGGTTTCCAGGATGTTCTA
BIG-TAIR      AAGGTATACAGGAATGCTTCTTCTAAAGATTTAAAGCTGGACGGTTTCCAGGATGTTCTA
              ************************************************************
BIG-GENOME    TGCAGCTATATAAATAACCCTCATACGGCTTTTGTTAGGAGATATGCTAGAAGGCTGTTT
BIG-ORF       TGCAGCTATATAAATAACCCTCATACGGCTTTTGTTAGGAGATATGCTAGAAGGCTGTTT
BIG-TAIR      TGCAGCTATATAAATAACCCTCATACGGCTTTTGTTAGGAGATATGCTAGAAGGCTGTTT
              ************************************************************
BIG-GENOME    TTGCATCTTTGCGGAAGCAAAACTCAATACTACAGTGTCAGAGATTCATGGCAGTTTTCC
BIG-ORF       TTGCATCTTTGCGGAAGCAAAACTCAATACTACAGTGTCAGAGATTCATGGCAGTTTTCC
BIG-TAIR      TTGCATCTTTGCGGAAGCAAAACTCAATACTACAGTGTCAGAGATTCATGGCAGTTTTCC
              ************************************************************
BIG-GENOME    AATGAAGTGAAAAACCTATACAAGCATGTTGAAAAGTCTGGTGGTTTTGAAAACAATGTT
BIG-ORF       AATGAAGTGAAAAACCTATACAAGCATGTTGAAAAGTCTGGTGGTTTTGAAAACAATGTT
BIG-TAIR      AATGAAGTGAAAAACCTATACAAGCATGTTGAAAAGTCTGGTGGTTTTGAAAACAATGTT
              ************************************************************
BIG-GENOME    TCATATGAAAGAAGTGTAAAGATTGTGAAGTCGCTTTCTACAATTGCTGAAGTTGCTGTG
BIG-ORF       TCATATGAAAGAAGTGTAAAGATTGTGAAGTCGCTTTCTACAATTGCTGAAGTTGCTGTG
BIG-TAIR      TCATATGAAAGAAGTGTAAAGATTGTGAAGTCGCTTTCTACAATTGCTGAAGTTGCTGTG
              ************************************************************
BIG-GENOME    GCAAGACCTCGGAACTGGCAGAAGTACTGTCTCAGGCATGGAGATTTTCTGTCCTTCCTA
BIG-ORF       GCAAGACCTCGGAACTGGCAGAAGTACTGTCTCAGGCATGGAGATTTTCTGTCCTTCCTA
BIG-TAIR      GCAAGACCTCGGAACTGGCAGAAGTACTGTCTCAGGCATGGAGATTTTCTGTCCTTCCTA
              ************************************************************
BIG-GENOME    CTGAATGGTGTATTCCATTTTGCGGAAGAGTCTGTAATCCAGACACTTAAACTCCTCAAT
BIG-ORF       CTGAATGGTGTATTCCATTTTGCGGAAGAGTCTGTAATCCAGACACTTAAACTCCTCAAT
BIG-TAIR      CTGAATGGTGTATTCCATTTTGCGGAAGAGTCTGTAATCCAGACACTTAAACTCCTCAAT
              ************************************************************
BIG-GENOME    TTGGCCTTTTATCAAGGAAAAGATGTGAGCAGCTCAGTGCAGAAAGCTGAAGCAACTGAA
BIG-ORF       TTGGCCTTTTATCAAGGAAAAGATGTGAGCAGCTCAGTGCAGAAAGCTGAAGCAACTGAA
BIG-TAIR      TTGGCCTTTTATCAAGGAAAAGATGTGAGCAGCTCAGTGCAGAAAGCTGAAGCAACTGAA
              ************************************************************
BIG-GENOME    GTAGTGACAGGCTCAAACCGGTCAGGATCTCAGTCAGTGGATTCAAAAAAGAAGAAAAAA
BIG-ORF       GTAGTGACAGGCTCAAACCGGTCAGGATCTCAGTCAGTGGATTCAAAAAAGAAGAAAAAA
BIG-TAIR      GTAGTGACAGGCTCAAACCGGTCAGGATCTCAGTCAGTGGATTCAAAAAAGAAGAAAAAA
              ************************************************************
BIG-GENOME    GGTGAAGATGGGCATGATTCGGGTTTGGAAAAATTATATGTGGATATGGAAGGGGTGGTC
BIG-ORF       GGTGAAGATGGGCATGATTCGGGTTTGGAAAAATTATATGTGGATATGGAAGGGGTGGTC
BIG-TAIR      GGTGAAGATGGGCATGATTCGGGTTTGGAAAAATTATATGTGGATATGGAAGGGGTGGTC
              ************************************************************
BIG-GENOME    GATATCTTCAGTGCCAATTGCGGAGATCTCTTGAGGCAGTTTATTGATTTCTTTCTGCTG
BIG-ORF       GATATCTTCAGTGCCAATTGCGGAGATCTCTTGAGGCAGTTTATTGATTTCTTTCTGCTG
```

```
BIG-TAIR      GATATCTTCAGTGCCAATTGCGGAGATCTCTTGAGGCAGTTTATTGATTTCTTTCTGCTG
              ************************************************************
BIG-GENOME    GAATGGAATTCGAGCTCTGTTCGCACTGAAGCAAAATCTGTTATTTATGGCTTGTGGCAT
BIG-ORF       GAATGGAATTCGAGCTCTGTTCGCACTGAAGCAAAATCTGTTATTTATGGCTTGTGGCAT
BIG-TAIR      GAATGGAATTCGAGCTCTGTTCGCACTGAAGCAAAATCTGTTATTTATGGCTTGTGGCAT
              ************************************************************
BIG-GENOME    CATGGAAGACACTCGTTCAAAGAAAGTCTGTTAGCAGCTCTTTTGCAGAAGGTTAGATAC
BIG-ORF       CATGGAAGACACTCGTTCAAAGAAAGTCTGTTAGCAGCTCTTTTGCAGAAGGTTAGATAC
BIG-TAIR      CATGGAAGACACTCGTTCAAAGAAAGTCTGTTAGCAGCTCTTTTGCAGAAGGTTAGATAC
              ************************************************************
BIG-GENOME    CTGCCAGCATATGGTCAGAATATTGTTGAGTACACAGAACTTGTCTCCTTGTTGCTTGAC
BIG-ORF       CTGCCAGCATATGGTCAGAATATTGTTGAGTACACAGAACTTGTCTCCTTGTTGCTTGAC
BIG-TAIR      CTGCCAGCATATGGTCAGAATATTGTTGAGTACACAGAACTTGTCTCCTTGTTGCTTGAC
              ************************************************************
BIG-GENOME    AAGGCTCCAGAAAACAACTCAAAGCAAGCAATTAATGAGCTTGTGGATCGGTGCCTGAAC
BIG-ORF       AAGGCTCCAGAAAACAACTCAAAGCAAGCAATTAATGAGCTTGTGGATCGGTGCCTGAAC
BIG-TAIR      AAGGCTCCAGAAAACAACTCAAAGCAAGCAATTAATGAGCTTGTGGATCGGTGCCTGAAC
              ************************************************************
BIG-GENOME    CCTGATGTGATAAGGTGCTTTTTTGAGACGCTGCATTCCCAGAACGAACTCATTGCCAAT
BIG-ORF       CCTGATGTGATAAGGTGCTTTTTTGAGACGCTGCATTCCCAGAACGAACTCATTGCCAAT
BIG-TAIR      CCTGATGTGATAAGGTGCTTTTTTGAGACGCTGCATTCCCAGAACGAACTCATTGCCAAT
              ************************************************************
BIG-GENOME    CATCCAAACTCCCGTATATACAGCACTCTGGGTAATCTTGTGGAATTTGATGGTTATTAT
BIG-ORF       CATCCAAACTCCCGTATATACAGCACTCTGGGTAATCTTGTGGAATTTGATGGTTATTAT
BIG-TAIR      CATCCAAACTCCCGTATATACAGCACTCTGGGTAATCTTGTGGAATTTGATGGTTATTAT
              ************************************************************
BIG-GENOME    CTTGAGAGCGAACCTTGTGTTGCCTGCAGTTCTCCTGATGTGCCATATAGCAAAATGAAG
BIG-ORF       CTTGAGAGCGAACCTTGTGTTGCCTGCAGTTCTCCTGATGTGCCATATAGCAAAATGAAG
BIG-TAIR      CTTGAGAGCGAACCTTGTGTTGCCTGCAGTTCTCCTGATGTGCCATATAGCAAAATGAAG
              ************************************************************
BIG-GENOME    CTTGAAAGTTTGAAATCCGAGACAAAGTTTACTGATAACCGCATCATTGTGAAATGTACT
BIG-ORF       CTTGAAAGTTTGAAATCCGAGACAAAGTTTACTGATAACCGCATCATTGTGAAATGTACT
BIG-TAIR      CTTGAAAGTTTGAAATCCGAGACAAAGTTTACTGATAACCGCATCATTGTGAAATGTACT
              ************************************************************
BIG-GENOME    GGGAGTTATACGATACAGTCTGTGACAATGAATGTTCATGATGCACGAAAGTCAAAGTCG
BIG-ORF       GGGAGTTATACGATACAGTCTGTGACAATGAATGTTCATGATGCACGAAAGTCAAAGTCG
BIG-TAIR      GGGAGTTATACGATACAGTCTGTGACAATGAATGTTCATGATGCACGAAAGTCAAAGTCG
              ************************************************************
BIG-GENOME    GTGAAGGTCCTAAACTTGTATTACAACAATCGCCCTGTCTCGGATTTATCAGAGCTTAAA
BIG-ORF       GTGAAGGTCCTAAACTTGTATTACAACAATCGCCCTGTCTCGGATTTATCAGAGCTTAAA
BIG-TAIR      GTGAAGGTCCTAAACTTGTATTACAACAATCGCCCTGTCTCGGATTTATCAGAGCTTAAA
              ************************************************************
BIG-GENOME    AACAACTGGTCACTGTGGAAGCGTGCCAAAAGTTGTCATCTGTCTTTTAACCAAACTGAA
BIG-ORF       AACAACTGGTCACTGTGGAAGCGTGCCAAAAGTTGTCATCTGTCTTTTAACCAAACTGAA
```

```
BIG-TAIR        AACAACTGGTCACTGTGGAAGCGTGCCAAAAGTTGTCATCTGTCTTTTAACCAAACTGAA
                ************************************************************
BIG-GENOME      CTGAAGGTGGAATTTCCTATTCCTATTACTGCTTGTAACTTCATGATCGAGTTGGATTCT
BIG-ORF         CTGAAGGTGGAATTTCCTATTCCTATTACTGCTTGTAACTTCATGATCGAGTTGGATTCT
BIG-TAIR        CTGAAGGTGGAATTTCCTATTCCTATTACTGCTTGTAACTTCATGATCGAGTTGGATTCT
                ************************************************************
BIG-GENOME      TTCTATGAGAATCTTCAGGCGTTATCTCTTGAACCATTGCAATGTCCTCGATGCAGCCGA
BIG-ORF         TTCTATGAGAATCTTCAGGCGTTATCTCTTGAACCATTGCAATGTCCTCGATGCAGCCGA
BIG-TAIR        TTCTATGAGAATCTTCAGGCGTTATCTCTTGAACCATTGCAATGTCCTCGATGCAGCCGA
                ************************************************************
BIG-GENOME      CCTGTCACTGATAAACATGGTATTTGCAGCAACTGCCATGAGAATGCTTATCAGTGCAGG
BIG-ORF         CCTGTCACTGATAAACATGGTATTTGCAGCAACTGCCATGAGAATGCTTATCAGTGCAGG
BIG-TAIR        CCTGTCACTGATAAACATGGTATTTGCAGCAACTGCCATGAGAATGCTTATCAGTGCAGG
                ************************************************************
BIG-GENOME      CAATGCCGTAACATTAATTATGAAAATTTGGATTCATTCCTTTGCAACGAGTGTGGCTAC
BIG-ORF         CAATGCCGTAACATTAATTATGAAAATTTGGATTCATTCCTTTGCAACGAGTGTGGCTAC
BIG-TAIR        CAATGCCGTAACATTAATTATGAAAATTTGGATTCATTCCTTTGCAACGAGTGTGGCTAC
                ************************************************************
BIG-GENOME      AGCAAGTATGGGAGGTTTGAGTTTAACTTCATGGCAAAACCAAGCTTCATATTTGATAAT
BIG-ORF         AGCAAGTATGGGAGGTTTGAGTTTAACTTCATGGCAAAACCAAGCTTCATATTTGATAAT
BIG-TAIR        AGCAAGTATGGGAGGTTTGAGTTTAACTTCATGGCAAAACCAAGCTTCATATTTGATAAT
                ************************************************************
BIG-GENOME      ATGGAAAATGACGAAGATATGAAGAAGGGATTGGCTGCTATAGAGTCTGAGTCTGAAAAC
BIG-ORF         ATGGAAAATGACGAAGATATGAAGAAGGGATTGGCTGCTATAGAGTCTGAGTCTGAAAAC
BIG-TAIR        ATGGAAAATGACGAAGATATGAAGAAGGGATTGGCTGCTATAGAGTCTGAGTCTGAAAAC
                ************************************************************
BIG-GENOME      GCTCACAAGAGATATCAGCAACTACTGGGATTCAAGAAGCCGCTTCTGAAGATAGTTTCA
BIG-ORF         GCTCACAAGAGATATCAGCAACTACTGGGATTCAAGAAGCCGCTTCTGAAGATAGTTTCA
BIG-TAIR        GCTCACAAGAGATATCAGCAACTACTGGGATTCAAGAAGCCGCTTCTGAAGATAGTTTCA
                ************************************************************
BIG-GENOME      AGCATTGGTGAAACTGAAATGGATTCGCAGCACAAGGACACTGTCCAGCAAATGATGGCA
BIG-ORF         AGCATTGGTGAAACTGAAATGGATTCGCAGCACAAGGACACTGTCCAGCAAATGATGGCA
BIG-TAIR        AGCATTGGTGAAACTGAAATGGATTCGCAGCACAAGGACACTGTCCAGCAAATGATGGCA
                ************************************************************
BIG-GENOME      TCTTTACCTGGTCCATCATGCAAGATAAACCGCAAAATCGCACTTTTGGGCGTACTGTAT
BIG-ORF         TCTTTACCTGGTCCATCATGCAAGATAAACCGCAAAATCGCACTTTTGGGCGTACTGTAT
BIG-TAIR        TCTTTACCTGGTCCATCATGCAAGATAAACCGCAAAATCGCACTTTTGGGCGTACTGTAT
                ************************************************************
BIG-GENOME      GGTGAGAAGTGCAAAGCTGCTTTTGATTCTGTCAGCAAAAGTGTACAAACACTGCAGGGG
BIG-ORF         GGTGAGAAGTGCAAAGCTGCTTTTGATTCTGTCAGCAAAAGTGTACAAACACTGCAGGGG
BIG-TAIR        GGTGAGAAGTGCAAAGCTGCTTTTGATTCTGTCAGCAAAAGTGTACAAACACTGCAGGGG
                ************************************************************
BIG-GENOME      CTTCGTAGAGTTTTGATGAGCTACCTCCATCAGAAAAATTCAAATTTTTCATCAGGTGCC
BIG-ORF         CTTCGTAGAGTTTTGATGAGCTACCTCCATCAGAAAAATTCAAATTTTTCATCAGGTGCC
```

```
BIG-TAIR      CTTCGTAGAGTTTTGATGAGCTACCTCCATCAGAAAAATTCAAATTTTTCATCAGGTGCC
              ************************************************************
BIG-GENOME    TCAAGATGTGTGGTCTCAAAGACCCCAAACAATTGTTATGGTTGTGCAACTACATTTGTC
BIG-ORF       TCAAGATGTGTGGTCTCAAAGACCCCAAACAATTGTTATGGTTGTGCAACTACATTTGTC
BIG-TAIR      TCAAGATGTGTGGTCTCAAAGACCCCAAACAATTGTTATGGTTGTGCAACTACATTTGTC
              ************************************************************
BIG-GENOME    ACTCAATGTCTTGAGATTCTTCAAGTGCTGTCGAAGCATCCAAGATCTAGAAAACAACTT
BIG-ORF       ACTCAATGTCTTGAGATTCTTCAAGTGCTGTCGAAGCATCCAAGATCTAGAAAACAACTT
BIG-TAIR      ACTCAATGTCTTGAGATTCTTCAAGTGCTGTCGAAGCATCCAAGATCTAGAAAACAACTT
              ************************************************************
BIG-GENOME    GTTGCAGCTGGTATTTTGTCTGAGTTGTTTGAAAACAATATTCACCAAGGTCCGAAAACA
BIG-ORF       GTTGCAGCTGGTATTTTGTCTGAGTTGTTTGAAAACAATATTCACCAAGGTCCGAAAACA
BIG-TAIR      GTTGCAGCTGGTATTTTGTCTGAGTTGTTTGAAAACAATATTCACCAAGGTCCGAAAACA
              ************************************************************
BIG-GENOME    GCCCGTGCTCAAGCTAGAGCAGCTCTTTCTACTTTCTCGGAGGGTGATCTGAGTGCAGTG
BIG-ORF       GCCCGTGCTCAAGCTAGAGCAGCTCTTTCTACTTTCTCGGAGGGTGATCTGAGTGCAGTG
BIG-TAIR      GCCCGTGCTCAAGCTAGAGCAGCTCTTTCTACTTTCTCGGAGGGTGATCTGAGTGCAGTG
              ************************************************************
BIG-GENOME    AATGAGTTAAACAATCTGGTACAGAAGAAAATAATGTACTGCCTTGAACACCATCGTTCC
BIG-ORF       AATGAGTTAAACAATCTGGTACAGAAGAAAATAATGTACTGCCTTGAACACCATCGTTCC
BIG-TAIR      AATGAGTTAAACAATCTGGTACAGAAGAAAATAATGTACTGCCTTGAACACCATCGTTCC
              ************************************************************
BIG-GENOME    ATGGATATTGCGCTCGCCACTCGTGAGGAAATGTTGTTGCTTTCAGAAGTTTGCTCTCTC
BIG-ORF       ATGGATATTGCGCTCGCCACTCGTGAGGAAATGTTGTTGCTTTCAGAAGTTTGCTCTCTC
BIG-TAIR      ATGGATATTGCGCTCGCCACTCGTGAGGAAATGTTGTTGCTTTCAGAAGTTTGCTCTCTC
              ************************************************************
BIG-GENOME    ACTGATGAATTCTGGGAGTCAAGACTGCGCCTTGTTTTTCAGCTACTATTTTCATCCATT
BIG-ORF       ACTGATGAATTCTGGGAGTCAAGACTGCGCCTTGTTTTTCAGCTACTATTTTCATCCATT
BIG-TAIR      ACTGATGAATTCTGGGAGTCAAGACTGCGCCTTGTTTTTCAGCTACTATTTTCATCCATT
              ************************************************************
BIG-GENOME    AAATTGGGTGCTAAACATCCTGCTATATCAGAGCACATAATTCTTCCTTGCCTTAAGATT
BIG-ORF       AAATTGGGTGCTAAACATCCTGCTATATCAGAGCACATAATTCTTCCTTGCCTTAAGATT
BIG-TAIR      AAATTGGGTGCTAAACATCCTGCTATATCAGAGCACATAATTCTTCCTTGCCTTAAGATT
              ************************************************************
BIG-GENOME    ATTTCTGTGGCATGTACACCTCCAAAACCAGATACGGCAGAGAAAGAGCAAACGATGGGA
BIG-ORF       ATTTCTGTGGCATGTACACCTCCAAAACCAGATACGGCAGAGAAAGAGCAAACGATGGGA
BIG-TAIR      ATTTCTGTGGCATGTACACCTCCAAAACCAGATACGGCAGAGAAAGAGCAAACGATGGGA
              ************************************************************
BIG-GENOME    AAATCTGCTCCTGCAGTGCAAGAAAAGGATGAGAATGCTGCTGGCGTCATAAAGTATTCA
BIG-ORF       AAATCTGCTCCTGCAGTGCAAGAAAAGGATGAGAATGCTGCTGGCGTCATAAAGTATTCA
BIG-TAIR      AAATCTGCTCCTGCAGTGCAAGAAAAGGATGAGAATGCTGCTGGCGTCATAAAGTATTCA
              ************************************************************
BIG-GENOME    TCAGAAAGCGAAGAAAATAACCTGAATGTATCTCAGAAGACGCGAGATATTCAGCTTGTG
BIG-ORF       TCAGAAAGCGAAGAAAATAACCTGAATGTATCTCAGAAGACGCGAGATATTCAGCTTGTG
```

BIG-TAIR TCAGAAAGCGAAGAAAATAACCTGAATGTATCTCAGAAGACGCGAGATATTCAGCTTGTG

****

BIG-GENOME AGTTATTTGGAATGGGAGAAAGGGGCATCATATCTCGACTTTGTGAGGAGGCAATACAAA

BIG-ORF AGTTATTTGGAATGGGAGAAAGGGGCATCATATCTCGACTTTGTGAGGAGGCAATACAAA

BIG-TAIR AGTTATTTGGAATGGGAGAAAGGGGCATCATATCTCGACTTTGTGAGGAGGCAATACAAA

****

BIG-GENOME GCCTCTCAGTCAATTAGAGGTGCGAGCCAAAAGTCTAGAACCCACAGATCAGATTTCTTG

BIG-ORF GCCTCTCAGTCAATTAGAGGTGCGAGCCAAAAGTCTAGAACCCACAGATCAGATTTCTTG

BIG-TAIR GCCTCTCAGTCAATTAGAGGTGCGAGCCAAAAGTCTAGAACCCACAGATCAGATTTCTTG

****

BIG-GENOME GCACTTAAATATACACTTAGGTGGAAGCGACGGTCTAGCAGGACATCTAAAGGTGGTTTA

BIG-ORF GCACTTAAATATACACTTAGGTGGAAGCGACGGTCTAGCAGGACATCTAAAGGTGGTTTA

BIG-TAIR GCACTTAAATATACACTTAGGTGGAAGCGACGGTCTAGCAGGACATCTAAAGGTGGTTTA

****

BIG-GENOME CAGGCATTTGAGCTCGGGTCGTGGGTCACAGAGCTTATTCTTAGTGCATGTTCTCAATCT

BIG-ORF CAGGCATTTGAGCTCGGGTCGTGGGTCACAGAGCTTATTCTTAGTGCATGTTCTCAATCT

BIG-TAIR CAGGCATTTGAGCTCGGGTCGTGGGTCACAGAGCTTATTCTTAGTGCATGTTCTCAATCT

****

BIG-GENOME ATCAGGTCGGAGATGTGCACATTGATTAGTTTACTCGCTGCCCAAAGCTCACCTAGGCGC

BIG-ORF ATCAGGTCGGAGATGTGCACATTGATTAGTTTACTCGCTGCCCAAAGCTCACCTAGGCGC

BIG-TAIR ATCAGGTCGGAGATGTGCACATTGATTAGTTTACTCGCTGCCCAAAGCTCACCTAGGCGC

****

BIG-GENOME TACAGGCTGATTAATTTGCTGATAGGCTTGCTTCCCGCTACGCTTGCAGCGGGTGAAAGT

BIG-ORF TACAGGCTGATTAATTTGCTGATAGGCTTGCTTCCCGCTACGCTTGCAGCGGGTGAAAGT

BIG-TAIR TACAGGCTGATTAATTTGCTGATAGGCTTGCTTCCCGCTACGCTTGCAGCGGGTGAAAGT

****

BIG-GENOME AGCGCAGAATACTTTGAGCTACTCTTCAAAATGATTGAGACACAAGATGCACTTCTTTTC

BIG-ORF AGCGCAGAATACTTTGAGCTACTCTTCAAAATGATTGAGACACAAGATGCACTTCTTTTC

BIG-TAIR AGCGCAGAATACTTTGAGCTACTCTTCAAAATGATTGAGACACAAGATGCACTTCTTTTC

****

BIG-GENOME TTAACTGTCCGGGGTTGTCTGACAACAATCTGTAAATTGATCTCCCAAGAGGTGGGAAAC

BIG-ORF TTAACTGTCCGGGGTTGTCTGACAACAATCTGTAAATTGATCTCCCAAGAGGTGGGAAAC

BIG-TAIR TTAACTGTCCGGGGTTGTCTGACAACAATCTGTAAATTGATCTCCCAAGAGGTGGGAAAC

****

BIG-GENOME ATTGAATCTCTAGAAAGGAGTCTTCAGATTGACATTTCACAGGGATTTACCCTCCACAAG

BIG-ORF ATTGAATCTCTAGAAAGGAGTCTTCAGATTGACATTTCACAGGGATTTACCCTCCACAAG

BIG-TAIR ATTGAATCTCTAGAAAGGAGTCTTCAGATTGACATTTCACAGGGATTTACCCTCCACAAG

****

BIG-GENOME CTCTTAGAACTCCTTGGAAAGTTCTTAGAGGTTCCAAATATTCGTTCCAGGTAATTTTCT

BIG-ORF CTCTTAGAACTCCTTGGAAAGTTCTTAGAGGTTCCAAATATTCGTTCCAG----------

BIG-TAIR CTCTTAGAACTCCTTGGAAAGTTCTTAGAGGTTCCAAATATTCGTTCCAG----------

****

BIG-GENOME CATCATTAAATCTATTGATCTGCCATTGACTTCTTCATACCTGTCTAATGTTTTGAGTGT

BIG-ORF --

```
BIG-TAIR        ------------------------------------------------------------

BIG-GENOME      GGCAGATTCATGAGAGACAATTTACTGTCTCATGTTCTGGAGGCCCTCATTGTAATTCGT
BIG-ORF         -----ATTCATGAGAGACAATTTACTGTCTCATGTTCTGGAGGCCCTCATTGTAATTCGT
BIG-TAIR        -----ATTCATGAGAGACAATTTACTGTCTCATGTTCTGGAGGCCCTCATTGTAATTCGT
                     *******************************************************
BIG-GENOME      GGCTTGATAGTGCAAAAGACAAAGCTTATAAATGATTGCAACAGACGCCTAAAAGATCTT
BIG-ORF         GGCTTGATAGTGCAAAAGACAAAGCTTATAAATGATTGCAACAGACGCCTAAAAGATCTT
BIG-TAIR        GGCTTGATAGTGCAAAAGACAAAGCTTATAAATGATTGCAACAGACGCCTAAAAGATCTT
                ************************************************************
BIG-GENOME      CTTGACGGACTTCTGCTTGAAAGCAGTGAAAATAAACGTCAATTTATCCGTGCCTGTGTT
BIG-ORF         CTTGACGGACTTCTGCTTGAAAGCAGTGAAAATAAACGTCAATTTATCCGTGCCTGTGTT
BIG-TAIR        CTTGACGGACTTCTGCTTGAAAGCAGTGAAAATAAACGTCAATTTATCCGTGCCTGTGTT
                ************************************************************
BIG-GENOME      TCTGGACTGCAAACCCATGCAGAGGAGAACAAAGGACGTACCTGTTTGGTAAGAAGAATT
BIG-ORF         TCTGGACTGCAAACCCATGCAGAGGAGAACAAAGGACGTACCTGTTTG------------
BIG-TAIR        TCTGGACTGCAAACCCATGCAGAGGAGAACAAAGGACGTACCTGTTTG------------
                ************************************************
BIG-GENOME      ATCATACTGACCACTGTATCATCTTCAAAATATATGATTCTTTATTACTTTACAGTCTCT
BIG-ORF         ------------------------------------------------------------
BIG-TAIR        ------------------------------------------------------------

BIG-GENOME      GGGGATCTTCTATTCTTCTTTTTAGACTTCTGGATGAAAATATTATATACACTTAGTTTG
BIG-ORF         ------------------------------------------------------------
BIG-TAIR        ------------------------------------------------------------

BIG-GENOME      AGTATTAGGAGTATTTTGAACAGTCTATATATAGCTGATATCCTTCCTAACTTTATATCC
BIG-ORF         ------------------------------------------------------------
BIG-TAIR        ------------------------------------------------------------

BIG-GENOME      TAGATTCCAGTTGACATATATCCTTGACTTGTGTTTTGATCATTTCAATTGCATATGCAG
BIG-ORF         ------------------------------------------------------------
BIG-TAIR        ------------------------------------------------------------

BIG-GENOME      TTTATTCTTGAGCAACTTTGTAATCTGATCTGCCCGTCAAAACCTGAGGCTGTGTATATG
BIG-ORF         TTTATTCTTGAGCAACTTTGTAATCTGATCTGCCCGTCAAAACCTGAGGCTGTGTATATG
BIG-TAIR        TTTATTCTTGAGCAACTTTGTAATCTGATCTGCCCGTCAAAACCTGAGGCTGTGTATATG
                ************************************************************
BIG-GENOME      CTGATCTTGAACAAATCACACACGCAAGAGGAGTTTATCCGGGGATCAATGACAAAAAAT
BIG-ORF         CTGATCTTGAACAAATCACACACGCAAGAGGAGTTTATCCGGGGATCAATGACAAAAAAT
BIG-TAIR        CTGATCTTGAACAAATCACACACGCAAGAGGAGTTTATCCGGGGATCAATGACAAAAAAT
                ************************************************************
BIG-GENOME      CCATATTCGAGTGCTGAGATTGGCCCACTGATGCGGGATGTGAAAAACAAGATTTGTCAG
BIG-ORF         CCATATTCGAGTGCTGAGATTGGCCCACTGATGCGGGATGTGAAAAACAAGATTTGTCAG
```

```
BIG-TAIR        CCATATTCGAGTGCTGAGATTGGCCCACTGATGCGGGATGTGAAAAACAAGATTTGTCAG
                ************************************************************
BIG-GENOME      CAGTTGGACCTGCTAGGTCTACTGGAAGATGACTATGGCATGGAGTTGCTTGTAGCAGGA
BIG-ORF         CAGTTGGACCTGCTAGGTCTACTGGAAGATGACTATGGCATGGAGTTGCTTGTAGCAGGA
BIG-TAIR        CAGTTGGACCTGCTAGGTCTACTGGAAGATGACTATGGCATGGAGTTGCTTGTAGCAGGA
                ************************************************************
BIG-GENOME      AACATCATTTCACTAGACCTGAGCATAGCTCAAGTTTATGAGCTAGTTTGGAAGAAATCA
BIG-ORF         AACATCATTTCACTAGACCTGAGCATAGCTCAAGTTTATGAGCTAGTTTGGAAGAAATCA
BIG-TAIR        AACATCATTTCACTAGACCTGAGCATAGCTCAAGTTTATGAGCTAGTTTGGAAGAAATCA
                ************************************************************
BIG-GENOME      AATCAGTCTTCTACTTCACTGACCAACTCCGCACTGTTAGCTTCAAATGCAGCTCCCAGC
BIG-ORF         AATCAGTCTTCTACTTCACTGACCAACTCCGCACTGTTAGCTTCAAATGCAGCTCCCAGC
BIG-TAIR        AATCAGTCTTCTACTTCACTGACCAACTCCGCACTGTTAGCTTCAAATGCAGCTCCCAGC
                ************************************************************
BIG-GENOME      AGGGATTGCCCTCCCATGACGGTGACATACAGACTTCAGGTTTGTGACGCTTTTCTAGTG
BIG-ORF         AGGGATTGCCCTCCCATGACGGTGACATACAGACTTCAG---------------------
BIG-TAIR        AGGGATTGCCCTCCCATGACGGTGACATACAGACTTCAG---------------------
                ***************************************
BIG-GENOME      AAGTACTAGGCCAAAGATGGGTTTTGCTCGCCTTTACTTATTGTTTTTGATAAAAGCGGC
BIG-ORF         ------------------------------------------------------------
BIG-TAIR        ------------------------------------------------------------

BIG-GENOME      AAATTCATACAATATTGTGTGGTTAAAATATTGGCAGGGGTTAGATGGTGAAGCTACTGA
BIG-ORF         -------------------------------------GGGTTAGATGGTGAAGCTACTGA
BIG-TAIR        -------------------------------------GGGTTAGATGGTGAAGCTACTGA
                                                     ***********************
BIG-GENOME      GCCTATGATCAAGGAATTGGAAGAAGATAGAGAAGAATCACAAGATCCAGAGATTGAGTT
BIG-ORF         GCCTATGATCAAGGAATTGGAAGAAGATAGAGAAGAATCACAAGATCCAGAGATTGAGTT
BIG-TAIR        GCCTATGATCAAGGAATTGGAAGAAGATAGAGAAGAATCACAAGATCCAGAGATTGAGTT
                ************************************************************
BIG-GENOME      TGCAATAGCAGGTGCAGTTCGAGAATATGGTGGTCTGGAAATTTTACTTGATATGATCAA
BIG-ORF         TGCAATAGCAGGTGCAGTTCGAGAATATGGTGGTCTGGAAATTTTACTTGATATGATCAA
BIG-TAIR        TGCAATAGCAGGTGCAGTTCGAGAATATGGTGGTCTGGAAATTTTACTTGATATGATCAA
                ************************************************************
BIG-GENOME      GGTAAGGATCTTAAACTATCAATGGGAAACTGAGATTCTCTCCTAGGCTCTTAGGCACTT
BIG-ORF         G-----------------------------------------------------------
BIG-TAIR        G-----------------------------------------------------------
                *
BIG-GENOME      TTCGATACACATTGAAGGAGCGTAAAAGAAGAATAATTATAATACCAAAGTAGCTTATTA
BIG-ORF         ------------------------------------------------------------
BIG-TAIR        ------------------------------------------------------------

BIG-GENOME      ACATGTAATGTATTCCTATGCAGAGTTTACAAGATGACTTCAAATCCAACCAAGAAGAGA
BIG-ORF         -----------------------AGTTTACAAGATGACTTCAAATCCAACCAAGAAGAGA
```

```
BIG-TAIR        -----------------------AGTTTACAAGATGACTTCAAATCCAACCAAGAAGAGA
                                       *************************************
BIG-GENOME      TGGTTGCAGTTCTTGATCTCCTAAACCATTGCTGCAAGATTAGAGAGAACAGGCGAGCTC
BIG-ORF         TGGTTGCAGTTCTTGATCTCCTAAACCATTGCTGCAAGATTAGAGAGAACAGGCGAGCTC
BIG-TAIR        TGGTTGCAGTTCTTGATCTCCTAAACCATTGCTGCAAGATTAGAGAGAACAGGCGAGCTC
                ************************************************************
BIG-GENOME      TACTCAGGCTAGGGGCTTTAAGCTTACTTCTTGAAACAGCTAGGAGGGCATTTTCTGTGG
BIG-ORF         TACTCAGGCTAGGGGCTTTAAGCTTACTTCTTGAAACAGCTAGGAGGGCATTTTCTGTGG
BIG-TAIR        TACTCAGGCTAGGGGCTTTAAGCTTACTTCTTGAAACAGCTAGGAGGGCATTTTCTGTGG
                ************************************************************
BIG-GENOME      ATGCTATGGAGCCAGCTGAAGGCATACTCTTAATTGTCGAGAGTTTGACGCTTGAAGCAA
BIG-ORF         ATGCTATGGAGCCAGCTGAAGGCATACTCTTAATTGTCGAGAGTTTGACGCTTGAAGCAA
BIG-TAIR        ATGCTATGGAGCCAGCTGAAGGCATACTCTTAATTGTCGAGAGTTTGACGCTTGAAGCAA
                ************************************************************
BIG-GENOME      ATGAAAGTGACAGCATCAGTGCCGCACAAAGTGCTTTGACTGTCAGTAACGAGGAAACTG
BIG-ORF         ATGAAAGTGACAGCATCAGTGCCGCACAAAGTGCTTTGACTGTCAGTAACGAGGAAACTG
BIG-TAIR        ATGAAAGTGACAGCATCAGTGCCGCACAAAGTGCTTTGACTGTCAGTAACGAGGAAACTG
                ************************************************************
BIG-GENOME      GAACTTGGGAACAGGCCAAAAAAATAGTGCTTATGTTCTTAGAAAGGCTAAGCCATCCTT
BIG-ORF         GAACTTGGGAACAGGCCAAAAAAATAGTGCTTATGTTCTTAGAAAGGCTAAGCCATCCTT
BIG-TAIR        GAACTTGGGAACAGGCCAAAAAAATAGTGCTTATGTTCTTAGAAAGGCTAAGCCATCCTT
                ************************************************************
BIG-GENOME      CAGGGCTTAAAAAGTCAAACAAACAGCAAAGGAACACGGAGATGGTTGCTAGAATCTTAC
BIG-ORF         CAGGGCTTAAAAAGTCAAACAAACAGCAAAGGAACACGGAGATGGTTGCTAGAATCTTAC
BIG-TAIR        CAGGGCTTAAAAAGTCAAACAAACAGCAAAGGAACACGGAGATGGTTGCTAGAATCTTAC
                ************************************************************
BIG-GENOME      CTTACTTAACATACGGTGAGCCTGCAGCGATGGAAGCACTCATAGAGCATTTTAGCCCTT
BIG-ORF         CTTACTTAACATACGGTGAGCCTGCAGCGATGGAAGCACTCATAGAGCATTTTAGCCCTT
BIG-TAIR        CTTACTTAACATACGGTGAGCCTGCAGCGATGGAAGCACTCATAGAGCATTTTAGCCCTT
                ************************************************************
BIG-GENOME      ACCTGCAAAACTGGTCTGAATTTGATCAGCTTCAGCAACGTCATGAGGAGGACCCCAAGG
BIG-ORF         ACCTGCAAAACTGGTCTGAATTTGATCAGCTTCAGCAACGTCATGAGGAGGACCCCAAGG
BIG-TAIR        ACCTGCAAAACTGGTCTGAATTTGATCAGCTTCAGCAACGTCATGAGGAGGACCCCAAGG
                ************************************************************
BIG-GENOME      ATGACAGCATTGCTCAGCAAGCGGCGAAGCAGAGGTTTACTGTGGAGAATTTTGTAAGAG
BIG-ORF         ATGACAGCATTGCTCAGCAAGCGGCGAAGCAGAGGTTTACTGTGGAGAATTTTGTAAGAG
BIG-TAIR        ATGACAGCATTGCTCAGCAAGCGGCGAAGCAGAGGTTTACTGTGGAGAATTTTGTAAGAG
                ************************************************************
BIG-GENOME      TGTCAGAATCTCTCAAGACAAGTTCATGTGGAGAGAGGTTAAAAGACATAGTTTTGGAGA
BIG-ORF         TGTCAGAATCTCTCAAGACAAGTTCATGTGGAGAGAGGTTAAAAGACATAGTTTTGGAGA
BIG-TAIR        TGTCAGAATCTCTCAAGACAAGTTCATGTGGAGAGAGGTTAAAAGACATAGTTTTGGAGA
                ************************************************************
BIG-GENOME      ATGGTATCATAGCTGTTGCTGTCAAACACATAAAGGAAATTTTTGCAATCACGGGACAGA
BIG-ORF         ATGGTATCATAGCTGTTGCTGTCAAACACATAAAGGAAATTTTTGCAATCACGGGACAGA
```

```
BIG-TAIR        ATGGTATCATAGCTGTTGCTGTCAAACACATAAAGGAAATTTTTGCAATCACGGGACAGA
                ************************************************************
BIG-GENOME      CTGGGTTCAAATCTAGCAAGGAATGGCTTTTGGCTCTTAAACTTCCGTCAGTGCCTCTTA
BIG-ORF         CTGGGTTCAAATCTAGCAAGGAATGGCTTTTGGCTCTTAAACTTCCGTCAGTGCCTCTTA
BIG-TAIR        CTGGGTTCAAATCTAGCAAGGAATGGCTTTTGGCTCTTAAACTTCCGTCAGTGCCTCTTA
                ************************************************************
BIG-GENOME      TTTTGTCAATGCTAAGAGGATTGTCGATGGGTCATTTGCCTACCCAAACATGCATAGACG
BIG-ORF         TTTTGTCAATGCTAAGAGGATTGTCGATGGGTCATTTGCCTACCCAAACATGCATAGACG
BIG-TAIR        TTTTGTCAATGCTAAGAGGATTGTCGATGGGTCATTTGCCTACCCAAACATGCATAGACG
                ************************************************************
BIG-GENOME      AGGGAGGAATTTTAACCCTACTTCATGCCTTAGAAGGAGTCTCTGGAGAAAACGACATCG
BIG-ORF         AGGGAGGAATTTTAACCCTACTTCATGCCTTAGAAGGAGTCTCTGGAGAAAACGACATCG
BIG-TAIR        AGGGAGGAATTTTAACCCTACTTCATGCCTTAGAAGGAGTCTCTGGAGAAAACGACATCG
                ************************************************************
BIG-GENOME      GTGCAAGGGCTGAAAATTTGCTAGATACACTTGCCGATAAAGAAGGAAAAGGAGATGGAT
BIG-ORF         GTGCAAGGGCTGAAAATTTGCTAGATACACTTGCCGATAAAGAAGGAAAAGGAGATGGAT
BIG-TAIR        GTGCAAGGGCTGAAAATTTGCTAGATACACTTGCCGATAAAGAAGGAAAAGGAGATGGAT
                ************************************************************
BIG-GENOME      TTCTTGGAGAGAAAGTCCGTGCATTACGAGATGCCACCAAAGATGAAATGAGACGCCGTG
BIG-ORF         TTCTTGGAGAGAAAGTCCGTGCATTACGAGATGCCACCAAAGATGAAATGAGACGCCGTG
BIG-TAIR        TTCTTGGAGAGAAAGTCCGTGCATTACGAGATGCCACCAAAGATGAAATGAGACGCCGTG
                ************************************************************
BIG-GENOME      CATTAAGAAAGAGAGAGGAGCTCCTGCAGGTAAATATAATAATGATTCTCTCTCAAGGAA
BIG-ORF         CATTAAGAAAGAGAGAGGAGCTCCTGCAG-------------------------------
BIG-TAIR        CATTAAGAAAGAGAGAGGAGCTCCTGCAG-------------------------------
                *****************************
BIG-GENOME      ACTAAATGTGTAATTTGGTCTTGTGACAATATTTACCAAGTTTCTCTTTTGAAACAGGGA
BIG-ORF         ---------------------------------------------------------GGA
BIG-TAIR        ---------------------------------------------------------GGA
                                                                         ***
BIG-GENOME      CTTGGAATGCGTCAAGAGCTATCTTCTGATGGTGGTGAAAGGATTGTGGTTTCTCAACCA
BIG-ORF         CTTGGAATGCGTCAAGAGCTATCTTCTGATGGTGGTGAAAGGATTGTGGTTTCTCAACCA
BIG-TAIR        CTTGGAATGCGTCAAGAGCTATCTTCTGATGGTGGTGAAAGGATTGTGGTTTCTCAACCA
                ************************************************************
BIG-GENOME      ATTCTTGAAGGTTTTGAGGATGTAGAGGAAGAAGAAGATGGATTGGCTTGCATGGTGTGT
BIG-ORF         ATTCTTGAAGGTTTTGAGGATGTAGAGGAAGAAGAAGATGGATTGGCTTGCATGGTGTGT
BIG-TAIR        ATTCTTGAAGGTTTTGAGGATGTAGAGGAAGAAGAAGATGGATTGGCTTGCATGGTGTGT
                ************************************************************
BIG-GENOME      AGAGAAGGCTACAAGCTCAGACCTTCTGATTTATTAGGAGTCTACTCTTACAGCAAACGG
BIG-ORF         AGAGAAGGCTACAAGCTCAGACCTTCTGATTTATTAGGAGTCTACTCTTACAGCAAACGG
BIG-TAIR        AGAGAAGGCTACAAGCTCAGACCTTCTGATTTATTAGGAGTCTACTCTTACAGCAAACGG
                ************************************************************
BIG-GENOME      GTGAATCTCGGTGTTGGCAATTCTGGAAGCGCACGTGGTGAATGCGTTTACACTACAGTG
BIG-ORF         GTGAATCTCGGTGTTGGCAATTCTGGAAGCGCACGTGGTGAATGCGTTTACACTACAGTG
```

```
BIG-TAIR        GTGAATCTCGGTGTTGGCAATTCTGGAAGCGCACGTGGTGAATGCGTTTACACTACAGTG
                ************************************************************
BIG-GENOME      AGCTACTTCAACATAATTCATTTTCAATGTCATCAAGAGGCAAAAAGAGCCGACGCTGCT
BIG-ORF         AGCTACTTCAACATAATTCATTTTCAATGTCATCAAGAGGCAAAAAGAGCCGACGCTGCT
BIG-TAIR        AGCTACTTCAACATAATTCATTTTCAATGTCATCAAGAGGCAAAAAGAGCCGACGCTGCT
                ************************************************************
BIG-GENOME      CTTAAAAATCCGAAGAAAGAATGGGAAGGAGCTATGTTGCGTAACAATGAATCTCTTTGC
BIG-ORF         CTTAAAAATCCGAAGAAAGAATGGGAAGGAGCTATGTTGCGTAACAATGAATCTCTTTGC
BIG-TAIR        CTTAAAAATCCGAAGAAAGAATGGGAAGGAGCTATGTTGCGTAACAATGAATCTCTTTGC
                ************************************************************
BIG-GENOME      AACTCTCTCTTCCCTGTCAAGGGTCCCTCAGTTCCCTTAGCACAGTATCTTCGTTACGTT
BIG-ORF         AACTCTCTCTTCCCTGTCAAGGGTCCCTCAGTTCCCTTAGCACAGTATCTTCGTTACGTT
BIG-TAIR        AACTCTCTCTTCCCTGTCAAGGGTCCCTCAGTTCCCTTAGCACAGTATCTTCGTTACGTT
                ************************************************************
BIG-GENOME      GACCAGTACTGGGATAATCTCAATGCTCTTGGTCGAGCCGACGGAAGCAGACTCCGTCTA
BIG-ORF         GACCAGTACTGGGATAATCTCAATGCTCTTGGTCGAGCCGACGGAAGCAGACTCCGTCTA
BIG-TAIR        GACCAGTACTGGGATAATCTCAATGCTCTTGGTCGAGCCGACGGAAGCAGACTCCGTCTA
                ************************************************************
BIG-GENOME      TTGACGTACGACATTGTCCTGGTAAGTTAAAAATTGGTTCTGAAACTTACTAGGTGTTGG
BIG-ORF         TTGACGTACGACATTGTCCTG---------------------------------------
BIG-TAIR        TTGACGTACGACATTGTCCTG---------------------------------------
                *********************
BIG-GENOME      CCTTCGGTTCAATTGGCAGTTGGTTAAAAAGTGTCAATTCGGTTTGGTTTTAAACTTGAT
BIG-ORF         ------------------------------------------------------------
BIG-TAIR        ------------------------------------------------------------

BIG-GENOME      CGGGACTTGATTCCAAACAGCTAATCAAGTGGCTCTGTTGATTTCGTTTCGGATTTACCA
BIG-ORF         ------------------------------------------------------------
BIG-TAIR        ------------------------------------------------------------

BIG-GENOME      AGTGATTCACTGACAACTTTTGTTTCATCTGTAGATGCTGGCTCGGTTTGCAACAGGAGC
BIG-ORF         ----------------------------------ATGCTGGCTCGGTTTGCAACAGGAGC
BIG-TAIR        ----------------------------------ATGCTGGCTCGGTTTGCAACAGGAGC
                                                  **************************
BIG-GENOME      TTCTTTTAGCGCGGATTGCCGAGGTGGAGGAAGAGATAGCAATTCCCGTTTCTTGCCTTT
BIG-ORF         TTCTTTTAGCGCGGATTGCCGAGGTGGAGGAAGAGATAGCAATTCCCGTTTCTTGCCTTT
BIG-TAIR        TTCTTTTAGCGCGGATTGCCGAGGTGGAGGAAGAGATAGCAATTCCCGTTTCTTGCCTTT
                ************************************************************
BIG-GENOME      CATGTTCCAAATGGCCCGTCATCTTCTCGACCAAGGAGGCCCCGTGCAACGCACAAACAT
BIG-ORF         CATGTTCCAAATGGCCCGTCATCTTCTCGACCAAGGAGGCCCCGTGCAACGCACAAACAT
BIG-TAIR        CATGTTCCAAATGGCCCGTCATCTTCTCGACCAAGGAGGCCCCGTGCAACGCACAAACAT
                ************************************************************
BIG-GENOME      GGCTAGATCTGTTTCATCGTACATTTCATCATCATCGACATCTACAGCAACAGCGCCATC
BIG-ORF         GGCTAGATCTGTTTCATCGTACATTTCATCATCATCGACATCTACAGCAACAGCGCCATC
```

```
BIG-TAIR        GGCTAGATCTGTTTCATCGTACATTTCATCATCATCGACATCTACAGCAACAGCGCCATC
                ************************************************************
BIG-GENOME      CTCGGACTCGCGACCTTTAACTCCTGGATCCCAGCTATCATCAACTGGAACAGAGGAAAC
BIG-ORF         CTCGGACTCGCGACCTTTAACTCCTGGATCCCAGCTATCATCAACTGGAACAGAGGAAAC
BIG-TAIR        CTCGGACTCGCGACCTTTAACTCCTGGATCCCAGCTATCATCAACTGGAACAGAGGAAAC
                ************************************************************
BIG-GENOME      GGTTCAGTTCATGATGGTAAACTCGCTTCTATCAGAATCATATGAATCATGGCTCCAACA
BIG-ORF         GGTTCAGTTCATGATGGTAAACTCGCTTCTATCAGAATCATATGAATCATGGCTCCAACA
BIG-TAIR        GGTTCAGTTCATGATGGTAAACTCGCTTCTATCAGAATCATATGAATCATGGCTCCAACA
                ************************************************************
BIG-GENOME      CCGTAGGGTCTTTCTCCAGCGGGGGATTTACCATACTTTCATGCAACACGCTCACGGTCG
BIG-ORF         CCGTAGGGTCTTTCTCCAGCGGGGGATTTACCATACTTTCATGCAACACGCTCACGGTCG
BIG-TAIR        CCGTAGGGTCTTTCTCCAGCGGGGGATTTACCATACTTTCATGCAACACGCTCACGGTCG
                ************************************************************
BIG-GENOME      GGTAGCTTCTCGCGCTGCAGAACCAACCAGTTCAGGAGGCAAAACCCAAGACGCAGAAAC
BIG-ORF         GGTAGCTTCTCGCGCTGCAGAACCAACCAGTTCAGGAGGCAAAACCCAAGACGCAGAAAC
BIG-TAIR        GGTAGCTTCTCGCGCTGCAGAACCAACCAGTTCAGGAGGCAAAACCCAAGACGCAGAAAC
                ************************************************************
BIG-GENOME      CTTAACCGGCGATGAACTTCTCTCTATCGTCAAACCAATGCTGGTCTACACCGGTATGAT
BIG-ORF         CTTAACCGGCGATGAACTTCTCTCTATCGTCAAACCAATGCTGGTCTACACCGGTATGAT
BIG-TAIR        CTTAACCGGCGATGAACTTCTCTCTATCGTCAAACCAATGCTGGTCTACACCGGTATGAT
                ************************************************************
BIG-GENOME      CGAACAGCTTCAACAGTTGTTCAAACCCAAGAAACCGGTCCACATCGAACCAATCAAGAA
BIG-ORF         CGAACAGCTTCAACAGTTGTTCAAACCCAAGAAACCGGTCCACATCGAACCAATCAAGAA
BIG-TAIR        CGAACAGCTTCAACAGTTGTTCAAACCCAAGAAACCGGTCCACATCGAACCAATCAAGAA
                ************************************************************
BIG-GENOME      AGAAGGTACATCAAGTGGAGTAGAGCTTGAGCCATGGGAGATTGTAATGAAAGAGAAGCT
BIG-ORF         AGAAGGTACATCAAGTGGAGTAGAGCTTGAGCCATGGGAGATTGTAATGAAAGAGAAGCT
BIG-TAIR        AGAAGGTACATCAAGTGGAGTAGAGCTTGAGCCATGGGAGATTGTAATGAAAGAGAAGCT
                ************************************************************
BIG-GENOME      GCTGAATGTGAAAGAAATGATTGGTTTCTCCAAGGAATTGATCTCGTGGCTCGACGAAAT
BIG-ORF         GCTGAATGTGAAAGAAATGATTGGTTTCTCCAAGGAATTGATCTCGTGGCTCGACGAAAT
BIG-TAIR        GCTGAATGTGAAAGAAATGATTGGTTTCTCCAAGGAATTGATCTCGTGGCTCGACGAAAT
                ************************************************************
BIG-GENOME      CAACTCTGCAACTGATCTACAAGAAGCATTTGATATTGTCGGCGTTTTGGCTGATGTTTT
BIG-ORF         CAACTCTGCAACTGATCTACAAGAAGCATTTGATATTGTCGGCGTTTTGGCTGATGTTTT
BIG-TAIR        CAACTCTGCAACTGATCTACAAGAAGCATTTGATATTGTCGGCGTTTTGGCTGATGTTTT
                ************************************************************
BIG-GENOME      GTCTGAAGGAGTTACACAGTGTGATCAGTTTGTGAGATCGGCGATCGATAAAGATTGA
BIG-ORF         GTCTGAAGGAGTTACACAGTGTGATCAGTTTGTGAGATCGGCGATCGATAAAGATTGA
BIG-TAIR        GTCTGAAGGAGTTACACAGTGTGATCAGTTTGTGAGATCGGCGATCGATAAAGATTGA
                **********************************************************
```

3. Primer pairs used to amplify and sequence through the cDNA of *BIG* gene

Primer S1F: 5’-GGAACATCGTCTTCTCCACAG-3’

Primer S1R: 5’-TTTGAGCATCTGCCCCCTTG-3’

Primer S2F: 5’-TTTCTCGCCGTTTACGCTCC-3’

Primer S2R: 5’-TCTGAAAAACACTGCTCTTGCC-3’

Primer S3F: 5’-AACCACACTGCTACAGAGGG-3’

Primer S3R: 5’-GAAAATCAGACAGAGCACGAC-3’

Primer S4F: 5’-GTAGTATTGAAGACAAGGCT-3’

Primer S4R: 5’-ATGACTGGTGCTGATGAG-3’

Primer S5F: 5’-CCCCTCATCCTCCTACATTTC-3’

Primer S5R: 5’-AGTATCTACCCCAAACTTCTCC-3’

Primer S6F: 5’-CTGTCAAAACTGGATTCCCTG-3’

Primer S6R: 5’-CATTGCTGCCTCTCTGAAAG-3’

Primer S7F: 5’-TGCAGTCTCTGAGCATACC-3’

Primer S7R: 5’-TCCACAGTCACAGAAAAACC-3’

Primer S8F: 5’-CTTGTGACCTTACTGTGTCC-3’

Primer S8R: 5’-CTTTCCCGTAACATCCTTTCC-3’

Primer S9F: 5’-GCTACTCTTTTTGTTGCTTCTC-3’

Primer S9R: 5’-CATCCATTTTTTCTTTCCAGCC-3’

Primer S10F: 5’-TCTCCAATCCAAACCCTGAC-3’

Primer S10R: 5’-TGAACCCCTGTATCCTTTCC-3’

Primer S11F: 5’-CCGTAAATCAAATTTCTCCGTC-3’

Primer S11R: 5’-TCCATCCATCCACTCAACTC-3’

Primer S12F: 5’-AGCTTCAACTCCCTCCATC-3’

Primer S12R: 5’-CTTTTCCTTTTCCCCCATTTTC-3’

Primer S13F: 5’-GCAAAAGCCCATAGAGCAG-3’

Primer S13R: 5’-TCAAGCAACAAGGAGACAAG-3’

Primer S14F: 5’-AAATGGGGGAAAAGGAAAAGG-3’

Primer S14R: 5’-CGGGAGTTTGGATGATTGG-3’

Primer S15F: 5’-CGAACTCATTGCCAATCATCC-3’

Primer S15R: 5’-TCATCAAAACTCTACGAAGCC-3’

Primer S16F: 5’-ATTCGCAGCACAAGGACAC-3’

Primer S16R: 5’-TCCGACCTGATAGATTGAGAAC-3’

Primer S17F: 5’-TTGGAATGGGAGAAAGGGG-3’

Primer S17R: 5’-CGGGCAGATCAGATTACAAAG-3’

Primer S18F: 5'-TTTATCCGTGCCTGTGTTTC-3'

Primer S18R: 5'-GTGTTCCTTTGCTGTTTGTTTG-3'

Primer S19F: 5'-AAGTGACAGCATCAGTGCC-3'

Primer S19R: 5'-CTTCCTCTACATCCTCAAAACC-3'

Primer S20F: 5'-CCAAAGATGAAATGAGACGCC-3'

Primer S20R: 5'-TTGAAGCTGTTCGATCATACC-3'

Primer S21F: 5'-CATCAACTGGAACAGAGGAAAC-3'

Primer S21R: 5'-CGGTGGCTCAATAAACCAAAC-3'

4. The confirmed cDNA of *BIG* gene by PCR and DNA sequencing

ggaacatcgtcttctccacagagtcctctttcaccgcctttgattctttacttataaacccttaccttcctcttcc
ttttgtatatacctatttctctagaaacctttttcctcatcgaactcttcttctttcgcctcgccagaaATGGCAG
ATGACTTGGCGAATCTCTGCCGATTTCTCTTCGACGACACCGCCTTTCCCTCTTTGTCGTCTTCGGCATCTTCAGA
TCTCTTTTCTCGCCGTTTACGCTCCGATGATTCGATCAAGCGCGGTCTCCGTTCTTTCTATCTCCTCCTTCGCTGG
GGTGTCGCTCCGATCGGCGGAGACGATGCTGATTCCTCTGGCAAGCTACGCTTTGAGACATGGTCCGATTCGCAGC
TTCAAGCTCTTGTTTCGATATCTCAGGCGATCCTTCTACTATCTCGATCTCTATTAGTGGACCAACTCGAACCAAT
CGTTTTGGGTGTCATCCAGGAGGTGATGGAATTCTCTCTCAGCTTCTTGGAGAAGTCAAGTTTTAGACAAAATGAT
CTCAAAATGGAGATCAACATGGAAATACTTTTGGAAATTGCTTCTTTTGATGGAAGTGAGAAGCAATATGATATAT
TACCAGATTTCTCTCCCGCTGAAGTTGCAGAACTGTGGCCAGCTTTTTCTGGCGAGCATGATAATATGGACGCACA
GAGCCTTGTGAAATGTACTTTTCAAGGGGGCAGATGCTCAAATGAAGAGAAGCCAGTTGACCGGCTTCTTATTACA
CTGATGTCTGAATGCATTGAATCTGATGTCCAGGCGCAGTCAGTAGTAAAGTCCCCTTTTCAACAAGACTGTGGAG
ACCTGAATCCCTTTACTCGACACCTGGCAGTTGTTCACCTTCGCTGTGTTTGTCGCCTGATTATGGTCTGCAAAGA
GCTTGTTCAATTGCCAAATATGTTGGATGAGAAGACGGTTGATCAAGCTGTCCTTGATAAATTGTCGTTCTGCTTA
AGAATTTTGAAGCTGCTTGGGAGTCTTTCAAAAGATGTTCAAAGTATAGAAAACGACGGCTCATTGTTACAAGCAG
TAGCTTCATTTACAGATGCTTTTCCTAAGCTGTTTAGGGTTTTCTTTGAATTCACTAACCACACTGCTACAGAGGG
TAATATTGAGAGTCTTTCTCTTGCGCTAGTGGAGGGATTTCTTAATCTCGTCCAACTCATTTTTGGCAAGAGCAGT
GTTTTTCAGAATGTCCAGGCATGTGTGGCAGCTTCTATTGTCAGCAATCTAGATTCCTCAGTGTGGAGATATGATG
GGTCCTCTTGTAATTTGACACCTCCACTTGCTTACTTCCCTCGGTCTGTCATCTACACATTGAAACTCATTCAAGA
TCTCAAGAGACAACCATACCATATCCATGATCTGAGGGTTTTAGAGTCTGAAGTTACCTATGAAGATGTCAGTTCC
ACTGTTGATTCTGTTTATTTCCATCTGCGCCAGGAAAAGATCCCGTTGCTCAAGTGCTTCACGGTCGAGGATATAA
TGAGAGTGATATTTCCTTCATCAAGTCAATGGATGGATAACTTCTTCCATCTTGTTTATTTCCTCCATCGCGAAGG
TGTAAAATTACGACCAAAAGTGGAGAGGACGTATTCCAGTTTAAGATCCAACAGTTTTGCAGAAGTTGAAAGCCAA
ATTTCTCATGACGATGAAGCCCTGTTTGGAAACTTATTTTCTGAGGGTAGTCGTTCTCTCTGTTCCATAGAACCAA
ATGATCAACCACCTGTTTCTGTCAGCAGCAATTTACTATTGCAAGCTGCTAAGGAACTGTTGAATTTTCTTAGAGC
ATGCATTCTTTGTCAAGAATGGGTTCCTAGTATATATGAAGATGGATGTAAAAAGCTTGACACTGGTCACATTGAT
ATCCTGCTCAATATAGTAGGCTGTAGTATTGAAGACAAGGCTTCTGATGGTGGTTGCATGCTCCAAGATGAGGGAA
GACCTGGACATGTTGCTTTTGAGCTGTTACTCAATCTCTTGAGAAGTCGTGCTCTGTCTGATTTTCTAGAGTCCTA
CCTTTTTCAGCAAATTCTTGTTGTTGAAAATAGTGACTTCAACTATAATGATAAGACTCTGGCACTGTTGGCTCAC
ACTCTTCTATGTAGGCCGGGCTTGGCTGGGGCACAGTTGAGAGCCAAAATTTATGATGGTTTCGTTAGTTTTGTTA
CCGAGAGAGCAAGAGGTATATGTGCTGAAGCTTTGAGTCTCAAGGAGTTGACTGCTTGTCTTCCTTCTGCTTTCCA
TATTGAGATTCTTCTTATGGCTTTCCATTTATCAAATGAAGCTGAGAAGGCAAAATTCTCAAATCTCATTGCTTCA
TGTCTGCATAAAGTTGATACCCCGGCAGGAATATGTGATGGTCCTCAGCTGTCCTCTTGGGCAATGCTGATATCTA

GGTTGTTAGTGCTGTTGCACCATATGTTGTTACATCCAAACACATGCCCAACATCATTAATGCTAGATCTAAGGTC
TAAACTGAGGGAAGTTCGTAGCTGTGGTAGTAATTTACATGTAACTGTTGGTGATCATTTATCTTCGTGGGCGTCT
CTTGTAGCAAGGGGTATAACTGATTCATGGGCTGAGGATGAGTCAGTCAGCCATCTAATGAGTCAAATGATTGACT
TTTCCCCTCATCCTCCTACATTTCAGAATGATGTGTCTACTGCCAAAACCTTAAACTTGGATTATGGAGATTTATC
TGCGAGTTTGTGTCGGGTTTTGGGGCTGTGGAAGGGGAAAAAGGCAGGAAAAGTGGAAGACCTGCTAGTGGAAAGA
TACATTTTCATGCTTTCTTCAGATATTGCTCGTATTAACTGTGCATTGGATAGCCAGCCTTCTTTGCATGTTAATT
ACCAGAATGTGGACATATCTAACTCTGTGGATCTCATCAGCACCAGTCATTTGCTTGTAGGCGACATCAATGTTGT
TGGTAGAAATATCGAGTTGAGAAACATTCTGATCGGTGTTTTAAATCAGCTCCAGGCAGCACCTGAGCAGGTGGTT
GAGGATTTGGGTTGGGATTATATTCGCGAAGGAGCTTGGCTCTCTCTTCTGTTGTACTTTCTTGATGGTGGCGTCT
GGGATTATTGCAACAAAAATTCATGTTCAGAAATCGATCCCTTCTGGAAGGAGTGCACATCTGTTGACGCCAAGTA
TGTTGCTGCAGCAGAAGGGGTAGTCTCTTACTTGATGAAGACTGGTGATATTGCAGAATTGCTGAGAATGCTTTCA
TCATTGGTTGGCAAATATTTACGAGTGTATAAGAAAGCTTTCCTTGCAACTTTCAGCGATTGGAATCATCACGGTC
ATAGTTCGCCTTCTCTGCTACTTCTCAAGCATACTCAATTTGGTAAAAGTCTCCAAGGTGAATATGCGAAGATTGG
TGACAATTCTCTCCATCTTCAATGCATCTTTTATCTGTCAAAACTGGATTCCCTGGGAGATGGAAGAGGTTCGGGT
GTTTTATGGAAAGTGTTTTGGGAATTTATGGTACATGGGTTTCCCACTAGTCTTCAAACTTCTAGTGCGATTCTTC
TTTCATGTATTCTAAGCATAAGATGCATTGTGCTGACAATAAATGGCTTACTCAAACTGGGTAACTCTAAGGAGAA
GTTTGGGGTAGATACTAGTGTACTTCATCAGCTACTTGATTCTATCATGATTATTAAGTTTGACCAGGTATTCGAA
AGCTTTCATGGGAAATGTGAGGAGATCCATCAAAATATATGTGCCGTGTTGCAGCTTCCAGATTTGACTGAGTTGT
TTCTGATGAAAGACATGGAGGGGTTTGTAAGAGATATCAGTGCTGAGCAGATAGATAGAAGCCAAGTGCTTGAAGG
GGTGATCACCAAGATTGTAGATGTTATGGACAGTCTAAGCAAGGATTCTTCGAAATCTGATATTTTCAAATTTTAT
CTTGGTGTAGATGCAGTCTCTGAGCATACCAGGGAATTTTATGAGTTGCAACGTGGAGATCTGTCTGTGTTTATTG
ACTCATTGGACTACTGTTCCTTGGAACCAGTAAACATAAAAGTACTTAACTTCCTTGTTGACCTTTTGTCTGTGGC
TCAATCCCCTGATCTCCGGAGAAGAGTACAACAAAAATTTATTGACATGGATTTGATATCTTTGTCTGGGTGGCTG
GAGAGAAGACTGTTGGGTTCCTTCGTTGAAGAAATAGATGGGAAAAAGACTGCAAAAGGAAATTCTCTTCCTTTCA
GAGAGGCAGCAATGAATTTTATTAATTGTCTTGTGTCATCTACTAACGACCTACAGACCAGAGAGTTACAGAATCA
TTTATTCGAGGCCTTGCTGATCTCCCTCGATACCGCGTTTCTCTCATTTGATATCCATATGTCTATGTCATATTTT
CATTTTGTTCTTCAACTTGCAAGGGAAGACAACCTGATGAAGATGGTTTTAAAGAGAACCATAATGCTGATGGAGA
AGCTTGCTGCCGAGGAGAAGTTGCTTCCTGGTTTGAAGTTTATTTTTGGTGTGATAGGCACCTTGTTGAGCAACCG
TTCTCCAAGTCATGGAGAGAGTTTGTGTGGAAAGTCCTTGGCAAGCTATAAGAATACTGCAACAGGTCCTTTGGTC
CCAAAACTTTCAGGAACAACGAAAAAATCTGATACATTGGCTCTCCCCGTGGATCAGGAAGGAAGCTCAATATCAC
TTGAATGCGATGTCACTTCTGTTGATGAAGATGAAGATGATGGAACATCTGATGGTGAAGTGGCCAGCTTAGACAA
GGAAGATGAAGAAGATGCCAACAGTGAGAGGTACCTTGCCTCAAAAGTCTGCACTTTTACGTCCAGTGGCAGTAAT
TTCATGGAACAACACTGGTATTTTTGTTACACTTGTGACCTTACTGTGTCCAAAGGTTGCTGTTCTGTTTGCGCGA
AAGTTTGCCACCGGGGTCACCGTGTTGTCTATTCACGATCGAGTCGGTTTTTCTGTGACTGTGGAGCTGGAGGTGT
TAGGGGAAGCAGCTGCCAGTGCCTGAAGCCACGCAAATATAATGGAAATGGAAGTGCTCCAGCTCGTGGTACAAAT
AATTTCCAATCGTTTTTACCCTTGTCTGAGGATGCAGATCAGCTTGGAGAAAGCGATTCGGATGTGGAAGAAGATG
GTTTCGGGGAGGAAAATCACGTTGTCTTATATATCCCTAAAGAAACTCAATATAAGATGTCACTGCTACTCGAAGA
GCTCGGGATAGAGGACCGAGTACTGGAGCTTTTCTCATCTTTACTTCCGTCTATCACTAGCAAAAGAGACTCTGGC
CTGTCGAAAGAGAAGCAGGTCAATCTCGGGAAAGACAAAGTTCTTTCATTTGACACGGATCTTTTGCAGCTGAAAA
AAGCATATAAAAGTGGGTCATTGGACTTAAAAATAAAGGCTGATTATACTAATTCAAAGGACCTTAAATCTCTTTT
AGCCAATGGTTCCCTTGTCAAGTCTCTCCTGAGTGTTAGTGTTCGGGGTCGCCTTGCAGTTGGAGAAGGTGATAAA
GTTGCTATATTTGATGTTGGACAGCTAATAGGACAAGCCACAATTGCACCCATAAATGCAGACAAGGCCAACGTTA
AACCACTTTCAAGGAATATTGTTCGTTTTGAGATTGTGCATCTTTCATTCAACCCAGTTGTGGAGAATTATCTTGC
TGTCGCAGGCCTTGAAGATTGCCAGATACTTACTTTGAATCATCGAGGTGAAGTCATCGACAGGCTTGCTGTTGAG

CTTGCCCTGCAAGGTGCATTCATCAGGCGTATAGACTGGGTTCCTGGTTCACAGGTTCAGTTAATGGTCGTTACGA
ACAAATTCGTGAAGATTTATGATCTATCCCAGGATAGCATCAGTCCAACACAGTACTTCACTTTGCCAAACGACAT
GATTGTGGATGCTACTCTTTTTGTTGCTTCTCGTGGGAGGGTTTTCCTTCTTGTTCTTTCAGAACAAGGGAATTTG
TATAGGTTCGAACTATCTTGGGGCGGCAATGCAGGCGCAACACCGCTTAAGGAAATCGTTCAGATTATGGGAAAGG
ATGTTACGGGAAAGGGTTCATCTGTCTATTTCTCTCCAACATATCGACTGCTTTTCATATCCTATCATGATGGAAG
TTCTTTTATGGGTCGACTCAGCTCAGATGCAACATCCTTAACTGATACATCTGGCATGTTTGAGGAAGAATCAGAT
TGTAAACAAAGGGTGGCTGGATTGCATCGTTGGAAAGAGTTGTTGGCTGGCAGTGGATTATTTATTTGCTTCTCCA
GCGTGAAGTCAAATGCTGTCTTAGCTGTGTCCTTGAGGGGCGATGGGGTATGTGCACAGAATCTCCGTCATCCGAC
AGGATCATCTTCCCCTATGGTTGGAATAACCGCATACAAACCTTTGTCAAAAGACAATGTTCACTGTCTAGTTCTG
CATGATGATGGCAGCCTTCAGATTTATTCTCATGTTCGTAGTGGAGTTGATACTGACTCAAATTTCACAGCTGAAA
AAGTTAAAAAGTTGGGTTCTAAGATACTTAACAACAAAACCTATGCTGGTGCAAAGCCAGAGTTTCCCCTGGATTT
CTTTGAGAGGGCATTTTGCATTACAGCAGATGTGAGACTTGGTAGTGATGCTATTAGAAATGGTGATTCCGAGGGA
GCAAAACAGAGCTTGGCATCTGAGGATGGCTTTATTGAGAGCCCCAGTCCCGTGGGCTTCAAGATATCTGTCTCCA
ATCCAAACCCTGACATTGTTATGGTTGGCATCCGGATGCATGTGGGTACTACATCAGCAAGCTCTATACCTTCAGA
AGTGACCATTTTCCAGAGATCGATTAAGATGGATGAGGGCATGAGGTGCTGGTATGACATCCCATTTACTGTGGCT
GAGTCACTTCTAGCTGATGAAGATGTTGTAATCTCTGTGGGGCCGACTACTAGTGGGACTGCACTGCCCAGAATAG
ACTCGCTTGAAGTATATGGTCGAGCTAAAGATGAATTTGGCTGGAAAGAAAAAATGGATGCTGTGCTAGATATGGA
AGCTCGTGTGCTTGGTCATGGTTTGCTTCTTCCAGGCTCTAGTAAAAAGAGAGCGTTGGCACAGTCTGCTTCAATG
GAAGAGCAAGTTATCGCTGATGGTCTTAAGCTCCTATCAATCTATTATTCAGTTTGTAGGCCACGGCAAGAAGTAG
TGCTTAGCGAACTCAAATGCAAACAGCTACTAGAGACGATTTTCGAAAGTGATAGGGAAACCCTATTACAAACGAC
AGCTTGCCGTGTTTTGCAGTCTGTCTTTCCAAGAAAGGAGATATACTACCAGGTCAAAGACACCATGCGACTCCTT
GGAGTGGTCAAGGTGACCTCCATTCTTTCTTCGAGGTTGGGGATTTTGGGTACTGGAGGTTCGATTGTTGAAGAGT
TCAATGCTCAGATGCGGGCGGTGTCTAAAGTAGCTTTGACCCGTAAATCAAATTTCTCCGTCTTTCTGGAGATGAA
TGGCTCTGAAGTGGTTGATAATCTGATGCAAGTGCTATGGGGAATTTTGGAGTCAGAGCCACTCGACACACCTACT
ATGAACAATGTCGTGATGTCCTCCGTTGAACTAATCTATAGCTATGCAGAGTGTTTGGCATCTCAAGGAAAGGATA
CAGGGGTTCATTCTGTAGCTCCTGCAGTTCAGTTACTGAAAGCACTTATGTTGTTTCCCAATGAGTCTGTGCAAAC
ATCCAGCAGCCTAGCTATATCATCAAGGTTACTTCAGGTTCCTTTCCCAAAGCAAACAATGTTGACAACAGATGAT
TTGGTTGACAATGTTACAACTCCTTCGGTGCCTATCAGAACAGCTGGTGGAAATACACATGTCATGATTGAGGAGG
ACTCTATAACTTCATCTGTTCAATACTGCTGTGATGGCTGCTCCACCGTCCCTATACTGAGAAGAAGGTGGCACTG
CACTGTTTGTCCAGATTTTGATCTATGTGAGGCATGCTATGAAGTGTTAGATGCAGATCGTCTACCACCACCACAC
ACTCGAGATCATCCTATGACAGCAATTCCAATAGAAGTGGAATCACTTGGTGCAGACACCAATGAGATTCAGTTCT
CTGCGGATGAAGTAGGTATTTCAAATATGTTGCCGGTGGTAACCAGTAGCATTCCACAAGCTTCAACTCCCTCCAT
CCATGTCTTGGAACCTGGTGAATCTGCTGAGTTCTCTGCCTCACTGACCGATCCTATTTCTATCTCAGCGTCAAAG
CGTGCCGTAAATTCTTTGATTCTCTCTGAGTTTCTCCAAGAGTTGAGTGGATGGATGGAAACAGTATCCGGTGTTC
AAGCTATTCCTGTGATGCAATTGTTCTACAGATTATCATCTGCCATTGGTGGAGCCTTTATGGATAGCTCAAAGCC
CGAAGAAATAAGCTTGGATAAGTTAATTAAATGGCTTTTGGGTGAAATCAATCTCAGCAAGCCGTTTGCTGCTTCA
ACTCGCTCTTCCTTAGGGGAAATTGTGATTCTTGTGTTTATGTTTTTCACCTTGATGCTTCGGAGTTGGCACCAGC
CTGGTAGTGATGGTAGTAGCTCCAAATTAGGTGGAAGTACAGATGTACATGACCGGAGGATTGTCCAGAGCTCCAC
TGTAGTAGCTACTCAGTCTTCTTTACATGTTCAAGAGAGAGATGACTTTGCATCTCAGCTAGTTCGTGCGTGCAGT
TGCCTCAGGAACCAAGAGTTTGTTAATTATCTGATGAACATACTTCAACAGCTAGTGCATGTTTTTAAGTCACGTG
CTGCCAATGTTGAAGCTCGTGGATCAAGCTCTGGTTCTGGTTGTGGAGCCATGCTCACAGTCAGAAGAGATTTACC
TGCGGGTAACTATTCTCCCTTCTTTTCGGATTCATATGCAAAAGCCCATAGAGCAGATATTTTTGTGGACTATCAC
AGGCTGCTACTTGAGAATGTGTTTCGCTTAGTATATACCTTAGTTAGACCTGAAAAACAAGAGAAAATGGGGGAAA
AGGAAAAGGTATACAGGAATGCTTCTTCTAAAGATTTAAAGCTGGACGGTTTCCAGGATGTTCTATGCAGCTATAT

AAATAACCCTCATACGGCTTTTGTTAGGAGATATGCTAGAAGGCTGTTTTTGCATCTTTGCGGAAGCAAAACTCAA
TACTACAGTGTCAGAGATTCATGGCAGTTTTCCAATGAAGTGAAAAACCTATACAAGCATGTTGAAAAGTCTGGTG
GTTTTGAAAACAATGTTTCATATGAAAGAAGTGTAAAGATTGTGAAGTCGCTTTCTACAATTGCTGAAGTTGCTGT
GGCAAGACCTCGGAACTGGCAGAAGTACTGTCTCAGGCATGGAGATTTTCTGTCCTTCCTACTGAATGGTGTATTC
CATTTTGCGGAAGAGTCTGTAATCCAGACACTTAAACTCCTCAATTTGGCCTTTTATCAAGGAAAAGATGTGAGCA
GCTCAGTGCAGAAAGCTGAAGCAACTGAAGTAGTGACAGGCTCAAACCGGTCAGGATCTCAGTCAGTGGATTCAAA
AAAGAAGAAAAAAAGGTGAAGATGGGCATGATTCGGGTTTGGAAAAATTATATGTGGATATGGAAGGGGTGGTCGAT
ATCTTCAGTGCCAATTGCGGAGATCTCTTGAGGCAGTTTATTGATTTCTTTCTGCTGGAATGGAATTCGAGCTCTG
TTCGCACTGAAGCAAAATCTGTTATTTATGGCTTGTGGCATCATGGAAGACACTCGTTCAAAGAAAGTCTGTTAGC
AGCTCTTTTGCAGAAGGTTAGATACCTGCCAGCATATGGTCAGAATATTGTTGAGTACACAGAACTTGTCTCCTTG
TTGCTTGACAAGGCTCCAGAAAACAACTCAAAGCAAGCAATTAATGAGCTTGTGGATCGGTGCCTGAACCCTGATG
TGATAAGGTGCTTTTTTGAGACGCTGCATTCCCAGAACGAACTCATTGCCAATCATCCAAACTCCCGTATATACAG
CACTCTGGGTAATCTTGTGGAATTTGATGGTTATTATCTTGAGAGCGAACCTTGTGTTGCCTGCAGTTCTCCTGAT
GTGCCATATAGCAAAATGAAGCTTGAAAGTTTGAAATCCGAGACAAAGTTTACTGATAACCGCATCATTGTGAAAT
GTACTGGGAGTTATACGATACAGTCTGTGACAATGAATGTTCATGATGCACGAAAGTCAAAGTCGGTGAAGGTCCT
AAACTTGTATTACAACAATCGCCCTGTCTCGGATTTATCAGAGCTTAAAAACAACTGGTCACTGTGGAAGCGTGCC
AAAAGTTGTCATCTGTCTTTTAACCAAACTGAACTGAAGGTGGAATTTCCTATTCCTATTACTGCTTGTAACTTCA
TGATCGAGTTGGATTCTTTCTATGAGAATCTTCAGGCGTTATCTCTTGAACCATTGCAATGTCCTCGATGCAGCCG
ACCTGTCACTGATAAACATGGTATTTGCAGCAACTGCCATGAGAATGCTTATCAGTGCAGGCAATGCCGTAACATT
AATTATGAAAATTTGGATTCATTCCTTTGCAACGAGTGTGGCTACAGCAAGTATGGGAGGTTTGAGTTTAACTTCA
TGGCAAAACCAAGCTTCATATTTGATAATATGGAAAATGACGAAGATATGAAGAAGGGATTGGCTGCTATAGAGTC
TGAGTCTGAAAACGCTCACAAGAGATATCAGCAACTACTGGGATTCAAGAAGCCGCTTCTGAAGATAGTTTCAAGC
ATTGGTGAAACTGAAATGGATTCGCAGCACAAGGACACTGTCCAGCAAATGATGGCATCTTTACCTGGTCCATCAT
GCAAGATAAACCGCAAAATCGCACTTTTGGGCGTACTGTATGGTGAGAAGTGCAAAGCTGCTTTTGATTCTGTCAG
CAAAAGTGTACAAACACTGCAGGGGCTTCGTAGAGTTTTGATGAGCTACCTCCATCAGAAAAAATTCAAATTTTTCA
TCAGGTGCCTCAAGATGTGTGGTCTCAAAGACCCCAAACAATTGTTATGGTTGTGCAACTACATTTGTCACTCAAT
GTCTTGAGATTCTTCAAGTGCTGTCGAAGCATCCAAGATCTAGAAAACAACTTGTTGCAGCTGGTATTTTGTCTGA
GTTGTTTGAAAACAATATTCACCAAGGTCCGAAAACAGCCCGTGCTCAAGCTAGAGCAGCTCTTTCTACTTTCTCG
GAGGGTGATCTGAGTGCAGTGAATGAGTTAAACAATCTGGTACAGAAGAAAATAATGTACTGCCTTGAACACCATC
GTTCCATGGATATTGCGCTCGCCACTCGTGAGGAAATGTTGTTGCTTTCAGAAGTTTGCTCTCTCACTGATGAATT
CTGGGAGTCAAGACTGCGCCTTGTTTTTCAGCTACTATTTTCATCCATTAAATTGGGTGCTAAACATCCTGCTATA
TCAGAGCACATAATTCTTCCTTGCCTTAAGATTATTTCTGTGGCATGTACACCTCCAAAACCAGATACGGCAGAGA
AAGAGCAAACGATGGGAAAATCTGCTCCTGCAGTGCAAGAAAAGGATGAGAATGCTGCTGGCGTCATAAAGTATTC
ATCAGAAAGCGAAGAAAATAACCTGAATGTATCTCAGAAGACGCGAGATATTCAGCTTGTGAGTTATTTGGAATGG
GAGAAAGGGGCATCATATCTCGACTTTGTGAGGAGGCAATACAAAGCCTCTCAGTCAATTAGAGGTGCGAGCCAAA
AGTCTAGAACCCACAGATCAGATTTCTTGGCACTTAAATATACACTTAGGTGGAAGCGACGGTCTAGCAGGACATC
TAAAGGTGGTTTACAGGCATTTGAGCTCGGGTCGTGGGTCACAGAGCTTATTCTTAGTGCATGTTCTCAATCTATC
AGGTCGGAGATGTGCACATTGATTAGTTTACTCGCTGCCCAAAGCTCACCTAGGCGCTACAGGCTGATTAATTTGC
TGATAGGCTTGCTTCCCGCTACGCTTGCAGCGGGTGAAAGTAGCGCAGAATACTTTGAGCTACTCTTCAAAATGAT
TGAGACACAAGATGCACTTCTTTTCTTAACTGTCCGGGGTTGTCTGACAACAATCTGTAAATTGATCTCCCAAGAG
GTGGGAAACATTGAATCTCTAGAAAGGAGTCTTCAGATTGACATTTCACAGGGATTTACCCTCCACAAGCTCTTAG
AACTCCTTGGAAAGTTCTTAGAGGTTCCAAATATTCGTTCCAGATTCATGAGAGACAATTTACTGTCTCATGTTCT
GGAGGCCCTCATTGTAATTCGTGGCTTGATAGTGCAAAAGACAAAGCTTATAAATGATTGCAACAGACGCCTAAAA
GATCTTCTTGACGGACTTCTGCTTGAAAGCAGTGAAAATAAACGTCAATTTATCCGTGCCTGTGTTTCTGGACTGC

AAACCCATGCAGAGGAGAACAAAGGACGTACCTGTTTGTTTATTCTTGAGCAACTTTGTAATCTGATCTGCCCGTC
AAAACCTGAGGCTGTGTATATGCTGATCTTGAACAAATCACACACGCAAGAGGAGTTTATCCGGGGATCAATGACA
AAAAATCCATATTCGAGTGCTGAGATTGGCCCACTGATGCGGGATGTGAAAAACAAGATTTGTCAGCAGTTGGACC
TGCTAGGTCTACTGGAAGATGACTATGGCATGGAGTTGCTTGTAGCAGGAAACATCATTTCACTAGACCTGAGCAT
AGCTCAAGTTTATGAGCTAGTTTGGAAGAAATCAAATCAGTCTTCTACTTCACTGACCAACTCCGCACTGTTAGCT
TCAAATGCAGCTCCCAGCAGGGATTGCCCTCCCATGACGGTGACATACAGACTTCAGGGGTTAGATGGTGAAGCTA
CTGAGCCTATGATCAAGGAATTGGAAGAAGATAGAGAAGAATCACAAGATCCAGAGATTGAGTTTGCAATAGCAGG
TGCAGTTCGAGAATATGGTGGTCTGGAAATTTTACTTGATATGATCAAGAGTTTACAAGATGACTTCAAATCCAAC
CAAGAAGAGATGGTTGCAGTTCTTGATCTCCTAAACCATTGCTGCAAGATTAGAGAGAACAGGCGAGCTCTACTCA
GGCTAGGGGCTTTAAGCTTACTTCTTGAAACAGCTAGGAGGGCATTTTCTGTGGATGCTATGGAGCCAGCTGAAGG
CATACTCTTAATTGTCGAGAGTTTGACGCTTGAAGCAAATGAAAGTGACAGCATCAGTGCCGCACAAAGTGCTTTG
ACTGTCAGTAACGAGGAAACTGGAACTTGGGAACAGGCCAAAAAAATAGTGCTTATGTTCTTAGAAAGGCTAAGCC
ATCCTTCAGGGCTTAAAAAGTCAAACAAACAGCAAAGGAACACGGAGATGGTTGCTAGAATCTTACCTTACTTAAC
ATACGGTGAGCCTGCAGCGATGGAAGCACTCATAGAGCATTTTAGCCCTTACCTGCAAAACTGGTCTGAATTTGAT
CAGCTTCAGCAACGTCATGAGGAGGACCCCAAGGATGACAGCATTGCTCAGCAAGCGGCGAAGCAGAGGTTTACTG
TGGAGAATTTTGTAAGAGTGTCAGAATCTCTCAAGACAAGTTCATGTGGAGAGAGGTTAAAAGACATAGTTTTGGA
GAATGGTATCATAGCTGTTGCTGTCAAACACATAAAGGAAATTTTTGCAATCACGGGACAGACTGGGTTCAAATCT
AGCAAGGAATGGCTTTTGGCTCTTAAACTTCCGTCAGTGCCTCTTATTTTGTCAATGCTAAGAGGATTGTCGATGG
GTCATTTGCCTACCCAAACATGCATAGACGAGGGAGGAATTTTAACCCTACTTCATGCCTTAGAAGGAGTCTCTGG
AGAAAACGACATCGGTGCAAGGGCTGAAAATTTGCTAGATACACTTGCCGATAAAGAAGGAAAAGGAGATGGATTT
CTTGGAGAGAAAGTCCGTGCATTACGAGATGCCACCAAAGATGAAATGAGACGCCGTGCATTAAGAAAGAGAGAGG
AGCTCCTGCAGGGACTTGGAATGCGTCAAGAGCTATCTTCTGATGGTGGTGAAAGGATTGTGGTTTCTCAACCAAT
TCTTGAAGGTTTTGAGGATGTAGAGGAAGAAGAAGATGGATTGGCTTGCATGGTGTGTAGAGAAGGCTACAAGCTC
AGACCTTCTGATTTATTAGGAGTCTACTCTTACAGCAAACGGGTGAATCTCGGTGTTGGCAATTCTGGAAGCGCAC
GTGGTGAATGCGTTTACACTACAGTGAGCTACTTCAACATAATTCATTTTCAATGTCATCAAGAGGCAAAAAGAGC
CGACGCTGCTCTTAAAAATCCGAAGAAAGAATGGGAAGGAGCTATGTTGCGTAACAATGAATCTCTTTGCAACTCT
CTCTTCCCTGTCAAGGGTCCCTCAGTTCCCTTAGCACAGTATCTTCGTTACGTTGACCAGTACTGGGATAATCTCA
ATGCTCTTGGTCGAGCCGACGGAAGCAGACTCCGTCTATTGACGTACGACATTGTCCTGATGCTGGCTCGGTTTGC
AACAGGAGCTTCTTTTAGCGCGGATTGCCGAGGTGGAGGAAGAGATAGCAATTCCCGTTTCTTGCCTTTCATGTTC
CAAATGGCCCGTCATCTTCTCGACCAAGGAGGCCCCGTGCAACGCACAAACATGGCTAGATCTGTTTCATCGTACA
TTTCATCATCATCGACATCTACAGCAACAGCGCCATCCTCGGACTCGCGACCTTTAACTCCTGGATCCCAGCTATC
ATCAACTGGAACAGAGGAAACGGTTCAGTTCATGATGGTAAACTCGCTTCTATCAGAATCATATGAATCATGGCTC
CAACACCGTAGGGTCTTTCTCCAGCGGGGGATTTACCATACTTTCATGCAACACGCTCACGGTCGGGTAGCTTCTC
GCGCTGCAGAACCAACCAGTTCAGGAGGCAAAACCCAAGACGCAGAAACCTTAACCGGCGATGAACTTCTCTCTAT
CGTCAAACCAATGCTGGTCTACACCGGTATGATCGAACAGCTTCAACAGTTGTTCAAACCCAAGAAACCGGTCCAC
ATCGAACCAATCAAGAAAGAAGGTACATCAAGTGGAGTAGAGCTTGAGCCATGGGAGATTGTAATGAAAGAGAAGC
TGCTGAATGTGAAAGAAATGATTGGTTTCTCCAAGGAATTGATCTCGTGGCTCGACGAAATCAACTCTGCAACTGA
TCTACAAGAAGCATTTGATATTGTCGGCGTTTTGGCTGATGTTTTGTCTGAAGGAGTTACACAGTGTGATCAGTTT
GTGAGATCGGCGATCGATAAAGATTGAtttcgggaacaaggaagagctttatgtgtttttatttttggcaatgtgtc
tcttgttattaatatttgtgattgattgtaatttagaaaattgtcgttgttgtaaacattgagacactctgttttg
ttgtgttattaatgcaaaagcacacacatattgctctttgtttaacctcgcagcacaacaactcgcacgatcgagt
cttcggtttgaaaatttccacatttggttttggtatcatggtataacattttatgcaaatgtgtagattcggattg
gattagtgtacaggttcgaattttaatcatcaaaaatataagtttggtttattgagccaccg
